# Recent Advancements in Nickel-Catalyzed Electrochemical Reductive Cross-Coupling

**DOI:** 10.1021/acsorginorgau.5c00056

**Published:** 2025-07-10

**Authors:** Subban Kathiravan, Ian A. Nicholls

**Affiliations:** Bioorganic & Biophysical Chemistry Laboratory, Linnaeus University Centre for Biomaterials Chemistry, Department of Chemistry & Biomedical Sciences, 4180 Linnaeus University, Kalmar SE-39182, Sweden

**Keywords:** nickel catalysis, electrochemical synthesis, reductive cross-coupling, C−H activation, C−C, C−N, C−S, and C−P bond formation, sustainable chemistry, transition-metal catalysis, organic electrochemistry

## Abstract

Nickel-catalyzed electrochemical cross–coupling has emerged as an important advancement in synthetic chemistry, combining the versatile catalytic properties of nickel with the sustainability and precision of electrochemical methods. This review captures the recent progress in this dynamic field, focusing on developments published from 2015 onward, and emphasizes the development of innovative catalytic systems and reaction conditions that enhance efficiency, selectivity, and environmental sustainability. Key advancements include novel nickel catalysts, expanded substrate scopes, and mechanistic insights that elucidate the synergistic benefits of electrochemical approaches. By exploring these recent developments, we highlight the transformative potential of nickel-catalyzed electrochemical cross–coupling in facilitating complex bond formation under mild conditions. This comprehensive overview provides a foundation for understanding the current state and future directions of this promising area, emphasizing its significance in advancing green and efficient synthetic methodologies.

## Graphical abstract

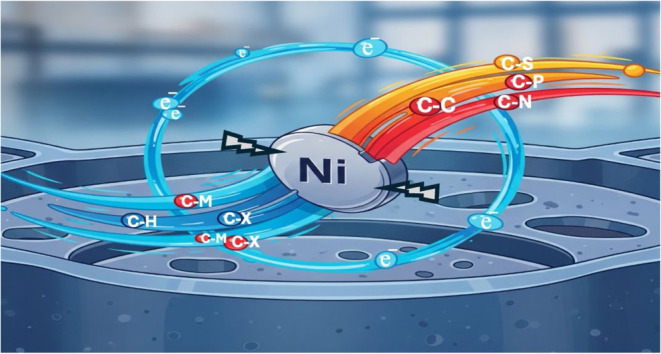

## Introduction

Modern electrochemical techniques have revolutionized organic synthesis, providing a more sustainable and versatile approach to many chemical transformations.^1–3^ Traditionally, organic synthesis has relied on stoichiometric reagents, often involving toxic or hazardous substances and generating significant waste. In contrast, electrochemical organic synthesis harnesses electrical energy to drive reactions, offering a cleaner and more efficient alternative.^4–7^ By using electrons as direct reagents, this technique reduces the need for hazardous chemicals and harsh conditions while enhancing reaction efficiency, selectivity, and control over reaction pathways.

As interest in green chemistry continues to grow, electrochemical synthesis is emerging as a key tool for developing more sustainable and environmentally friendly chemical processes.^8,9^ This approach introduces several advantages, including milder reaction conditions, improved atom economy, and enhanced selectivity. Additionally, electrochemical methods have expanded the synthetic toolbox, enabling the discovery of new reaction pathways and the selective functionalization of complex molecules.^10,11^ As a result, electrochemical synthesis is playing a crucial role in modern organic chemistry, driving innovation in the production of pharmaceuticals, materials, and other valuable organic compounds.^12^

## Nickel-Catalyzed Electrochemical Organic Synthesis

Nickel-catalyzed electrochemical cross-coupling has emerged as a transformative approach in modern synthetic chemistry, offering sustainable and efficient pathways for the formation of carbon–carbon and carbon-heteroatom bonds.^13–15^ The field has witnessed rapid advancements due to the unique ability of nickel to facilitate challenging bond formations under mild conditions.^16^ This, combined with the intrinsic advantages of electrochemical techniques, such as the avoidance of stoichiometric chemical oxidants or reductants and improved reaction selectivity, has driven a surge of interest and significant breakthroughs in recent years. Nickel’s diverse oxidation states and relatively low cost make it particularly well-suited for electrochemical applications, where it can engage in various catalytic cycles to facilitate cross-coupling reactions. Electrochemical methods complement nickel’s catalytic ability by enabling precise control over redox processes, opening new avenues for reaction innovation and mechanistic understanding.

This Perspective aims to encapsulate the recent developments in nickel-catalyzed electrochemical cross-coupling, with a focus on key advancements in reaction design, mechanistic insights, and synthetic applications. We will explore the integration of nickel catalysis with electrochemical techniques, discussing how this synergy has been leveraged to enhance the reaction efficiency, selectivity, and sustainability.

The review is structured around three key areas of focus:

### Enantioselective Nickel Catalysis

We first discuss electrochemically driven enantioselective nickel catalysis and its application in organic synthesis, highlighting the advances in chiral molecular scaffolds.

### Cross-Electrophilic Coupling

Next, we explore cross-electrophilic synthesis, emphasizing the importance of these reactions in constructing complex molecular architectures. Although the pioneering reports on nickel-catalyzed electrochemical cross-coupling, initially developed in the 1990s,^15,21–23,25,38^ are not covered in exhaustive detail in this review, their innovations laid the groundwork for many of the transformations discussed herein. These early contributions established key mechanistic principles and catalytic strategies that continue to serve as a foundation for the ongoing development of nickel-catalyzed electrochemical cross-coupling methodologies. For readers interested in the pioneering contributions and foundational advances, we encourage readers to refer to the original literature cited in this review.

### C–H Activation Reactions

Finally, we delve into nickel-catalyzed C–H activation reactions, particularly for C–N bond formation, showcasing the potential of these methods in expanding the scope of nickel-catalyzed electrochemical cross-coupling.

By providing a comprehensive overview of the state-of-the-art of this burgeoning field, we aim to underscore the transformative potential of nickel-catalyzed electrochemical cross-coupling in organic synthesis. Our discussion will also illuminate the current challenges and future directions, emphasizing how continued research and development can further solidify this approach as a cornerstone of modern chemical synthesis.

While this review focuses on advances in nickel-catalyzed electro-organic synthesis since 2015, it is important to acknowledge earlier studies in electrochemical nickel catalysis, which provide essential context for understanding the foundational contributions and ongoing evolution of this rapidly developing area.^17–25^

## Nickel-Catalyzed Enantioselective Cross-Coupling

Enantioselective cross-coupling reactions have emerged as pivotal tools in the field of asymmetric synthesis, allowing for the creation of chiral molecules with high precision and efficiency.^26,27^ Among the various transition metals utilized in these processes, nickel (Ni) has garnered significant attention due to its unique reactivity and versatility. Nickel-catalyzed enantioselective cross-coupling reactions are increasingly recognized for their ability to generate a diverse array of chiral compounds, which are crucial for applications in pharmaceuticals, agrochemicals, and materials science.^28,29^ Chiral molecules are fundamental in pharmaceuticals due to their potential to interact selectively with biological targets, often resulting in drugs with enhanced efficacy and reduced side effects. However, achieving high enantioselectivity in synthetic processes is challenging, requiring catalysts that can effectively control the stereochemistry of the reaction. Nickel, as a catalyst, has gained significant attention for its unique reactivity and versatility in this context.

Nickel offers several advantages over traditional catalysts like palladium and platinum.^30^ It is relatively inexpensive and abundant and exhibits a broad range of oxidation states, facilitating various types of bond-forming reactions. Nickel’s capability to engage in multiple coupling reactions, such as cross-coupling, reductive coupling, and oxidative coupling, makes it an attractive choice for synthetic chemists seeking to develop novel methodologies.^13^

Recent years have seen substantial progress in nickel-catalyzed enantioselective cross-coupling.^31^ The development of chiral nickel complexes and ligands has been particularly influential in enhancing the enantioselectivity of these reactions.^32^ Chiral ligands, such as phosphines, amines, and other functionalized molecules, play a critical role in dictating the stereochemical outcome of the cross-coupling process.

Guo and co-workers have reported an asymmetric electrosynthesis combined with a chiral Ni catalyst for an intermolecular alkylation reaction in an undivided cell.^33^ The reaction gave good yields with excellent enantioselectivities (up to 97% ee). The mechanistic studies suggested that single-electron anodic oxidation generates a Lewis-acid-bound radical intermediate, which selectively reacts with the benzylic radical species to give an alkylated product (Scheme 1). While the reaction demonstrated promising enantioselectivity with highly substituted phenol coupling partners, it showed reduced performance with less substituted phenols, resulting in only moderate yields and enantioselectivities. These findings highlight a sensitivity to electronic and steric factors of the phenol coupling partner, with more substituted derivatives delivering higher yields and enantioselectivity.

**Scheme 1:**
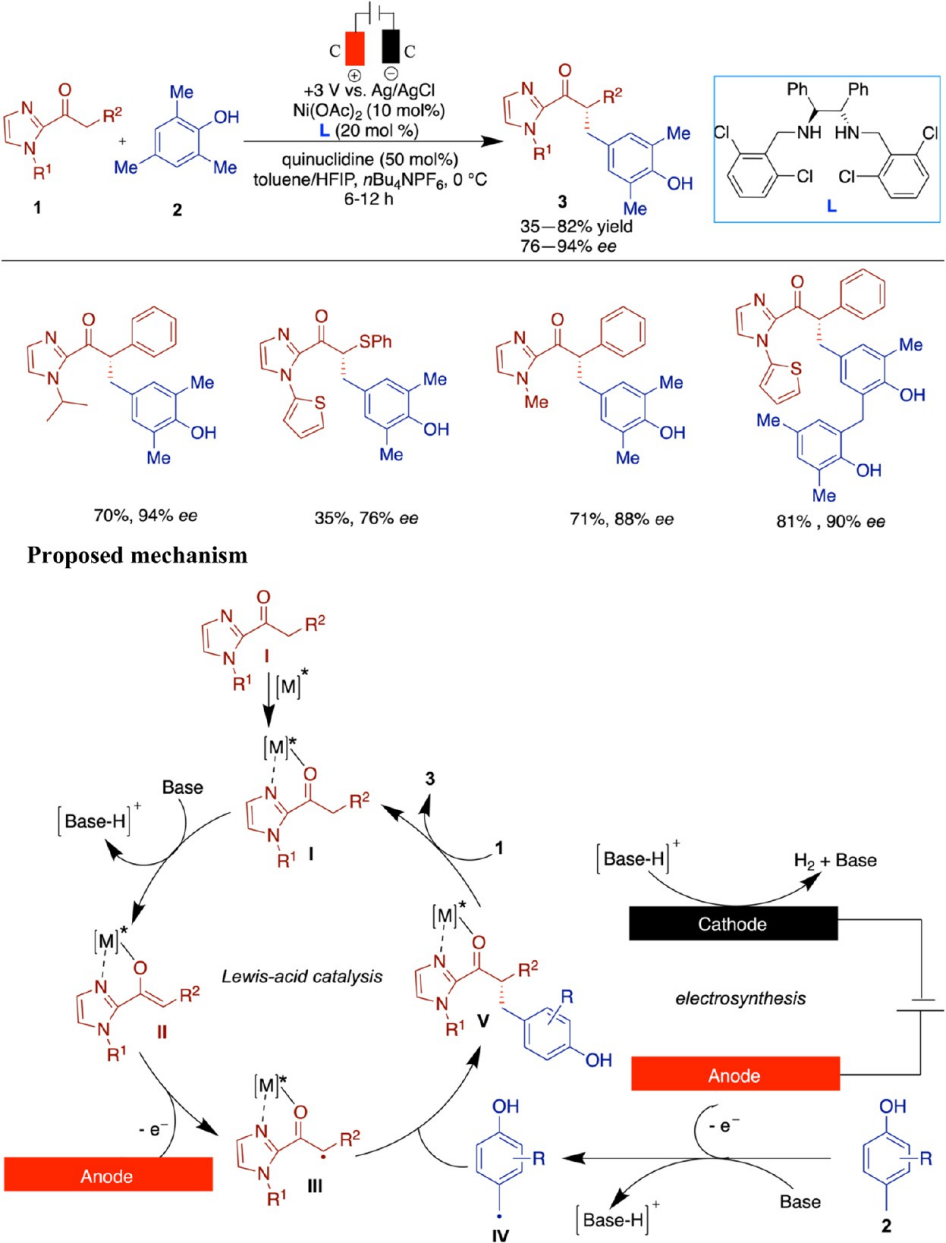
Nickel-Chiral Diamine Catalyzed Asymmetric Electrochemical Alkylation

The same group has reported enantioselective nickel-catalyzed electrochemical radical allylation through single-electron transfer (SET) (Scheme 2).^34^ The activation of nucleophiles via nickel catalysis initiated a single-electron transfer process, resulting in the formation of a chiral catalyst-bound radical cation intermediate. This intermediate can be utilized as an alternative approach for creating stereocontrolled radical reactions. Employing a suitable chiral diamine-based nickel complex can act as the essential chiral catalyst to initiate the anodic single-electron transfer (SET) activation of an enolate species. This catalyst simultaneously facilitates a cross-coupling pathway, enabling an effective asymmetric induction for Keck radical allylation. Cyclic voltammetry experiments corroborated the proposed SET activation processes, showing that the nickel-bound enolate intermediate has a lower oxidation potential and is more prone to anodic oxidation in comparison to the other reaction partners. These findings illustrate the potential for developing stereocontrolled radical reactions. In this example, all reported reactions afforded only moderate yields, with the highest being 75%, and most in the range of 49–60%. Additionally, optimal results required conducting the reaction at −10 °C. These observations suggest that, while the reaction is enantioselective and operationally viable, the yields remain moderate across substrates, and the need for low-temperature conditions reflects the typical trade-off in achieving high stereocontrol. The authors proposed a mechanism for the observed reactivity based on the experimental findings. The nickel-enolate intermediate (**II**) was formed through the condensation of 2-acylimidazoles with a nickel catalyst. The nickel-coordinated radical cation intermediate (**III**) was generated via anodic oxidation of (**II**) through the single-electron transfer (SET) pathway, which plays a significant role in the asymmetric induction within the catalytic cycle. The key chirality-generating step involves the exergonic addition of this anodically generated electrophilic nickel-coordinated radical cation to the double bond of the electron-rich allylstannane (**2**), forming a stereogenic center in the intermediate (**IV**). Following the intermolecular SET event, the subsequent β-scission and anodic oxidation process yield the tin cation and intermediate (**V**). Finally, the desired product (**3**) is released, allowing the catalytic cycle to restart.

**Scheme 2:**
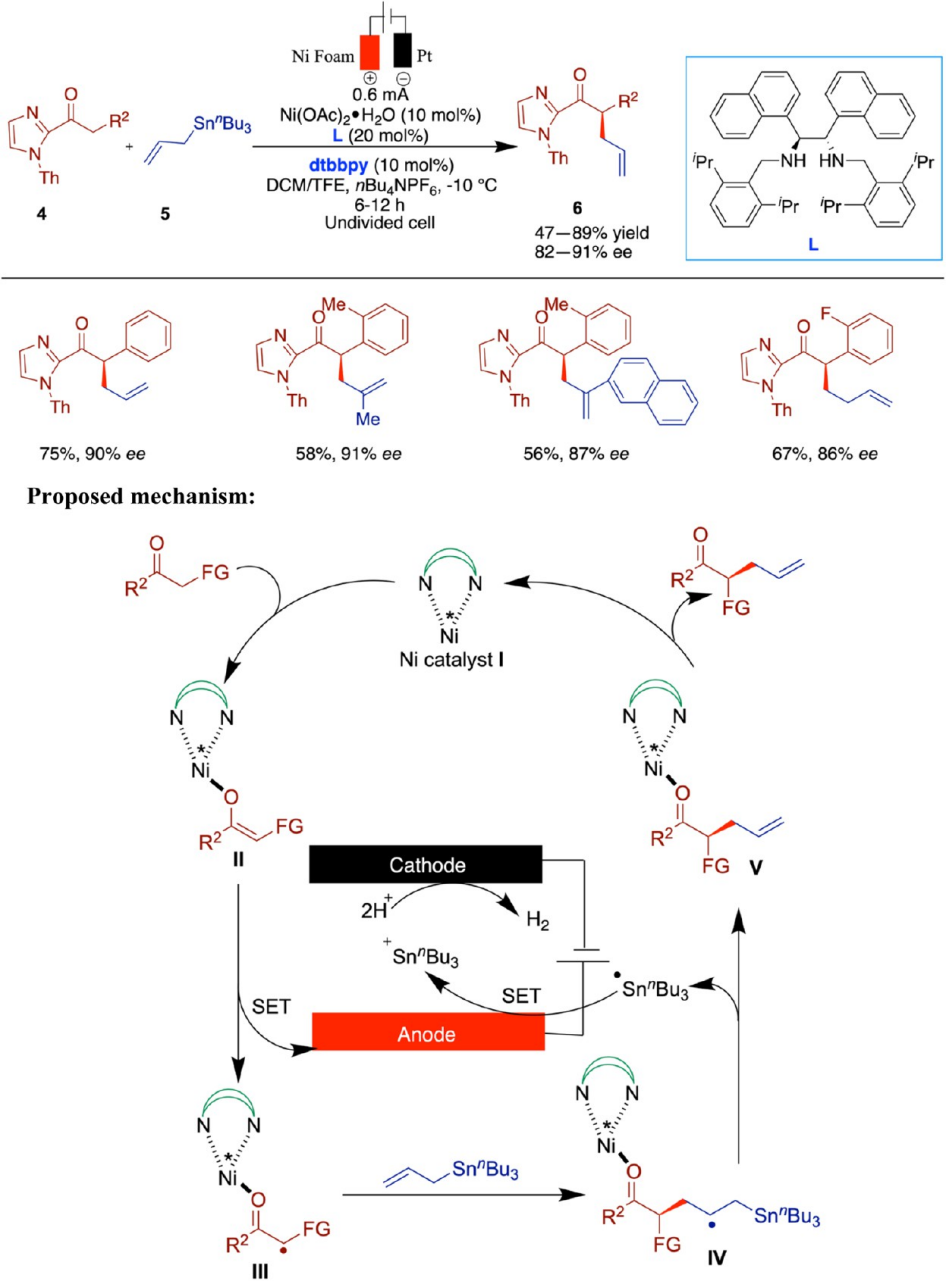
Enantioselective Nickel-Catalyzed Electrochemical Radical Allylation

The group has extended their elegant work on nickel electrocatalysis and has very recently reported enantioselective nickel-catalyzed anodic oxidative dienylation and allylation reactions for an innovative approach to achieve high stereocontrol in radical reactions through electricity-driven asymmetric Lewis-acid catalysis (Scheme 3).^35^ This method leverages the unique properties of chiral Lewis acids to facilitate both asymmetric dienylation and allylation reactions, leading to the formation of all-carbon quaternary stereocenters. The approach demonstrates significant potential in the modular synthesis of functional and chiral benzoxazole-oxazoline (Box) ligands. The study addresses the challenge of controlling stereochemistry in radical reactions, which is often hindered by incident racemization reactions. By merging electrochemistry with asymmetric Lewis-acid catalysis, the researchers developed a strategy to generate α-carbonyl radical species from enolate intermediates. This methodology offers a versatile and universally applicable strategy for various fields including electrosynthesis, organic chemistry, and drug discovery. Key to the success of this approach is the coordination of the chiral catalyst with the substrate, which lowers the oxidation potential and facilitates the electrocatalytic process while reducing the likelihood of racemic reactions. The ability to adjust the applied potential or current density at the anode enables precise control over the electron transfer, which enhances the selectivity and reaction rates. This control allows for the preferential activation of catalyst-bound substrates, while leaving unbound substrates largely unaffected. Structurally customizable Lewis-acid–nickel complexes provide a well-defined chiral environment that enables high levels of asymmetric induction. Detailed mechanistic investigations, including cyclic voltammetry and control experiments, revealed that the presence of the nickel catalyst significantly lowers the oxidation potential of the substrate. This facilitates the formation of a catalyst-bound reactive intermediate that plays a key role in the oxidation process. Notably, the reaction maintained high enantioselectivity even at low catalyst loadings, indicating the chiral nickel complex’s ability to suppress racemic background pathways.

**Scheme 3:**
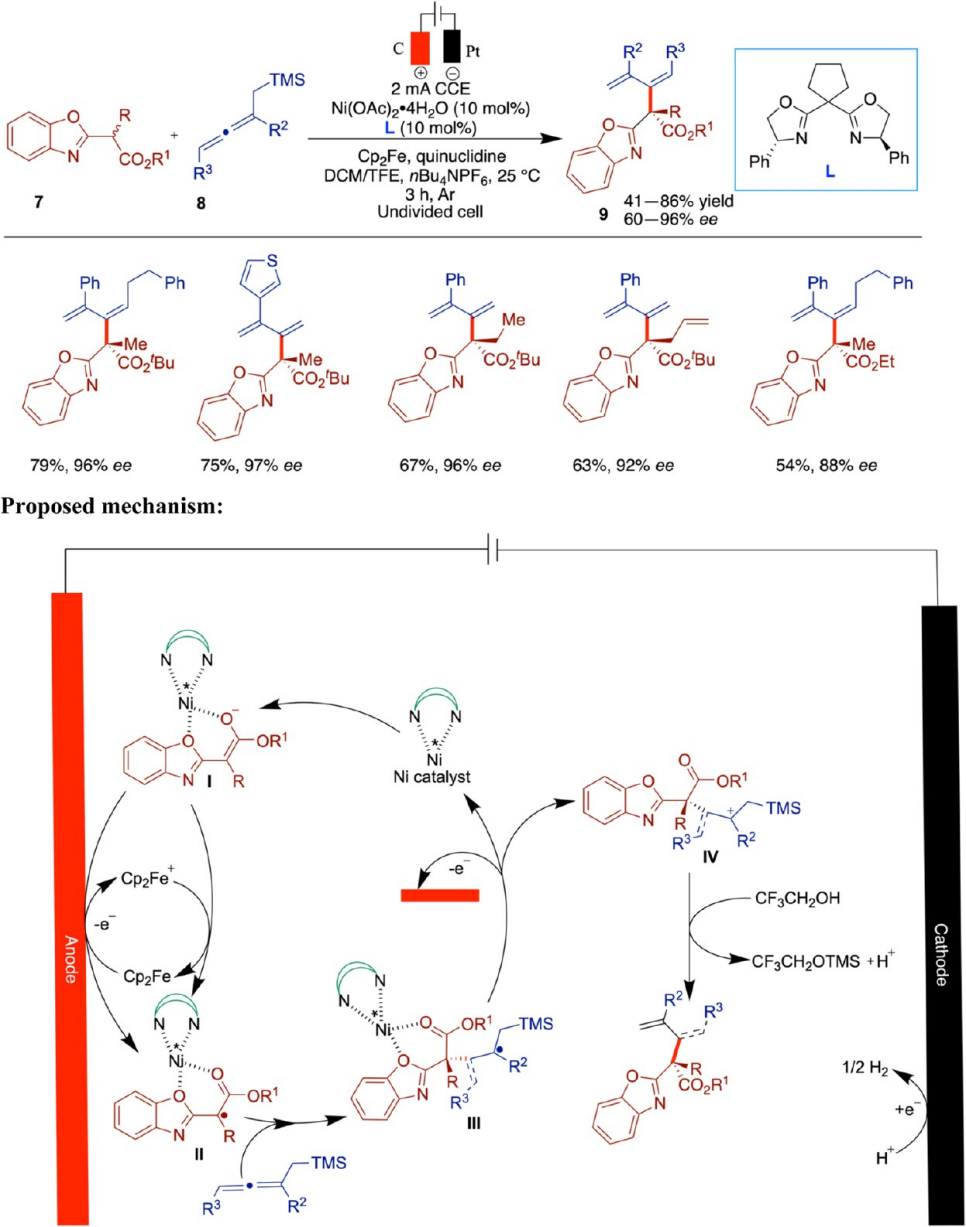
Enantioselective Nickel-Catalyzed Anodic Oxidative Dienylation and Allylation

The generality of the method was demonstrated through the synthesis of nearly 60 compounds, showcasing broad applicability. Most products were obtained in moderate to good yields, with a few examples delivering lower yields (down to 41%). Importantly, the enantioselectivity remained consistently high across the entire substrate scope, regardless of variations in yield.

The authors proposed a mechanistic pathway involving constant current electrolysis (CCE), wherein interaction between the substrate and the nickel catalyst forms a nickel-bound enolate intermediate (**I**), which undergoes preferential anodic oxidation. A ferrocene-assisted single-electron transfer converts intermediate **I** into a nickel-coordinated radical species (**II**), which is subsequently trapped by allenylsilane, generating a new stereogenic center in intermediate **III**. According to DFT studies, this radical intermediate is further oxidized to a cationic species (**IV**), followed by TMS removal to yield the final product.

Mei and co-workers have reported the enantioselective nickel-catalyzed electrochemical synthesis of BINOL derivatives through reductive homocoupling of aryl bromides using chiral pyridine-oxazoline ligands in an undivided cell (Scheme 4).^36^ The authors demonstrated the critical role of electrochemistry in enhancing the reactivity by conducting control experiments using conventional metal reductants. When manganese powder was used in place of electrochemical reduction, the desired product was obtained in 24% yield with 89% ee. Similarly, replacing the electric current with zinc powder resulted in only 5% yield and 86% ee. These results underscore the superior efficiency and selectivity achieved through the electrochemical approach in combination with nickel catalysis. The authors carried out a systematic ligand screening to optimize the enantioselective electrochemical homocoupling reaction. While common ligands such as BINAP and pybox were ineffective, modifications to pyrox-type ligands significantly improved both the yield and enantioselectivity, with the best result reaching 93% ee. The substrate scope included naphthyl bromides bearing alkyl, ether, ester, alkenyl, boron, and halogen groups, which were well tolerated and generally gave good yields with high enantioselectivities. In contrast, more challenging substrates, such as 8-bromoquinoline and ester-functionalized aryl bromides, gave lower yields and reduced ee. Control experiments using Mn as a reductant led to decreased yields, highlighting the superior performance of the electrochemical setup. Notably, when sterically bulky substituents were introduced on the naphthyl bromide component (**10**), a drop in yield was observed, likely due to increased steric hindrance affecting the coupling efficiency. The authors have proposed a mechanism, presented in Scheme 4, which is based on Amatore’s work on the electroreductive nickel-catalyzed dimerization of aryl halides.^37^ The proposed mechanism begins with the cathodic reduction of a Ni­(II) precatalyst to generate a Ni(0) species (**A**). This Ni(0) species undergoes oxidative addition with an aryl halide to form an aryl-Ni­(II) complex (**B**). Further electrochemical reduction converts this intermediate into an aryl–Ni­(I) species (**C**). A second oxidative addition with another aryl halide then produces a Ni­(III) complex (**D**), which undergoes reductive elimination to release the biaryl product and regenerate the Ni­(I) species (**E**). Finally, the Ni­(I) complex is reduced at the cathode to regenerate the active Ni(0) catalyst, completing the catalytic cycle.

**Scheme 4:**
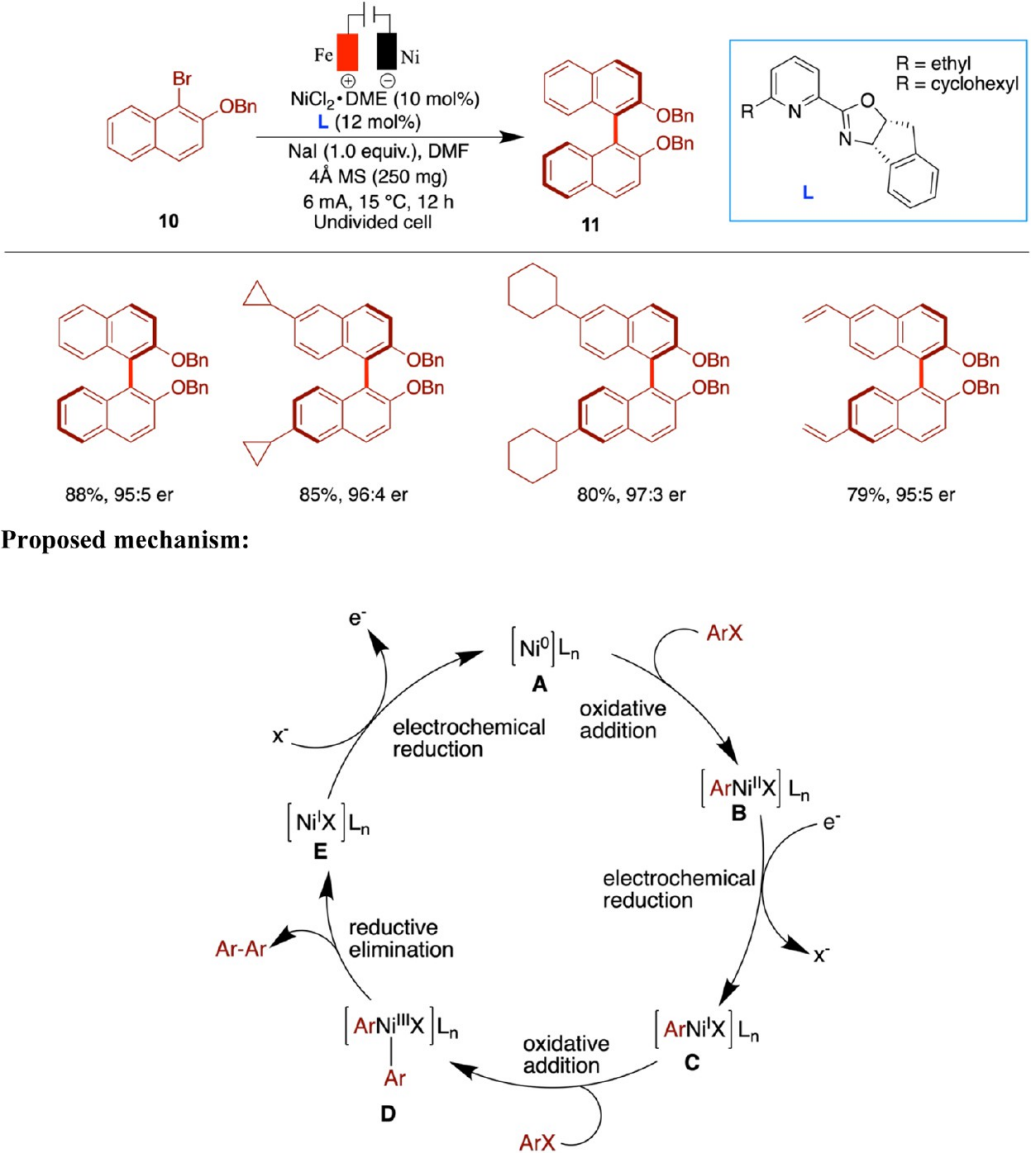
Enantioselective Nickel-Catalyzed Electrochemical Synthesis of Binol Derivatives

## Enantioselective Cross-Coupling with Alkyl Halides

While the primary focus of this review is electrochemical nickel-catalyzed cross-coupling reactions developed after 2015, it is important to acknowledge early seminal contributions that laid the groundwork for this field. Durandetti and co-workers pioneered the nickel-catalyzed electrochemical cross-coupling of aryl halides with activated alkyl halides under racemic conditions, using a sacrificial anode in an undivided electrochemical cell. First reported in 1996, this approach provided an efficient and practical method for C­(sp^2^)–C­(sp^3^) bond formation under mild conditions, eliminating the need for stoichiometric reductants such as zinc or manganese powders (Scheme 5).^38^ However, note that the use of a sacrificial anode still results in metal waste, which must be considered when evaluating the overall sustainability of the process. Through careful optimization of the catalytic system, the authors identified NiBr_2_(bpy) as a crucial component for enabling efficient cross-coupling. The reaction was conducted in an undivided (one-compartment) electrochemical cell using aluminum or zinc rods as the anode and nickel foil, stainless steel sponge, or carbon fiber as the cathode. The aryl halide was added via a syringe pump. As reported, this setup delivered yields ranging from 44 to 70% and demonstrated notable practicality due to the use of an undivided electrochemical cell, inexpensive and commercially available electrode materials, and mild operating conditions. For reference, reactions were conducted on a 10 mmol scale with 1 mmol of the catalyst. Notably, a variety of activated alkyl halides, including allyl derivatives, were successfully employed, marking an important advance in the development of electrochemical nickel-catalyzed cross-coupling. For detailed reaction conditions and mechanistic insights, readers are referred to the original publication.

**Scheme 5:**
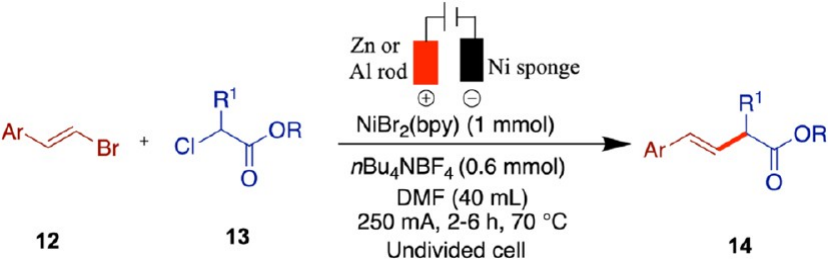
Nickel-Catalyzed Electrochemical Cross-Coupling

In a mechanistically related transformation, Reisman and co-workers developed an enantioselective nickel-catalyzed electrochemical cross-coupling between alkenyl electrophiles and benzylic halides (e.g., α-methylbenzyl chloride) using a sacrificial zinc anode (Scheme 6).^39^ While the substrates differ from the activated alkyl halides used in Durandetti’s (Scheme 5) and Mei’s (Scheme 7), the study demonstrates the potential for enantioselective C­(sp^2^)–C­(sp^3^) bond formation under electrochemical conditions using chiral ligands. A broad range of coupling partners was successfully employed, including styrenyl and alkyl-substituted alkenyl bromides, benzylic chlorides, and functionalized electrophiles such as aryl boronates, protected alcohols, and heterocycles. The reaction exhibited excellent tolerance to electron-donating and electron-withdrawing groups, halogens, and polar functionalities, consistently affording high enantioselectivities. However, limitations were observed: Substrates containing aryl bromides were prone to side reactions, including iodination and loss of the bromine through hydrodebromination, and the ortho-methyl-substituted product was obtained in lower yield and enantiomeric excess compared to its ortho-methoxy counterpart. Increased steric bulk on the alkyl chain or in ring size also led to diminished selectivity in some cases.

**Scheme 6:**
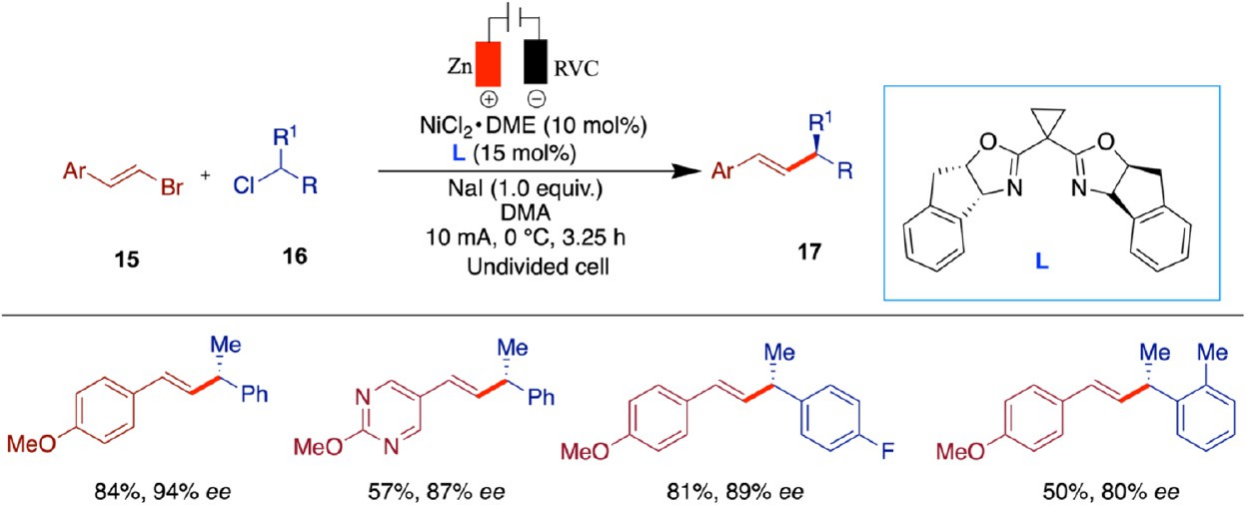
Enantioselective Electrochemical Nickel-Catalyzed Cross-Electrophile Coupling

**Scheme 7:**
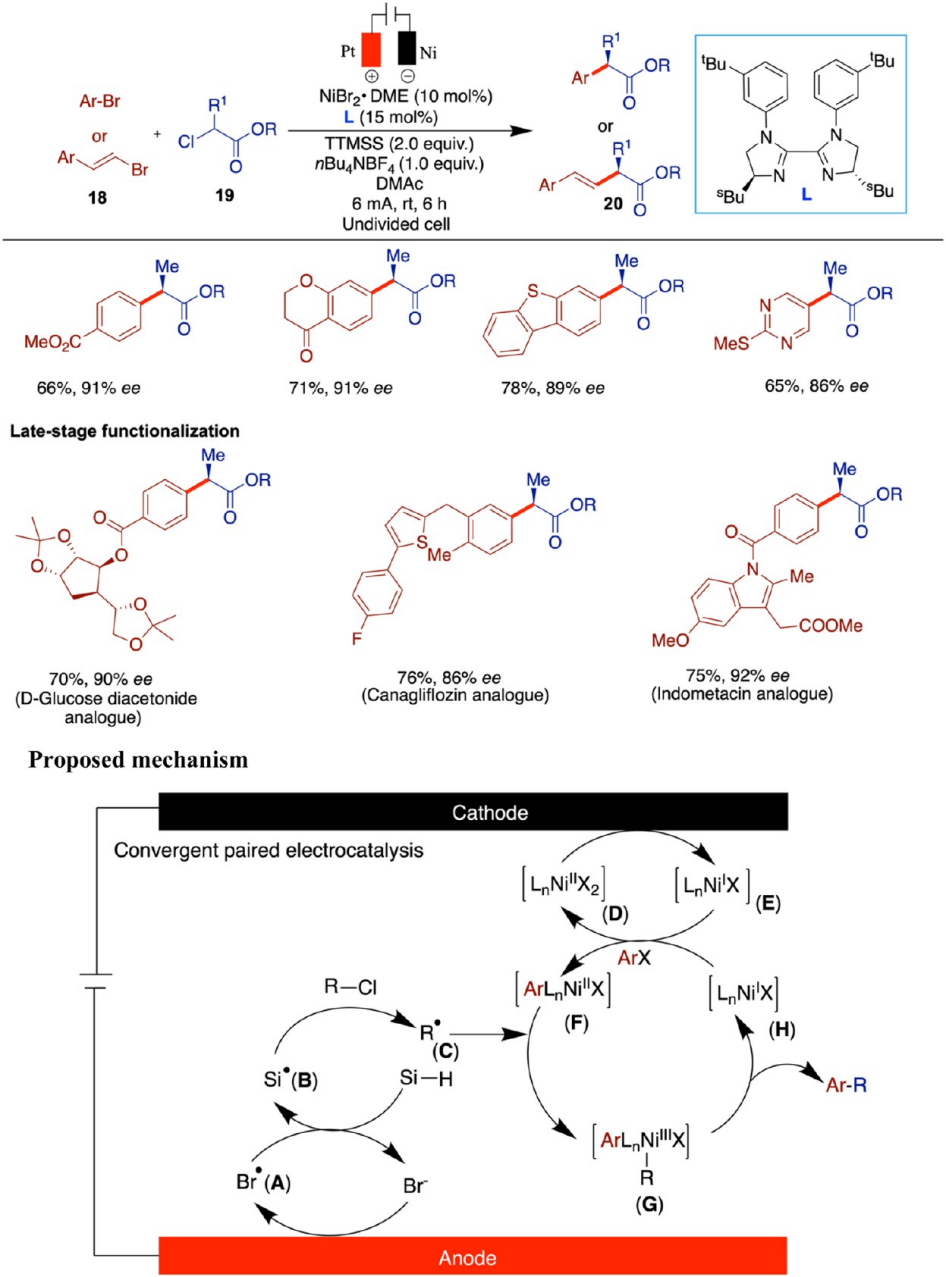
Electrolysis-Enabled Nickel-Catalyzed Enantioselective Reductive Cross-Coupling

Followed by these advances, the Mei group reported a paired electrolysis-enabled nickel-catalyzed enantioselective reductive cross-coupling between α-chloroesters and aryl bromides using chiral bis-imidazolidine ligands (Scheme 7).^40^ In this study, the authors screened a range of chiral ligands and showcased a broad and effective substrate scope. Although many substrates performed well under the optimized conditions, certain activated α-chloroesters afforded lower yields. Furthermore, the utility of the method was showcased through its application in the late-stage functionalization of the NSAID derivatives. In this study, the authors employed a paired electrolysis strategy, where both the anode and the cathode actively participate in the reaction. The process begins with anodic oxidation of the bromide ion to generate a bromine radical (**A**), which promptly abstracts a hydrogen atom from the silane, forming a silyl radical (**B**). This radical then reacts with the α-carbonyl chloride via chlorine atom abstraction, yielding an electrophilic alkyl radical intermediate (**C**). Simultaneously, the Ni­(II) complex (**D**) is reduced at the cathode to a Ni­(I) species (**E**). Subsequent oxidative addition involving another Ni­(I) species leads to the formation of a Ni­(II) intermediate (**F**). The alkyl radical can then be intercepted by the nickel complex (**F**), forming a high-valent Ni­(III) species (**G**), which undergoes reductive elimination to forge the desired C­(sp^2^)–C­(sp^3^) bond formation, while regenerating the Ni­(I) catalyst (**H**). It should be noted, as the authors mentioned, that an alternative pathway involving Ni(0)/Ni­(II)/Ni­(III)/Ni­(I) redox states cannot be ruled out. This unique approach has proven to be a valuable tool in organic synthesis, although the precise pairing of the rate and electric potential of anodic oxidation and cathodic reduction makes such processes difficult to achieve.

### Electrochemical Cross-Coupling of Aryl Bromides and Aziridines

Aziridines are well-known for their high reactivity in nucleophilic substitution reactions. Their inherent ring strain can be further exploited by introducing aryl or alkyl substituents on the ring, enhancing their synthetic utility. As a result, nickel-catalyzed arylation, alkenylation, and alkylation of aziridines have attracted considerable attention in the context of metal-mediated transformations.^41–43^ In 2013, three independent research groups of Rueping,^44^ Mei,^45^ and Nevado^46^ reported the use of aziridines in nickel-catalyzed electrochemical cross-coupling reactions. Notably, Mei, and Nevado developed enantioselective versions of these reactions, while Rueping’s approach yielded racemic products.

Rueping and co-workers developed a racemic nickel-catalyzed electrochemical strategy for the reductive cross-electrophile coupling between aziridines and aryl halides, affording phenethylamine derivatives under mild, room temperature conditions with good efficiency (Scheme 8).^44^ Importantly, this method successfully engaged unsubstituted *N*-tosylaziridines substrates typically difficult to functionalize. Mechanistic investigations, including evaluation of the nickel­(II) catalyst and electrode materials, provided valuable insight into the transformation’s underlying processes. The authors used stainless steel as both anode and cathode, and replacing the anode with zinc or aluminum resulted in no product formation. A variety of aryl electrophiles were screened, and it was found that sterically hindered substrates such as 2-bromo-1,3,5-trimethylbenzene gave no product, while 1-bromonaphthalene afforded only low yields. When more challenging unsubstituted aziridines were tested, the initially optimized ligand proved to be ineffective. However, the use of 4,4-bis­(trifluoromethyl)-2,2-bipyridine provided good to moderate yields with various aryl halides.

**Scheme 8:**
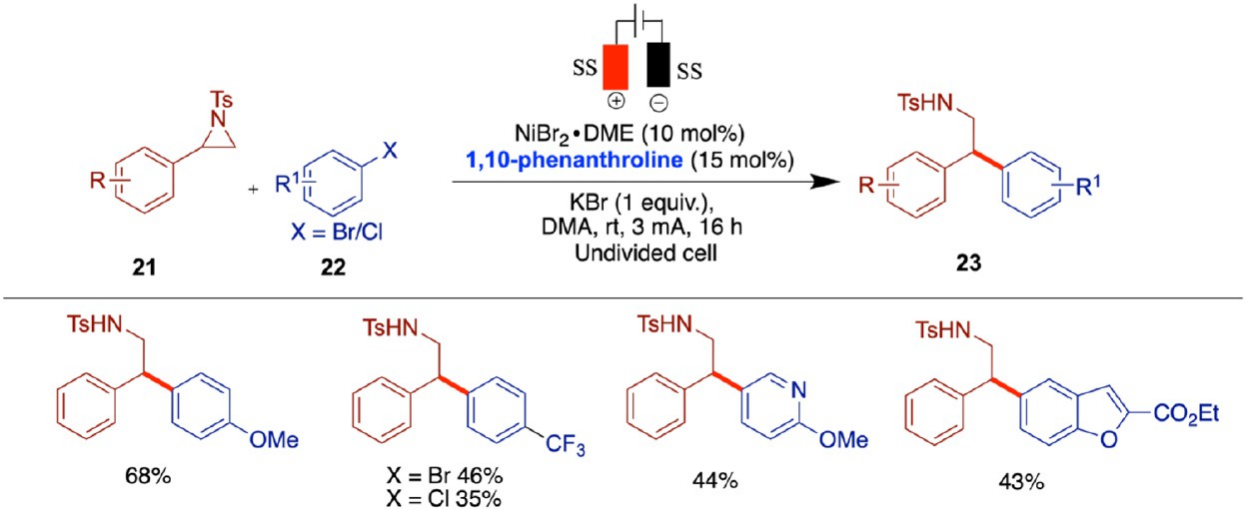
Electrochemical Reductive Cross-Coupling of Aryl Aziridines and Aryl Bromide/Chloride

Later that same year, the Mei research group has reported a nickel/biimidazole-catalyzed electrochemical enantioselective reductive cross-coupling of aryl aziridines with aryl iodides (Scheme 9).^45^ This process produces β-phenylethylamines with good to excellent enantioselectivity and broad functional group tolerance. The authors demonstrated that electroreduction efficiently promotes turnover of the nickel catalyst, as evidenced by a direct comparison with a conventional system using Mn powder activated by TMSCl. The electrochemical approach showed superior performance, highlighting its advantage over metal-reductant-mediated turnover. To enhance the reactivity and selectivity of the reaction, the authors investigated the role of the ligand structure through statistical analysis and compared it with ligands used in mechanistically related photochemical reactions. Notably, when aryl iodides bearing electron-withdrawing ester groups were employed, the reaction yielded lower product amounts, although high enantioselectivities (88–90% ee) were still maintained. The authors proposed a mechanism that begins with the cathodic reduction of the Ni­(II) precatalyst, generating the Ni(0) species **B**. Oxidative addition of aryl iodide to **B** forms the aryl–Ni­(II) intermediate **C**. Meanwhile, the nucleophilic iodide-induced ring opening of aziridine yields intermediate **E**, which may undergo a single-electron transfer (SET) to form radical species **F**. Alternatively, the formation of F via SET from a Ni­(I) species cannot be ruled out. The resulting radical is then intercepted by **C**, affording Ni­(III) intermediate **D**, which undergoes reductive elimination to give the cross-coupled product and regenerate the Ni­(I) species.

**Scheme 9:**
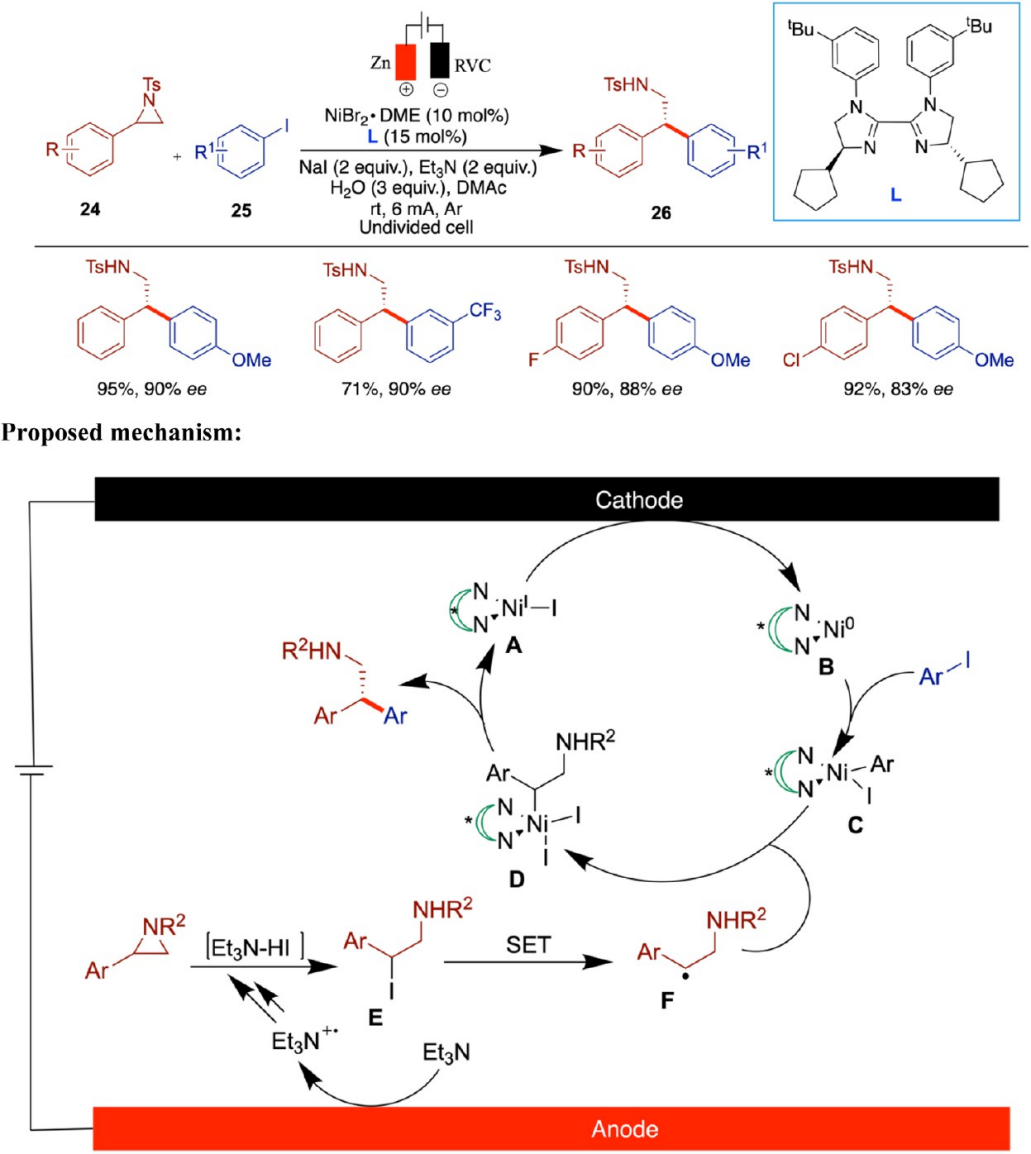
Nickel/Biimidazole-Catalyzed Electrochemical Enantioselective Reductive Cross-Coupling

Around the same time, Nevado et al. reported a similar electrochemical nickel-catalyzed enantioselective reductive cross-coupling of aziridines with alkenyl bromides (Scheme 10).^46^ This useful synthesis enabled the authors to produce enantioenriched β-aryl homoallylic amines with excellent E-selectivity. In both studies, the authors used triethylamine as a terminal reductant. Among the ligands screened, the chiral bisoxazoline ligand provided the highest enantioselectivity. The substrate scope was broad, demonstrating excellent functional group tolerance. A variety of N-tosyl- and Cbz-protected 2-aryl aziridines bearing electron-donating (e.g., −Me, −tBu, −OAc), halogen (−F, −Cl, −Br), and electron-withdrawing groups (−CF_3_, −COOMe, −CN) were well tolerated, delivering the desired products in moderate to good yields with high enantioselectivities. Sterically hindered ortho- and meta-substituted aziridines, as well as heteroaryl aziridines, also reacted efficiently. On the alkenyl bromide side, a wide range of styrenyl and vinyl bromides bearing diverse substituents including halogens, acetoxy, trifluoromethoxy, pinacol boronate, and heteroaromatics (pyridine and pyrimidine), were compatible. Notably, challenging substrates such as cyclic disubstituted alkenes required modified conditions. The utility of the method was further illustrated through the late-stage functionalization of drug-derived fragments such as estrone, naproxen, and d-alanine derivatives. Through a series of mechanistic studies, the authors determined that the process involves a stereoconvergent mechanism, where the aziridine is activated via nucleophilic ring opening. The authors proposed a mechanism with two possible pathways. The first pathway involves a Ni^0^/Ni^II^/Ni^III^/Ni^I^ catalytic sequence. Initially, the oxidative addition of alkenyl bromide to Ni(0) generates a Ni­(II) species (**II**). Simultaneously, at the anode, the in situ-generated nucleophilic Et_3_N-HX causes ring-opening of the aziridine, producing the β-halo-sulfonamide intermediate (**III**). This intermediate undergoes a single-electron transfer or halogen atom abstraction process, forming a corresponding benzyl radical (**IV**), which then forms a Ni­(III) intermediate (**V**). Following this, reductive elimination releases the expected cross-coupling product, leaving a Ni­(I) intermediate. This Ni­(I) intermediate is reduced at the cathode to regenerate Ni(0), which continues the cycle. The second pathway involves a Ni^I^/Ni^II^/Ni^III^ catalytic sequence, where under reducing conditions, Ni­(II)­Br_2_ can be reduced to Ni­(I)­Br at the cathode, and the oxidative addition of alkenyl bromide directly generates the key Ni­(II) complex (**II**).

**Scheme 10:**
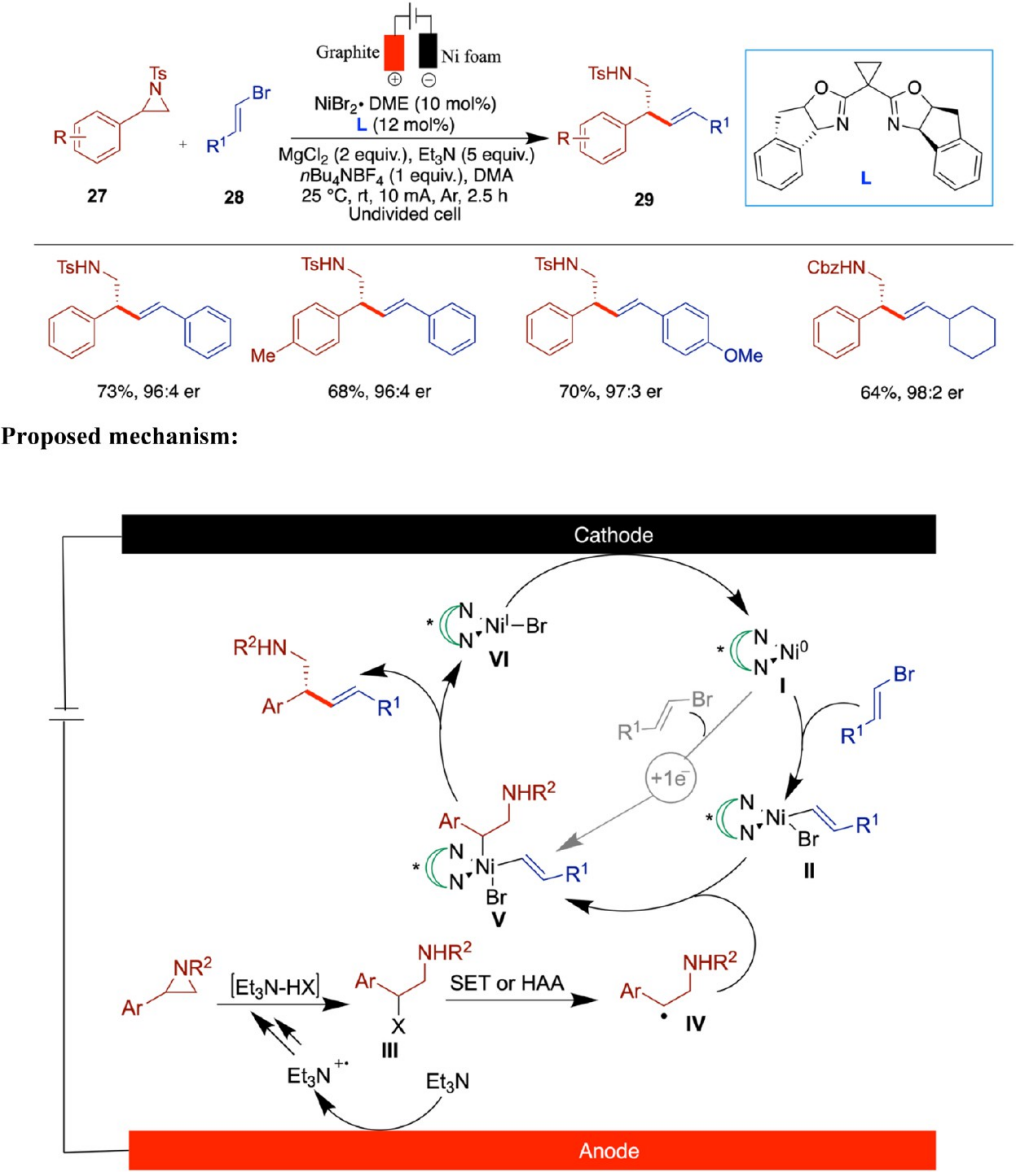
Electrochemical Nickel-Catalyzed Enantioselective Reductive Cross-Coupling of Aziridines with Alkenyl Bromides

Very recently, the Mei group reported an enantioselective electrochemical reductive cross-coupling (eRCC) of benzyl chlorides with aryl halides for the synthesis of 1,1-diaryl compounds using bis-imidazolidine ligand (**L8**) with good to excellent enantioselectivity (Scheme 11).^47^ This method does not require a sacrificial anode, which enhances its scalability. A wide range of aryl halides, including heteroaryl halides, can be used, and the authors further extended the method to late-stage drug diversification. Control studies using the conventional reductant manganese resulted in little to no reaction compared to the electrochemical conditions. The authors proposed a mechanism for the Ni-catalyzed enantioselective electrochemical reductive cross-coupling (eRCCs), in which the catalytic cycle is initiated by the cathodic reduction of the Ni­(II) precatalyst, generating a Ni­(I) species (**A**). Subsequently, the oxidative addition of an aryl halide to this Ni­(I) species yields an aryl-Ni­(III) species (**B**), which undergoes further cathodic reduction to generate an aryl-Ni­(I) species (**C**). The benzyl chloride is then activated by species C, leading to the formation of a benzyl radical intermediate via single-electron transfer (SET) and the concurrent generation of an aryl-Ni­(II) species (**D**). The Ni­(III) intermediate (**E**) is formed upon trapping of the benzyl radical by intermediate **D**, and subsequent reductive elimination furnishes the cross-coupled product while regenerating the Ni­(I) species (**A**), thereby completing the catalytic cycle. Additionally, the anodic oxidation of triethylamine facilitates the overall reaction.

**Scheme 11:**
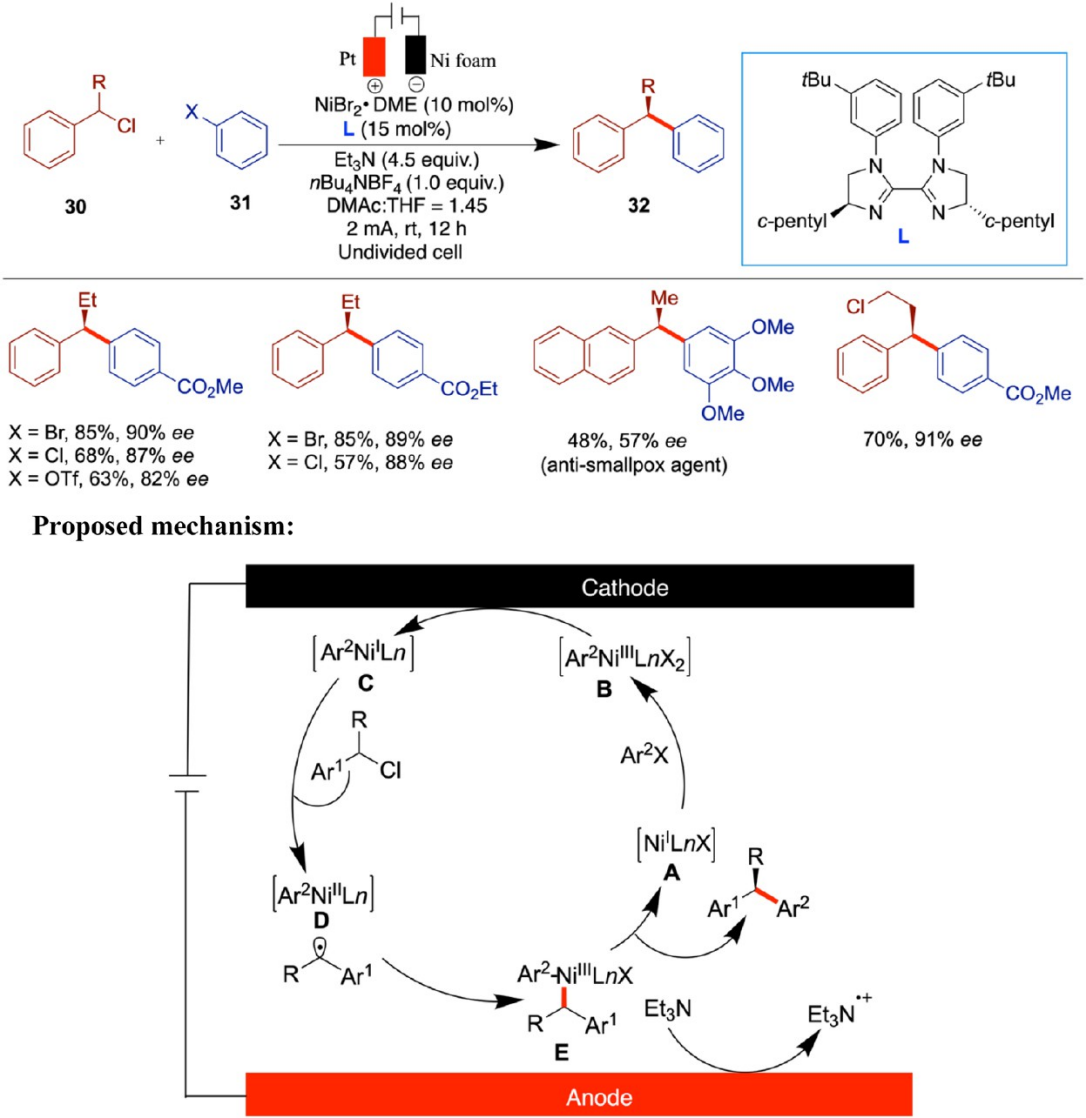
Electrochemical Cross-Coupling of Benzyl Halides with Aryl Halides

## Cross-Electrophile Coupling

Another critical area of advancement in nickel catalysis is cross-electrophilic coupling, a method that enables the direct coupling of two electrophiles, typically an organic halide and a metal catalyst to form new bonds.^48^ Unlike traditional cross-coupling, which usually involves the coupling of an electrophile with a nucleophile, cross-electrophile coupling simplifies the synthesis of complex molecules by eliminating the need for prefunctionalized reagents.^49^ This approach is particularly valuable in constructing molecular architectures with high efficiency and selectivity, making it an important tool in organic synthesis.^50^

Recent years have seen substantial progress in nickel-catalyzed cross-electrophile coupling. The development of novel catalytic systems and reaction conditions has expanded the scope of substrates that can be used in these reactions, allowing for the efficient synthesis of complex molecules under mild and sustainable conditions. Nickel-catalyzed reactions have shown higher functional group tolerance and can operate under milder conditions compared with those catalyzed by other metals, such as palladium. This versatility makes nickel an attractive choice for synthetic chemists who aim to develop novel methodologies.

In 2020, Mei and colleagues reported on the nickel-catalyzed reductive relay cross-coupling of alkyl halides with aryl halides in an undivided cell (Scheme 12).^51^ When they reacted alkyl bromides with aryl halides, an electrochemical reductive relay cross-coupling occurred, leveraging the chain-walking ability of an alkyl-nickel species. The authors demonstrated the versatile reactivity of both aryl and alkyl halides including three or four carbon chain lengths, which resulted in moderate to good yields. However, electron-rich aryl chlorides proved to be ineffective under the reported conditions.

**Scheme 12:**
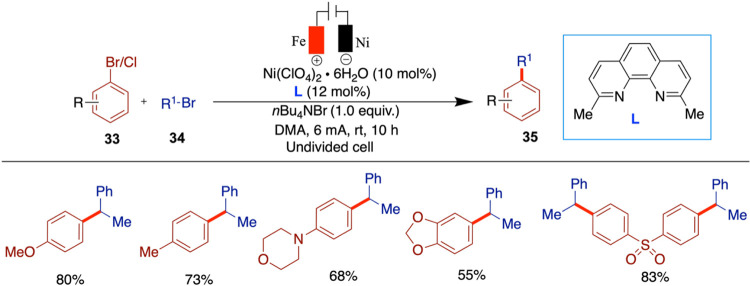
Nickel-Catalyzed Reductive Relay Cross-Coupling of Alkyl Halides with Aryl Halides

In 2017, Hansen et al. reported an electrochemical nickel-catalyzed cross-coupling of aryl halides with alkyl halides using DMA as the solvent, a sacrificial zinc anode, and a reticulated vitreous carbon (RVC) cathode. Unlike traditional methods that rely on stoichiometric zinc powder as a reductant, this approach employs electrochemical reduction to generate catalytically active low-valent nickel species, offering a more sustainable and controlled alternative (Scheme 13).^52^ In this study, the use of various ligands is necessary to achieve quantitative yields, depending on the specific aryl and alkyl halides used. The authors aimed to demonstrate that electrochemical conditions can be as efficient as or superior to conventional metal-powder reductants. In several cases, electrochemical methods delivered yields comparable to those obtained using traditional reductants, such as zinc powder. For example, the cross-coupling of cyclopentyl bromide with 4-trifluoromethylphenyl bromide gave an 82% yield under electrochemical conditions compared to 81% under zinc-mediated conditions. This demonstrates the method’s effectiveness and highlights instances where electrochemistry can even outperform conventional setups in selectivity and efficiency. Notably, in certain examples, electrochemical conditions gave significantly higher yields almost double those of the corresponding metal-reductant-based reactions. Despite this progress, the substrate scope remains somewhat constrained, as some alkyl halides still lead to moderate or low yields, suggesting room for further optimization. The authors proposed a mechanism based on the Weix report, which follows the conventional metal-reductant-based pathway.^53^ In this mechanism, the Ni­(II) complex undergoes cathodic reduction to generate a Ni(0) species (**A**). This Ni(0) species then undergoes oxidative addition with the aryl halide to form a Ni­(II) aryl complex (**B**), which subsequently couples with an alkyl radical to yield a Ni­(III) intermediate (**C**). Finally, reductive elimination from this Ni­(III) species furnishes the expected cross-coupled product.

**Scheme 13:**
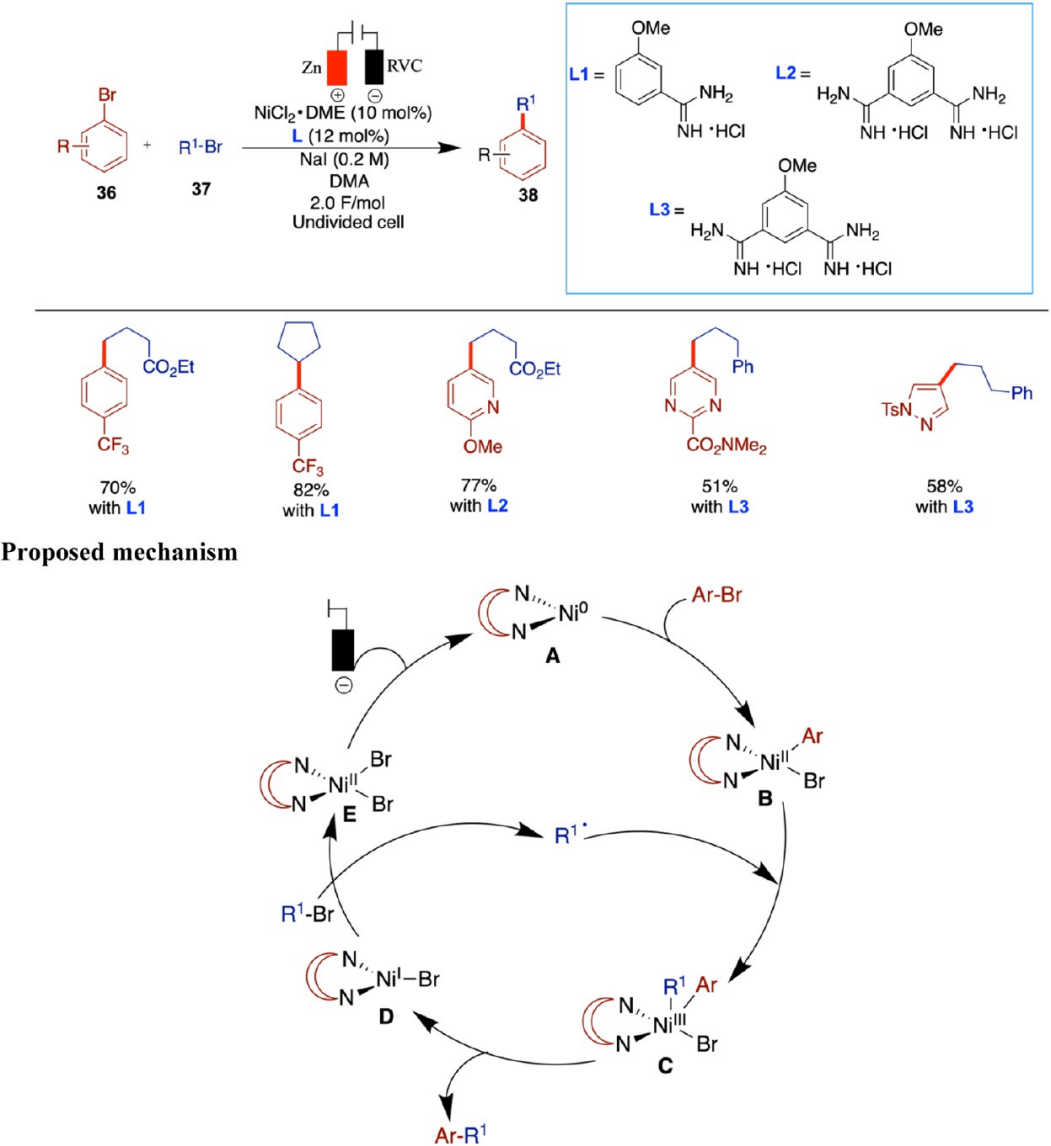
Nickel-Catalyzed Cross-Coupling of Aryl Halides with Alkyl Halides

More recently, in 2022, Weix et al. presented a scalable zinc-free reductive cross-electrophile coupling driven by electrochemistry without sacrificial electrode (Scheme 14).^54^ In this work, the authors removed the reliance on toxic polar solvents and used acetonitrile as a nontoxic user-friendly solvent to realize the cross-coupling. They identified a combination of two ligands, 4,4′-di-*tert*-butyl-2,2′-bipyridine and 4,4′,4″-tri-*tert*-butyl-2,2′:6,2″-terpyridine, which enhance the cross-coupling. In contrast, using a single ligand decreases the yield by promoting dimerization. While this method demonstrates broad applicability, certain limitations were noted. Sterically hindered substrates, such as neopentyl bromide and methyl 2-bromobenzoate led to lower yields. Moreover, the reactivity of electron-rich aryl bromides (e.g., 4-bromoanisole) was diminished, requiring an adjustment of the ligand ratio to enhance yields. These findings highlight that careful tuning of the ligand ratios is essential to minimize side products and to address challenges posed by less reactive or sterically demanding coupling partners. In the mechanistic proposal involving alkyl bromides and Ni­(I) species, the formation of alkyl radicals occurs via a single-electron-transfer (SET) process. The authors proposed the mechanism based on related precedents reported by Umehara^55^ and Vannucci.^56^ In this step, the Ni­(I) species acts as a reductant and donates an electron to the alkyl bromide, resulting in homolytic cleavage of the C–Br bond. This generates an alkyl radical and a bromide anion while oxidizing Ni­(I) to Ni­(II). Thus, in this transformation, the alkyl bromide is reduced, and the nickel center is oxidized from Ni­(I) to Ni­(II), consistent with a halogen atom abstraction.

**Scheme 14:**
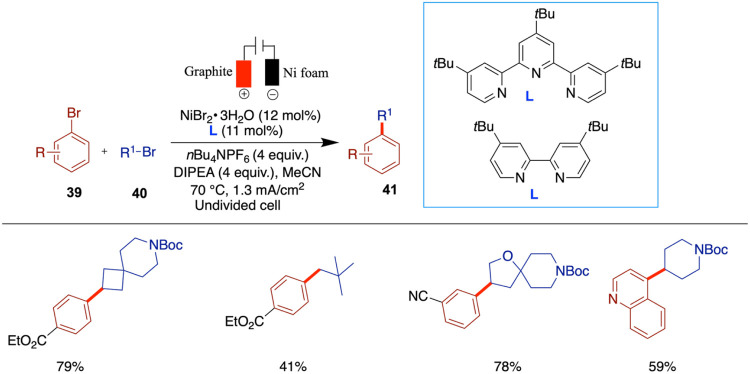
Zinc-Free Reductive Cross-Electrophile Coupling

Li and co-workers reported nickel-catalyzed electrochemical cross-coupling of alcohols with aryl halides (Scheme 15).^57^ The key to the success of this approach lies in the formation of a key intermediate, the alkoxy triphenyl phosphonium ion, at the anode. The cathodic reduction then facilitates the expected cross-coupling to construct C­(sp^3^)–C­(sp^3^) bonds. The commercial availability of both alcohols and aryl halides highlights the potential of this method in synthesis. Through this paired electrolysis, catalyzed by nickel, the authors explored a variety of substrates, including primary and secondary alkyl alcohols as well as π-activated alcohols, and found that less sterically hindered alcohols exhibited higher reactivity, while tertiary alcohols were not accommodated. The method was further demonstrated on pharmaceuticals containing hydroxy groups including but not limited to ospemifene, bucetin, simvastatin, and cortisone.

**Scheme 15:**
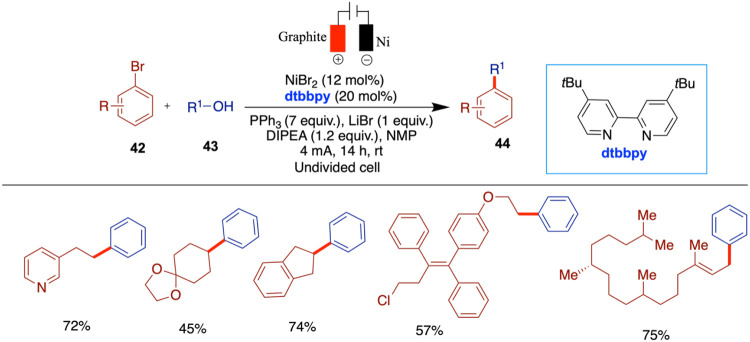
Electrochemical Cross-Coupling of Alcohols with Aryl Halides

The electrochemical nickel-catalyzed C­(sp^3^)–C­(sp^3^) cross-coupling of alkyl halides with alkyl tosylates was reported by Cantillo and co-workers in 2023 (Scheme 16).^58^ Secondary bromides and tosylates could also be used in this strategy. The achievement of high selectivity was facilitated by using tosylates in conjunction with sodium bromide as the supporting electrolyte. This combination enabled the gradual generation of small quantities of bromide through substitution, thereby ensuring that one of the reaction partners remained in excess throughout much of the nickel-catalyzed electroreductive process. The authors screened various alkyl tosylates with alkyl halides and observed good conversions, yielding cross-coupled products in moderate to good yields. Interestingly, secondary tosylates resulted in very low conversions, and some did not perform well under the optimized conditions.

**Scheme 16:**
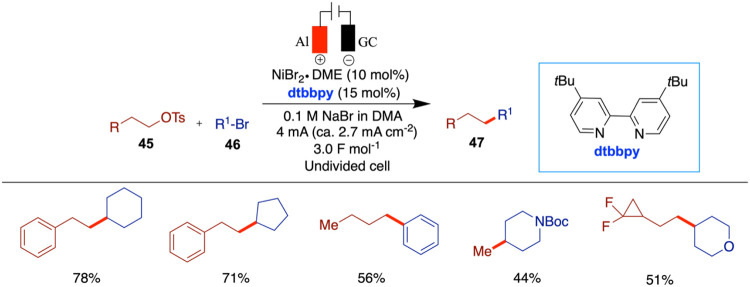
Nickel-Catalyzed C­(sp^3^)–C­(sp^3^) Cross-Coupling

Baran et al. have reported a chemoselective and scalable nickel electrocatalytic O-arylation of alcohols to produce aryl-alkyl ethers (Scheme 17).^59^ While the mechanism of this arylation of alcohols is similar to that of photochemical methods^60,61^ and conventional nickel-catalyzed cross-coupling protocols,^62^ the electrochemical approach offers an oxidant-free alternative with improved functional group tolerance. The inclusion of a tertiary amine moiety in this approach enhances the applicability of the electrochemical synthesis for ether formation. A wide variety of aryl halides and alcohols can be employed in this electrochemical etherification, affording products in moderate to good yields. The method exhibits broad functional group tolerance, accommodating both reductively and oxidatively sensitive motifs such as halogens (Br, Cl, and F), esters, ketones, aldehydes, amines, carbamates, boronates, and various heterocycles. Primary and secondary alcohols were effective coupling partners, while tertiary alcohols proved to be inefficient. Notably, complex polyfunctionalized fragments, including nucleosides, were also compatible, although dietherification was minimal when using substrates like 1,4-dibromobenzene due to low reactivity with low-valent nickel species. The study also compares this method with conventional approaches, highlighting its advantages and overall attractiveness.

**Scheme 17:**
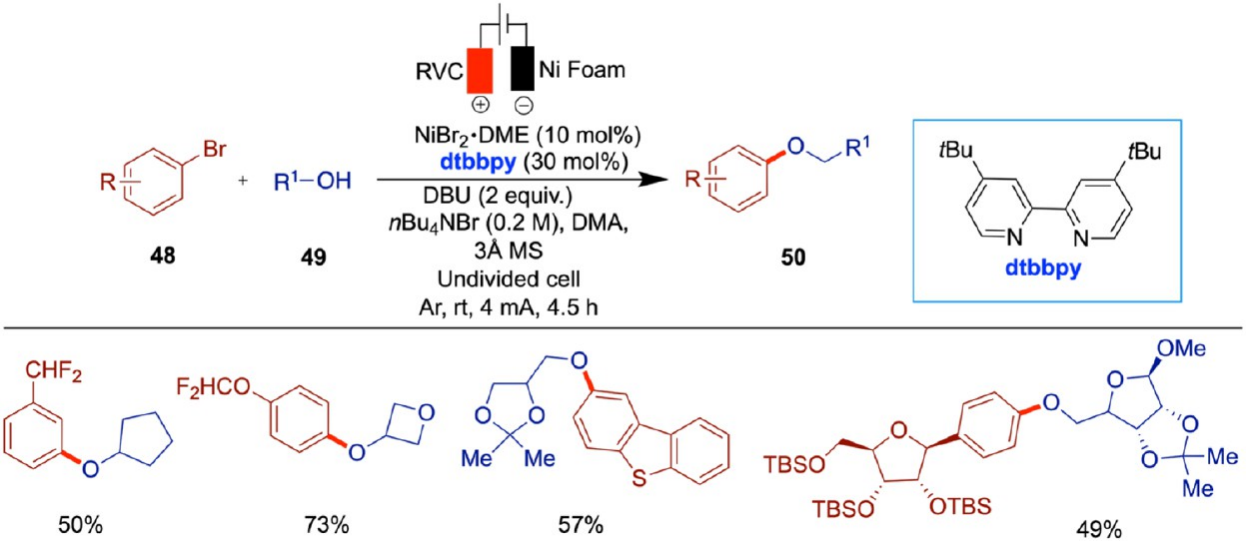
Nickel-Electrocatalytic *O*-Arylation of Alcohols

The literature contains very few examples of anodic oxidation of nucleophiles for C–C bond formation without the use of sacrificial stoichiometric electron donors. However, in 2021, Liu and co-workers reported a nickel-catalyzed C­(sp^3^)–C­(sp^2^) cross-coupling reaction using benzyl trifluoroborate as the nucleophile and bench-stable organic halides or β-bromostyrene as the electrophile in an undivided cell, employing a NiCl_2_·glyme and polypyridine catalytic system (Scheme 18).^63^ As a control experiment, when 4-bromophenol was used as the electrophile instead of 4-methoxybromobenzene, no cross-coupling product was observed. This outcome is attributed to the preferential oxidation of the substrate itself, which occurs at a potential less positive than that required for the borate nucleophile. This observation underscores the importance of protecting oxidation-sensitive functional groups under the applied electrochemical conditions. The methodology shows potential for late-stage functionalization of commercial pharmaceuticals and amino acid modification. The proposed mechanism involves the characteristic cathodic reduction of the Ni­(II)­L complex (**A**) to Ni­(I)­L (**B**), which undergoes oxidative addition with aryl halides to form a Ni­(III) intermediate (**C**). This Ni­(III) species is then reduced at the cathode to Ni­(II) (**D**), which combines with the alkyl radical (**E**) generated via anodic oxidation of benzyl borates at the anode through a single-electron transfer (SET). The resulting Ni­(III) intermediate (**F**) undergoes reductive elimination to give the final product.

**Scheme 18:**
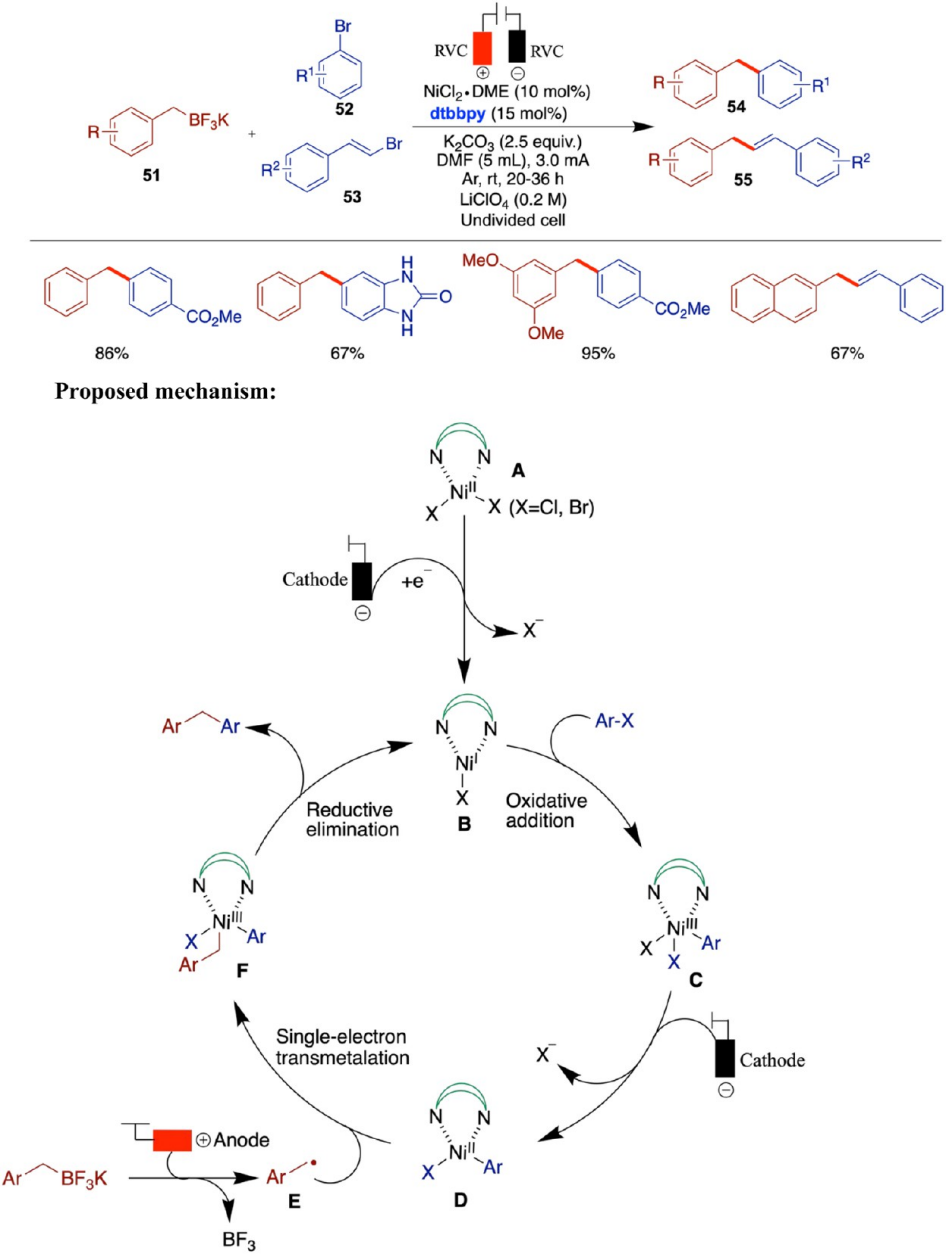
Nickel-Catalyzed C­(sp^3^)–C­(sp^2^) Cross-Coupling Reaction Using Benzyl Trifluoroborate

In 2020, Rueping et al. reported electrochemical nickel-catalyzed cross-electrophile coupling of readily available alkyl and aryl halides and olefin hydroarylation enabled by electrochemical reduction, which provided access to 1,1-diarylalkane derivatives (Scheme 19).^64^ This method showed compatibility with wide functional group tolerance and nonreactive secondary alkyl halides could also be incorporated. From the mechanistic studies, the authors proposed that the nickel hydride formed in the electroreductive chain-walking arylation is the crucial step for this hydroarylation reaction. The possibility of a chain-walking pathway competing with the formation of allylbenzene from alkyl halides was investigated by the authors through time-course experiments. This analysis confirmed that allylbenzene was not formed, indicating that chain-walking proceeds rapidly and continues until the more stable benzylic position is reached. The rate-determining step was identified as continuous generation of the internal olefin.

**Scheme 19:**
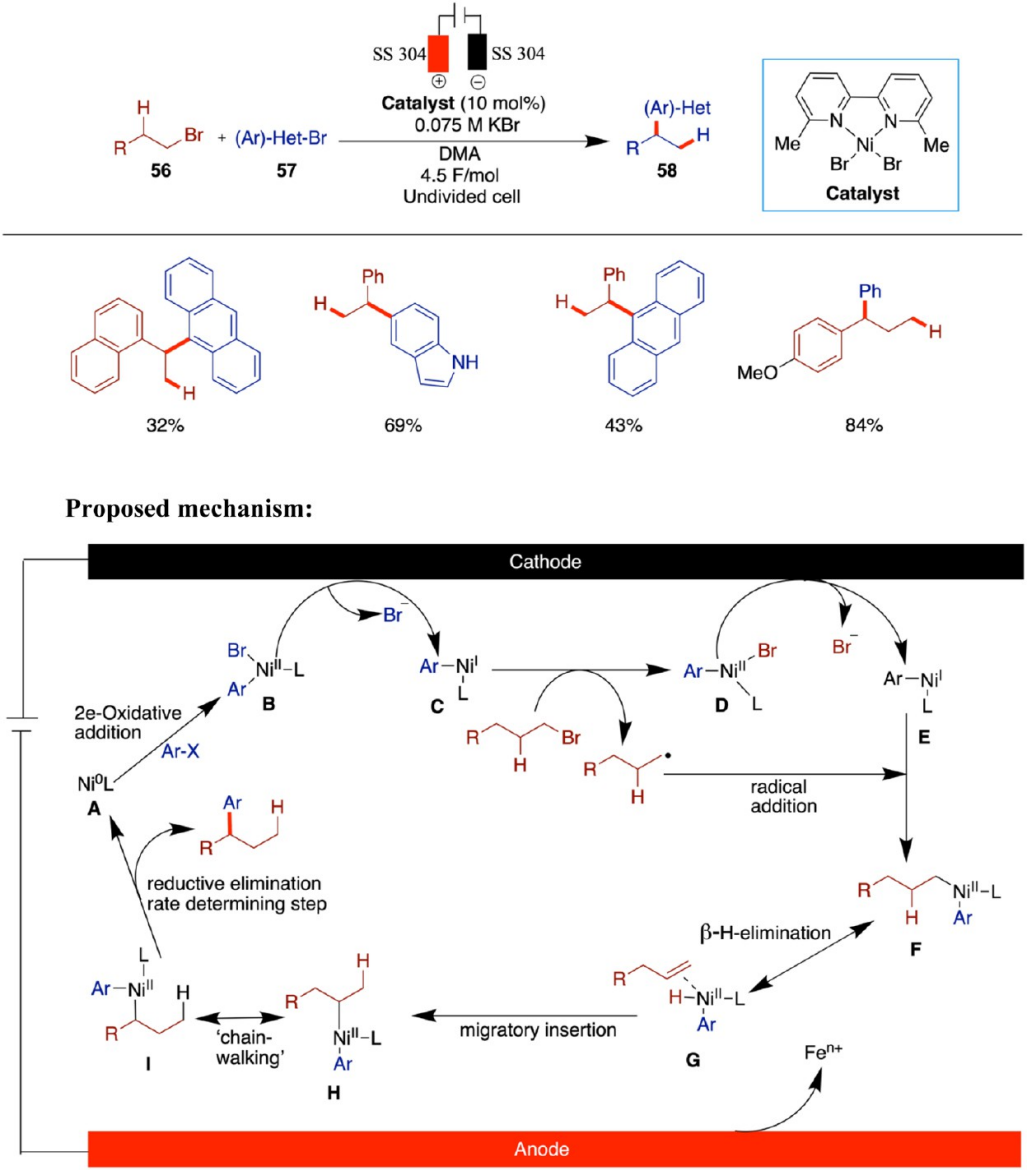
Cross-Electrophile Coupling of Alkyl and Aryl Halides

Based on their findings, the authors proposed a mechanism that begins with the reductive generation of a Ni(0) species at the cathode. This species undergoes oxidative addition with the aryl bromide via a two-electron process, followed by one-electron reduction to afford intermediate **C**. This is followed by a single-electron-transfer (SET) oxidative addition with the alkyl bromide, generating an alkyl radical and intermediate **D**. A subsequent one-electron cathodic reduction produces intermediate **E**. The alkyl radical then adds to the Ni­(I) species, yielding the Ni­(II) intermediate **F**. From this point, chain-walking occurs via β-hydride elimination to form Ni­(II)-hydride species **G**, which undergoes reinsertion to generate intermediate **H**. Final reductive elimination from **I** furnishes the desired product.

Unlike previous reports of reductive cross-coupling for C­(sp^3^)–C­(sp^3^) bond formation, which predominantly used alkyl halides, concerns over the cost and toxicity of these reagents have motivated the search for alternative coupling partners. Recently, Noel and colleagues reported a nickel-catalyzed electroreductive cross-electrophile coupling of alkyl amine-derived radical precursors with aryl iodides (Scheme 20).^65^ The amine-derived alkyl radical precursors, known as Katritzky salts, proved highly effective in this electrochemical transformation, affording a variety of products with diverse substituents in good yields. The scope of amines was largely limited to cyclic and a few acyclic amines. In addition, reduced reactivity was observed with some aryl halides, such as phenyl iodide. Aryl iodides bearing electron-withdrawing groups, however, gave good yields. When Katritzky salts derived from primary amines were tested, only trace amounts of the desired products were obtained.

**Scheme 20:**
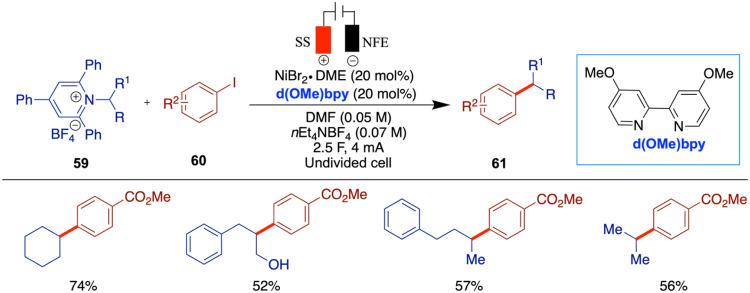
Electro-Reductive Cross-Electrophile Deaminative Coupling with Aryl Iodides

Alkylpyridinium salts have a more favorable reduction potential compared with other alkyl electrophiles commonly used in electrochemical reductive coupling. Due to their ease of reduction, these salts can be preferentially reduced over Ni-intermediates. This preferential reduction can lead to off-cycle byproducts through mechanisms such as trapping of the dihydropyridyl radical, dimerization, or reduction of alkyl halides. Almost at a similar time, Watson, Sevov, and Kalyani et al. all reported electroreductive coupling of alkylpyridinium salts and aryl halides (Scheme 21).^66^ As Noel and colleagues also used a variety of alkyl electrophiles and various amines for the cross-coupling. It is important to note that the authors systematically optimized key reaction parameters, including the nature of aryl halides (electron-rich vs electron-deficient), amine coupling partners, ligands, base, and solvent conditions, to achieve high yields and selectivity.

**Scheme 21:**
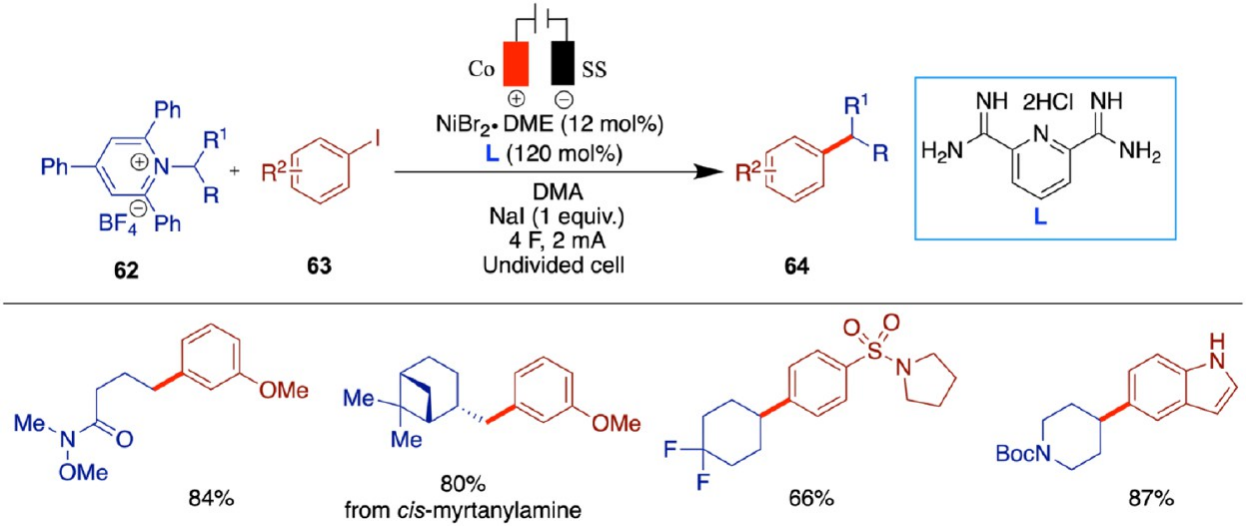
Electroreductive Coupling of Alkylpyridinium Salts and Aryl Halides

Liu and co-workers reported the electro/Ni dual-catalyzed redox-neutral decarboxylative C­(sp^3^)–C­(sp^2^) cross-coupling reactions of pristine carboxylates with aryl bromides (Scheme 22).^67^ This method features an innovative paired electrolysis strategy that eliminates the need for sacrificial oxidants or reductants by employing nonsacrificial carbon-based electrodes, thereby improving both the sustainability and practicality of the process. The method utilizes carboxylates, which are nontoxic, stable, cost-effective, and widely accessible, making them excellent nucleophiles for C–C cross-coupling reactions. In this reaction, the cathode facilitates the formation of a Ni^II^(Ar)­(Br) intermediate by activating the Ar–Br bond through a Ni^I^-bipyridine catalyst, followed by reduction. Concurrently, at the anode, diverse carboxylates such as amino acids, benzyl carboxylic acids, and 2-phenoxy propionic acid undergo oxidative decarboxylation, generating carbon-based free radicals. These radicals subsequently interact with the Ni^II^(Ar)­(Br) intermediate, leading to the production of C­(sp^3^)–C­(sp^2^) cross-coupling products. The authors further investigated the reaction mechanism using cyclic voltammetry and DFT computational studies, elucidating the correlation between the electrochemical properties of the carboxylates and reaction selectivity. The authors demonstrated the successful adaptation of this electrosynthetic method to both flow synthesis and the synthesis of value-added molecules. This advancement broadens the chemical space accessible via paired electrochemical C­(sp^3^)–C­(sp^2^) cross-coupling and highlights the method’s potential for scalable applications, supported by the ready availability of carboxylate nucleophiles and the flexibility of electrochemical flow technology.

**Scheme 22:**
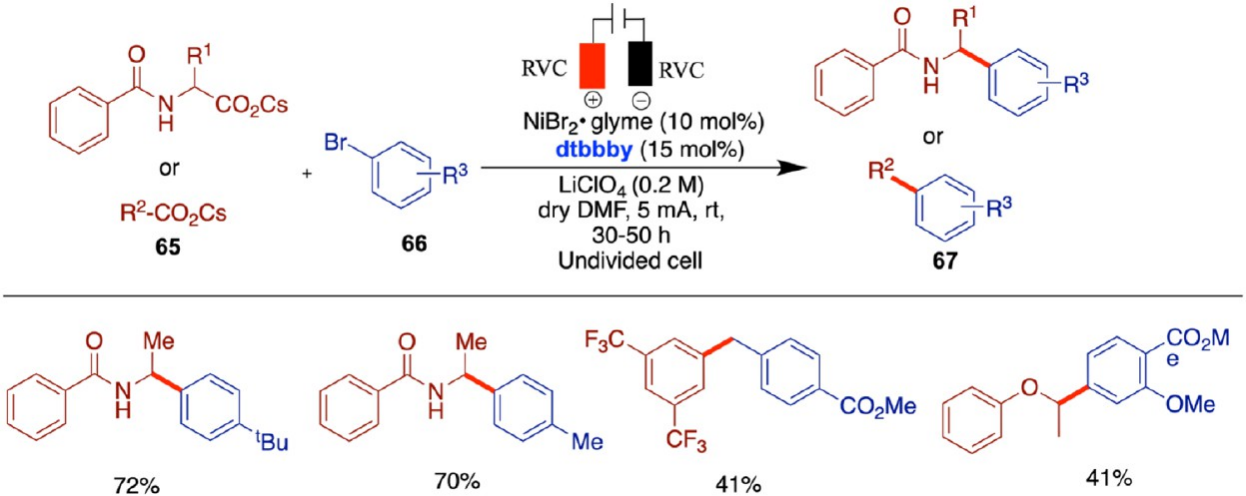
Decarboxylative C­(sp^3^)–C­(sp^2^) Cross-Coupling of Carboxylates with Aryl Bromides

### Nickel-Catalyzed Hydrogenation

Olefin hydrogenation is a pivotal process in organic synthesis, offering vast potential for transformation to synthesize important molecules. Electrocatalytic hydrogenation mediated by transition metals has emerged as a promising avenue to supplant hazardous hydrogen gas with electrons and protons. Nevertheless, this method encounters significant hurdles, notably the rapid hydrogen evolution reaction (HER) of metal-hydride species, overshadowing the alkene hydrogenation step, and the facile deposition of the metal catalyst on the electrode, impeding the reaction progress. A recent paper by Fu and co-workers, uncovered an economical and efficient approach to enhance selectivity for hydrogenation reactivity over the prevailing HER (Scheme 23).^68^ Leveraging a cost-effective and stable nickel salt catalyst (NiCl_2_·6H_2_O), the authors demonstrate remarkable substrate versatility, with broad compatibility across diverse olefins, including mono-, di-, and trisubstituted alkenes, aryl alkenes, and unactivated α-olefins. The method also tolerates functionalized substrates such as norbornene, cyclopentene, and naturally occurring enones like progesterone and testosterone, highlighting its notable chemoselectivity and potential for late-stage functionalization. Moreover, they have demonstrated the hydrodebromination of alkyl and aryl bromides within the same reaction framework, employing a distinct ligand [4,4-di-*tert*-butyl-2,2-dipyridyl (dtbbpy)], and achieving high chemoselectivity between hydrogenation and hydrodebromination through ligand modulation. In the proposed mechanism, the initial formation of a [Ni­(TEOA)_2_]^2+^ complex (**A**) is followed by partial ligand exchange with the alkene substrate to give a Ni­(II) species (**B**). Cathodic reduction generates Ni(0) intermediate **C**, which can then proceed via two possible pathways to form the alkyl–Ni­(II) intermediate **E**: either through a nickelacyclopropane species (**D**) or via oxidative addition followed by migratory insertion. Final proto-demetalation releases the product and regenerates the Ni­(II) species.

**Scheme 23:**
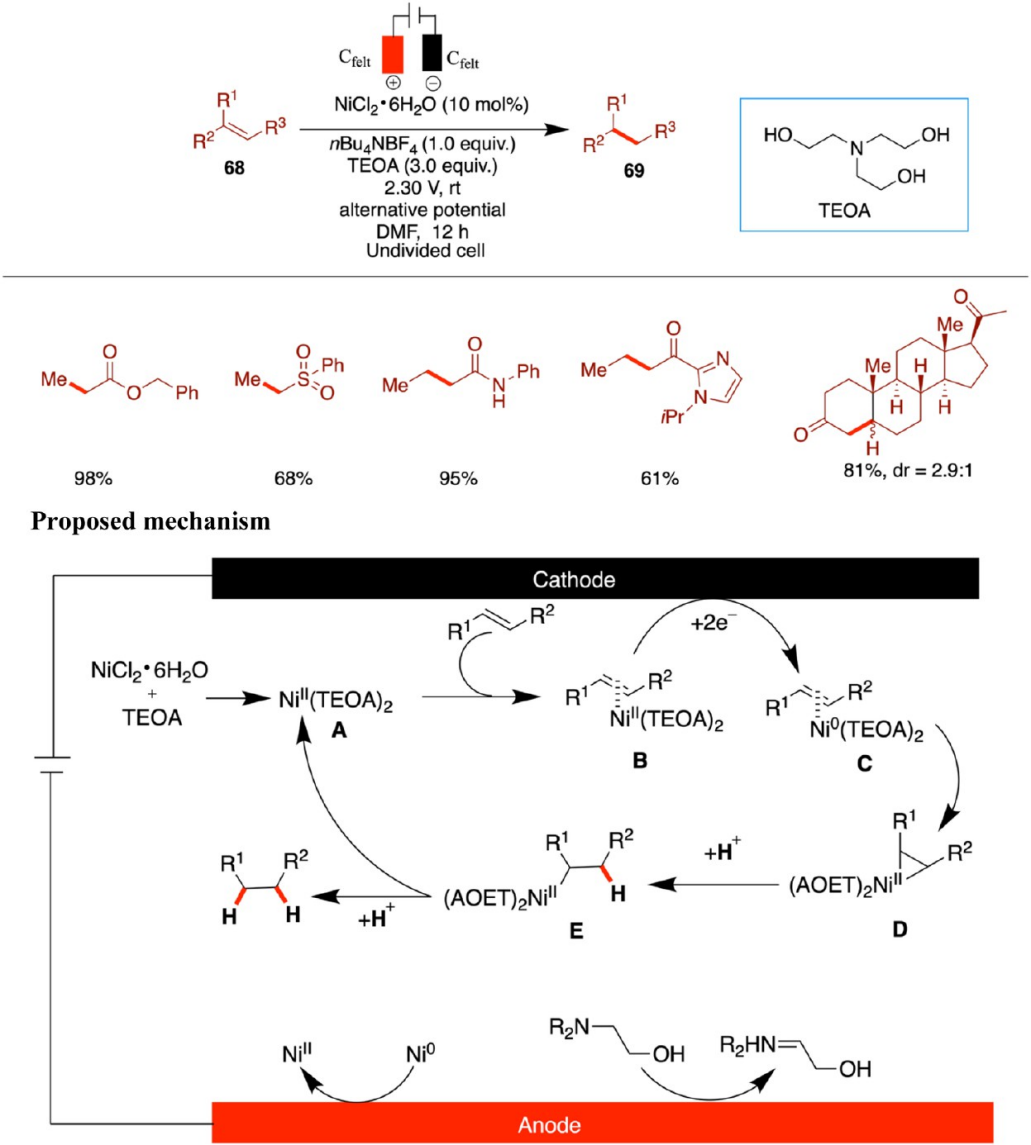
Nickel-Catalyzed Electrochemical Hydrogenation

Zeng and Xu reported electrochemical nickel-catalyzed reductive decarboxylative coupling of *N*-hydroxyphthalimide (NHP) esters with quinoxalinones for the synthesis of 3-alkylated quinoxalinones (Scheme 24).^69^ This method, which employed NiCl_2_ as a catalyst, offers broad substrate compatibility, accommodating primary, secondary, and tertiary aliphatic carboxylic acids, as well as amino acid-derived esters. The reaction proceeds under mild conditions and gave high yields of structurally diverse 3-alkylated quinoxalinones, making it an attractive alternative to traditional chemical oxidant-based methods. This reaction combines the advantages of electrochemistry and nickel catalysis, underscoring its potential for sustainable and scalable organic synthesis. The authors proposed a plausible mechanism which begins with the reduction of Ni­(II) to Ni­(I) at the cathode, followed by a single-electron transfer from Ni­(I) to the NHP ester, generating a Ni­(II) species and an alkyl radical. This radical then adds to the quinoxalinone substrate, forming an intermediate that undergoes further oxidation and proton loss to yield the 3-alkylated quinoxalinone product. The role of NEt_3_ as an oxidizable amine at the anode is crucial in maintaining the catalytic cycle. Control experiments using radical inhibitors such as TEMPO [(2,2,6,6-Tetramethylpiperidin-1-yl)­oxyl] and BHT [Butylated hydroxytoluene] confirmed the involvement of radical intermediates, as evidenced by the significant reduction or complete inhibition of the product formation in their presence. Cyclic voltammetry studies further supported the proposed mechanism by showing distinct reduction peaks corresponding to the intermediates involved in the catalytic cycle.

**Scheme 24:**
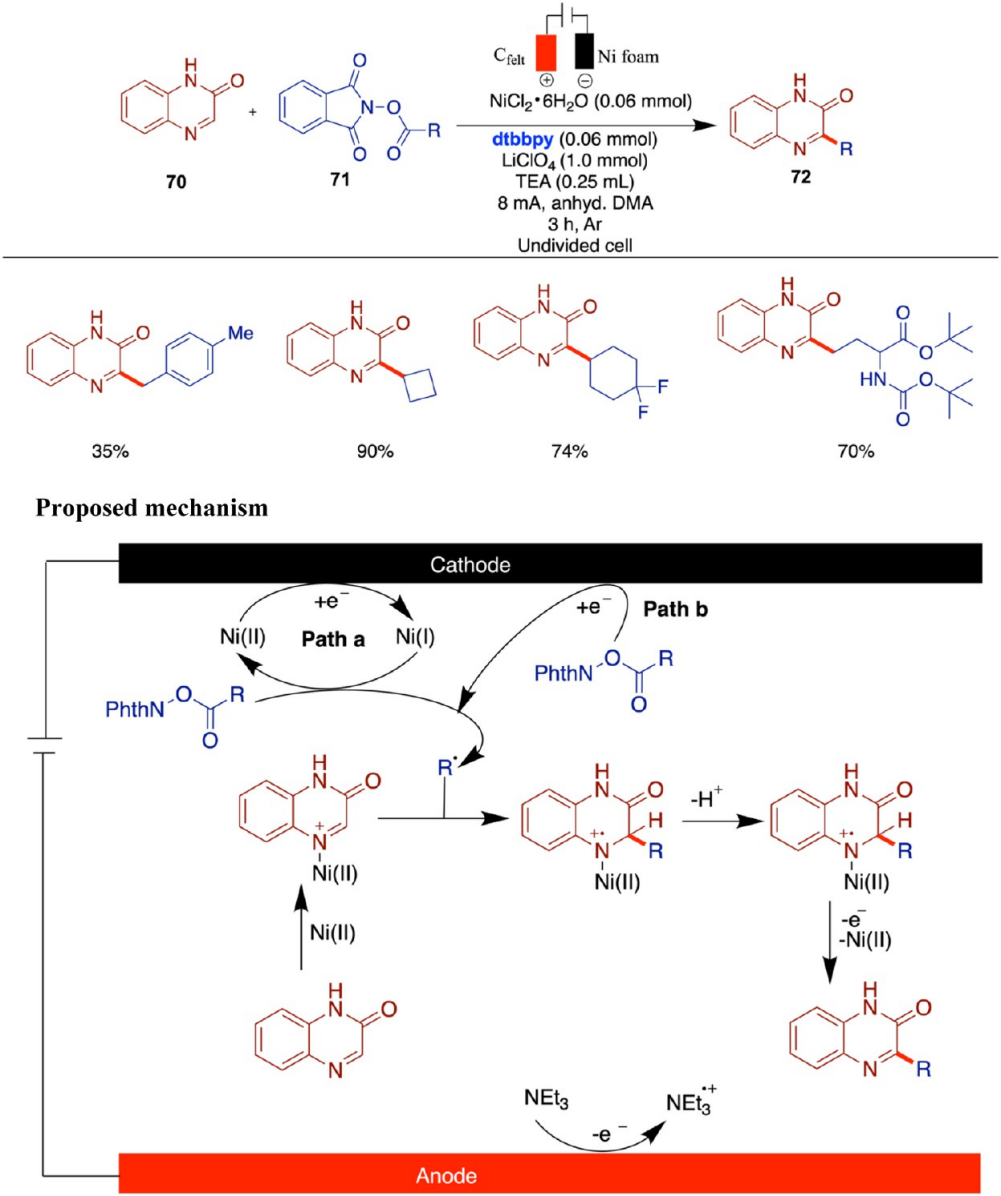
Decarboxylative Coupling of *N*-Hydroxyphthalimide (NHP) Esters with Quinoxalinones

The C­(sp^3^)–C­(sp^3^) bond holds ubiquitous significance in natural products, drugs, and various functional organic molecules. Consequently, the synthesis of these bonds stands as a highly coveted and challenging pursuit within the realms of synthetic organic chemistry, drug discovery, and associated fields. Wang and Qiu et al. reported an innovative approach to the construction of C­(sp^3^)–C­(sp^3^) bonds through electrochemically driven NiH-catalyzed reductive coupling of alkyl halides and alkyl alkenes.^70^ A distinctive aspect of this method is the dual role of alkyl halides, which act as both coupling substrates and hydrogen sources to generate NiH species under electrochemical conditions. The versatility of this reaction is enhanced by the incorporation of an intramolecular coordinating group into the substrate, allowing for facile adjustment of the product to yield the desired branched products. Noteworthy features include mild reaction conditions and broad substrate compatibility, as demonstrated across more than 70 examples. The method tolerates a wide range of unactivated alkenes and aryl motifs, including phenol- and benzene-derived alkenes, heterocycles (indole, pyridine, and furan), and sensitive functional groups such as boronic esters, free hydroxyl and carboxylic acids, and amines. The transformation shows high selectivity for terminal alkenes over internal and cyclic ones and for alkyl bromides over chlorides. In addition, a variety of cyclic and linear alkyl halides, including those bearing N-Boc, carbonyl, and spirocyclic substituents, were compatible. Notably, the method enables late-stage functionalization of complex molecules, such as steroids, NSAIDs, and sugars, highlighting its utility in medicinal chemistry. Mechanistic insights elucidated the electrochemically driven coupling process, highlighting the activation of sp^3^-carbon–halogen bonds through single-electron transfer (SET) by the nickel catalyst in its low valence state, which is generated via cathodic reduction. The key role of NiH species derived from alkyl halides in this transformation is also underscored (Scheme 25). When they used 8-aminoquinoline as an internal directing group, the branched products were obtained in good yields. The proposed mechanism begins with the cathodic reduction of a Ni­(II) complex to generate Ni(0), which then undergoes single-electron transfer (SET) with an alkyl halide, forming a Ni­(I)–Br species (**A**) and an alkyl radical. This Ni­(I) complex undergoes oxidative addition with the alkyl radical to afford a Ni­(II) species (**B**). A subsequent β-hydride elimination leads to the formation of a Ni–H intermediate (**c**), which is intercepted by an unactivated alkene to form species **D**. This intermediate then undergoes another SET oxidation to generate species **E**. Finally, reductive elimination from **E** delivers the desired linear product.

**Scheme 25:**
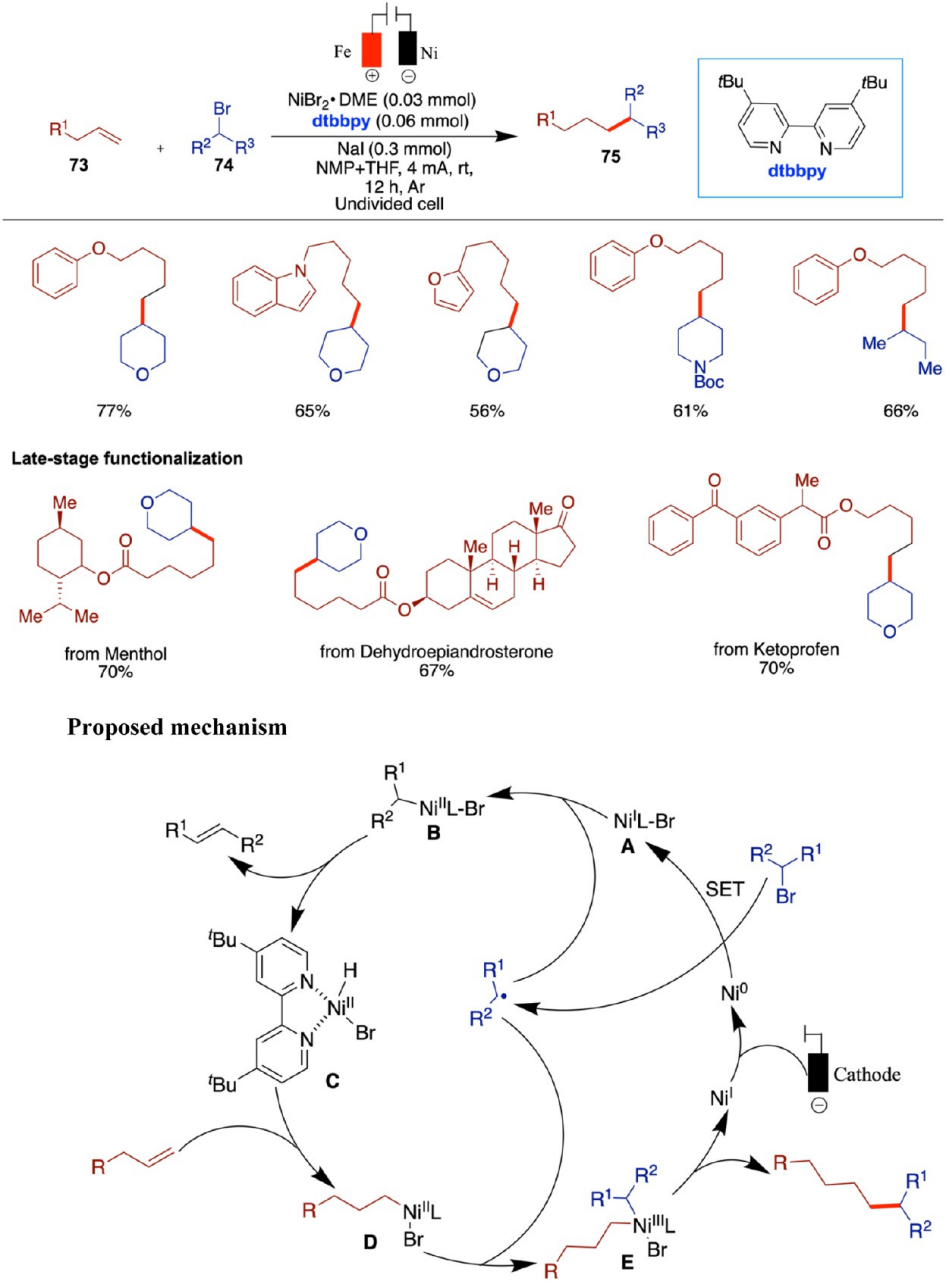
NiH-Catalyzed Reductive Coupling of Alkyl Halides and Alkyl Alkenes

The development of a nickel-catalyzed electrochemical method for synthesizing (hetero)­aryl trifluoromethyl selenides represents a significant advancement in the field of organofluorine chemistry. Durandetti and Krykun reported a novel and efficient method for synthesizing (hetero)­aryl trifluoromethyl selenides via nickel-catalyzed electrochemical processes (Scheme 26).^71^ This approach employs stable and inexpensive NiBr_2_bpy as a catalyst, replacing the more labile Ni­(COD)_2_, and utilizes [NMe_4_]­[SeCF_3_] as a shelf-stable source of the SeCF_3_ fragment. This innovation addresses a key challenge in the synthesis of SeCF_3_-containing compounds, providing a reliable and practical alternative to existing methods. The reaction proceeds under mild conditions and exhibits a broad substrate scope, tolerating functional groups such as esters, halogens (Cl, Br, F), and boronic esters, as well as heteroaryl iodides like indoles and pyridines. However, limitations were observed with sterically hindered ortho-substituted aryl iodides and 4-phenoxy aryl iodide, the latter giving a notably poor yield under the standard conditions. Cyclic voltammetry studies provide insights into the reaction mechanism, revealing the electrochemical behavior of the catalytic system. The catalytic cycle begins with the formation of a Ni­(II)–[SeCF_3_] (**A**) complex via anion exchange between NiBr_2_–bipyridine and [NMe_4_]­[SeCF_3_]. Upon electrochemical reduction, a Ni(0)–[SeCF_3_]–bipyridine species (**B**) is generated. This Ni(0) complex undergoes oxidative addition with aryl iodide to form a Ni­(II)–aryl intermediate (**C**). Reductive elimination from this species affords the aryl–SeCF_3_ product and regenerates the Ni(0)–bipyridine complex, which is then restored to the active catalyst by coordination with another [SeCF_3_]^−^ anion.

**Scheme 26:**
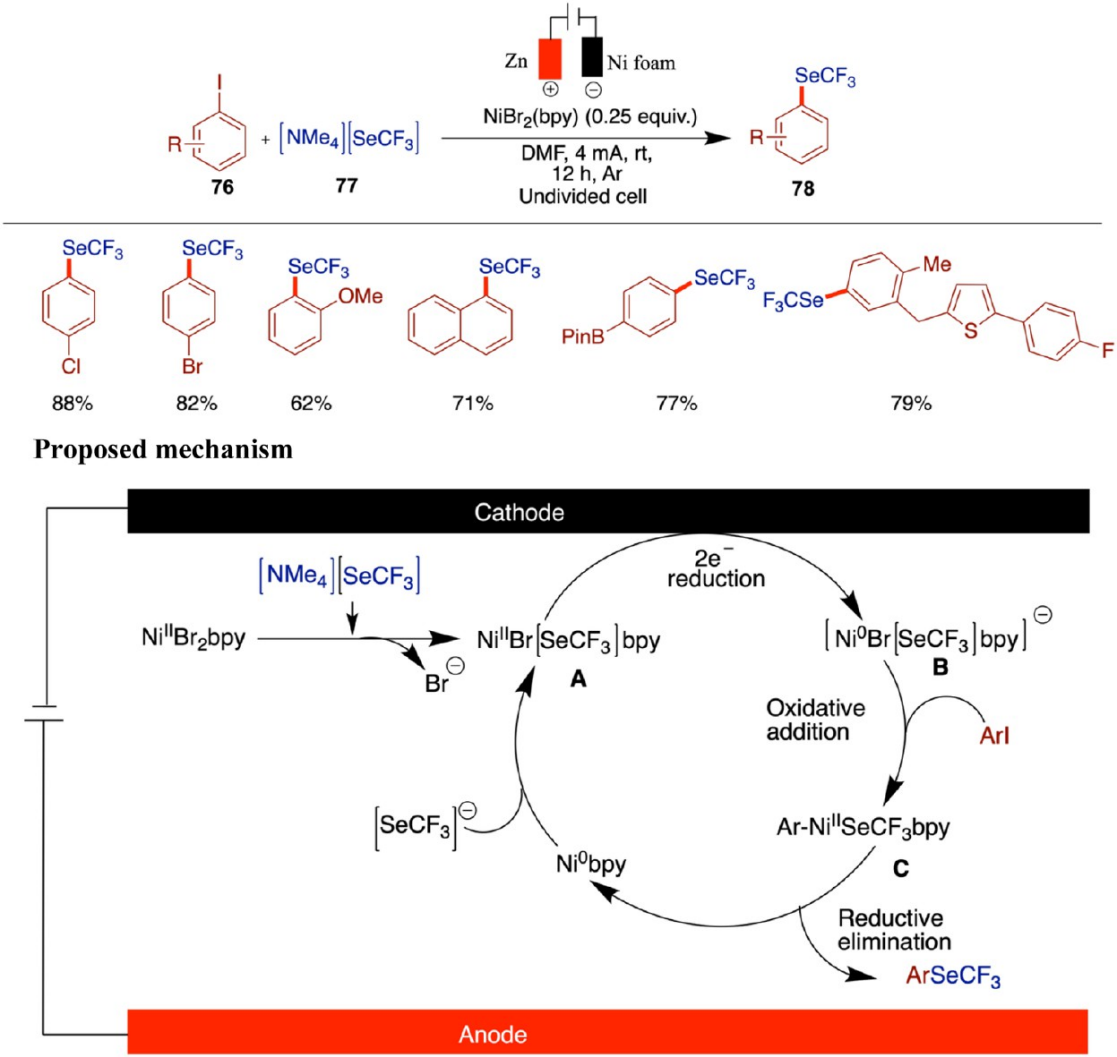
Synthesis of (Hetero)­aryl Trifluoromethyl Selenides

Aryl halides are crucial building blocks in contemporary organic chemistry and are widely used in the synthesis of pharmaceuticals and agrochemicals. Perrin and Vantourout reported a novel electrochemically driven nickel-catalyzed method for the halogenation of unsaturated halides and triflates, emphasizing the efficient exchange of halogens such as Br to Cl, I to Cl, I to Br, and OTf to Cl. Utilizing NiCl_2_·glyme as a precatalyst and 2,2′-bipyridine (bpy) as a ligand in an NMP solvent, the process leverages electrochemistry to generate a nickel species that promotes reductive elimination (Scheme 27).^72^ This technique demonstrates good functional group tolerance and is compatible with a broad range of substrates including heterocycles, dihaloarenes, and alkenes. The catalytic cycle begins with ligand exchange between NiCl_2_·glyme and bipyridine to form (bpy)­NiCl_2_ (**B**), which is reduced at the cathode to generate the active (bpy)­Ni(0) species (**C**). Oxidative addition of an aryl bromide forms a Ni­(II)–aryl intermediate (**D**) that undergoes halide exchange with TBACl to yield a Ni­(II)–aryl chloride complex (**E**). Subsequent anodic oxidation produces a Ni­(III) species (**F**), which undergoes reductive elimination to afford the aryl chloride product. The resulting Ni­(I) species (**G**) is reduced back to Ni(0) (**C**), completing the cycle. The use of NiCl_2_·DME and 2,2′-bipyridine as catalytic components offers a cost-effective and stable alternative to more sensitive and expensive nickel precatalysts like Ni­(COD)_2_. This approach simplifies the reaction setup and adheres to green chemistry principles by utilizing commercially available and less hazardous materials. The electrochemical activation allows the reaction to proceed under mild conditions, broadening the applicability to substrates with sensitive functional groups. Conducting the reaction at room temperature using a simple two-electrode setup with readily available electrode materials enhances the practicality and accessibility of the method for both academic laboratories and potential industrial applications. Mechanistic insights from cyclic voltammetry and other studies provide a comprehensive understanding of the catalytic cycle, facilitating further optimization and adaptation of this method. The substitution of heavier halogens (I or Br) or polar groups (OTf) with chlorine offers advantages in the pharmaceutical and agrochemical development. A variety of aryl bromides, vinyl bromides, aryl iodides, aryl triflates, and vinyl triflates were compatible with this reaction, although vinyl triflates afforded only poor yields.

**Scheme 27:**
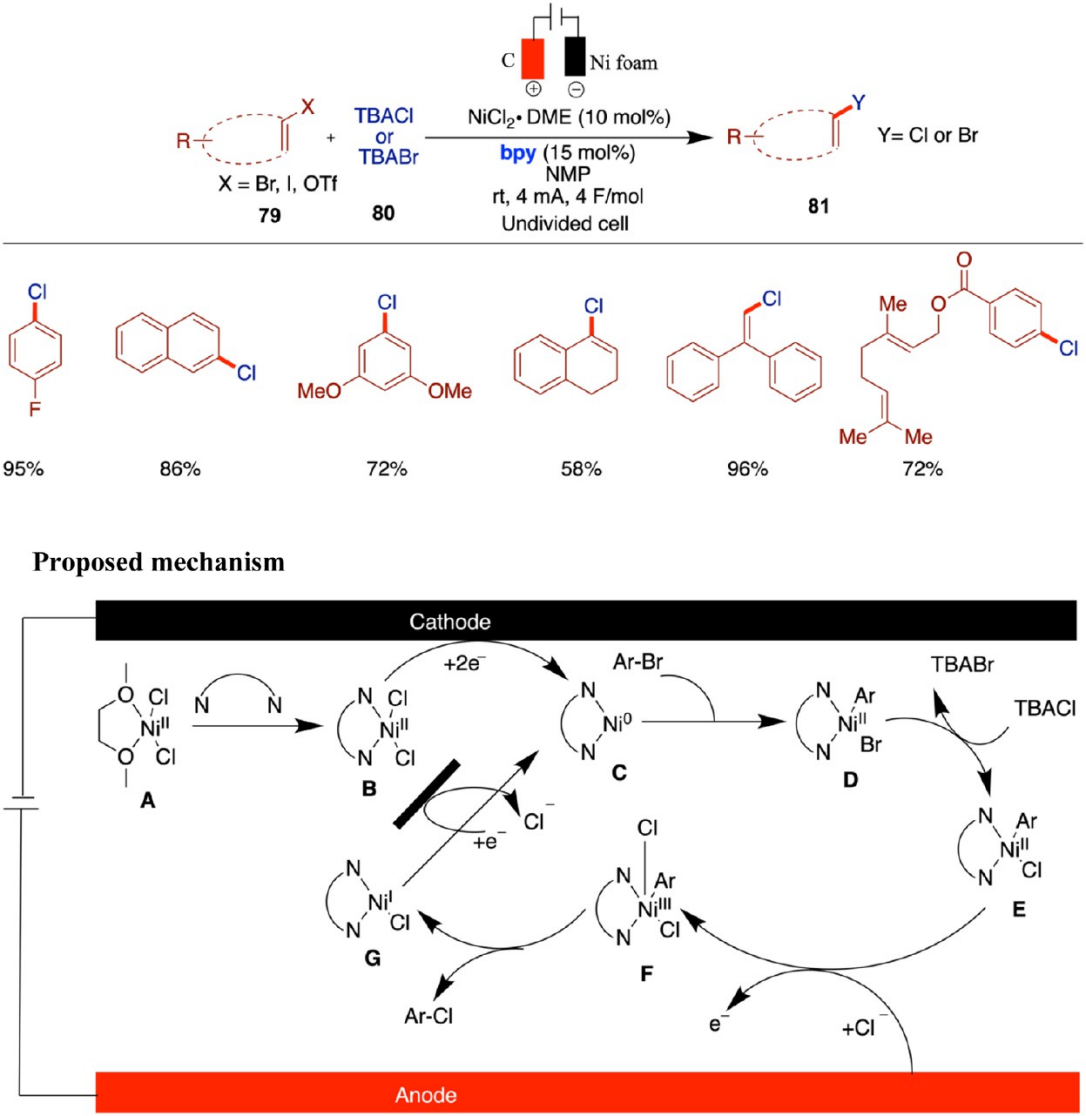
Nickel-Catalyzed Chlorination of Aryl/Vinyl Halides

## Nickel-Catalyzed Electrochemical C–S Bond Formation

The development of electrochemically enabled, nickel-catalyzed selective C–S bond-forming reactions represents a significant advancement in organic synthesis. Wang and co-workers reported an electrochemically driven, nickel-catalyzed chemoselective C–S coupling method for the synthesis of aryl sulfides and sulfones (Scheme 28).^73^ By optimizing both the nickel catalyst and the electrode materials, this electrochemical method demonstrates excellent redox performance, scalability, and sustainability. This innovative approach leverages electrochemical methods to facilitate the nickel-catalyzed formation of C–S bonds, offering a sustainable and efficient alternative to traditional synthetic routes. The process is compatible with various substrates, including heterocycles and functionalized arenes, demonstrating an excellent functional group tolerance. The catalytic cycle begins with cathodic reduction of Ni­(II) species **A** to Ni(0) complex **B**, which undergoes oxidative addition with aryl iodide to form Ni­(II) intermediate **C**. Simultaneously, anodic oxidation of sodium sulfinate generates sulfonyl radical **F**, which may disproportionate to form thiyl radical **G**. Two pathways are proposed: in Path A, **F** reacts with **C** to give Ni­(III) intermediate **D**, leading to sulfone product **85** and Ni­(I) species **E**. In Path B, **G** adds to **C** to form **D’**, ultimately yielding thioether **84** and **E**. The Ni­(I) species is then reduced at the cathode to regenerate **B**. Chemoselectivity is influenced by the nickel catalyst, while the choice of electrode and base may affect radical disproportionation.

**Scheme 28:**
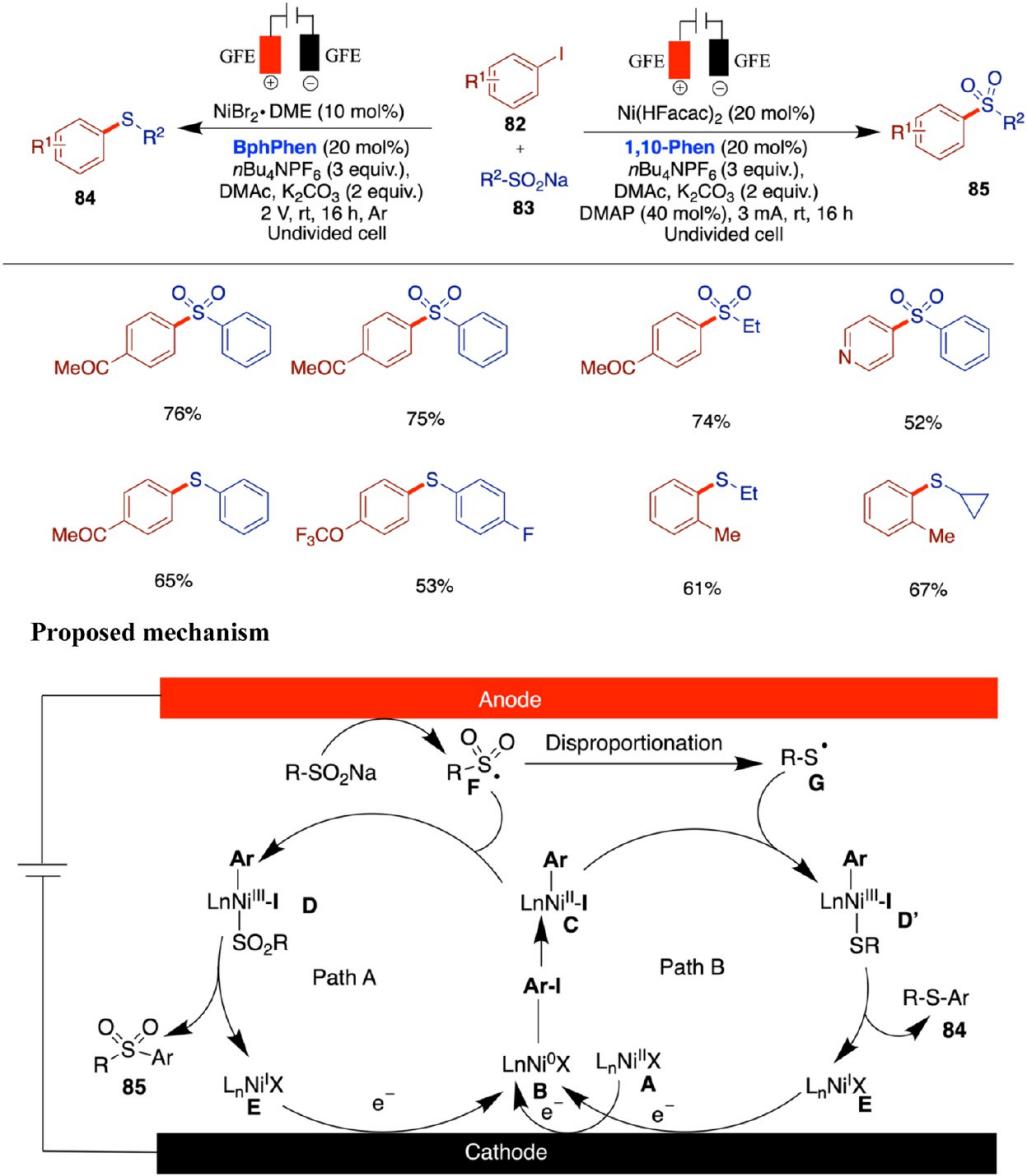
Nickel-Catalyzed Chemoselective C–S Bond Formation

Ackermann et al. reported an efficient electroreductive synthesis of thioethers from bench-stable thiosulfonates and alkyl bromides via cross-electrophile coupling, mediated by a nickel catalyst under mild reaction conditions (Scheme 29).^74^ The selection of the catalyst, electrodes, ligands, and solvents proved to be critical to reaction performance. Among the ligands tested, 2,2′-bipyridine (bpy) delivered superior yields compared with bathocuproine and neocuproine. While polar aprotic solvents such as DMF and DMA supported the transformation efficiently, variations in electrode materials, including platinum cathodes, had only a minor impact on yield. The reaction proceeds through the formation of a primary alkyl radical, as confirmed by a radical clock experiment. Cyclic voltammetry studies revealed that the bipyridine-ligated nickel catalyst is reduced at a less negative potential than the S-aryl benzenethiosulfonate substrate, indicating that the catalyst is reduced first. The substrate undergoes a two-electron reduction at more negative potentials, likely forming a thiyl radical intermediate, which can recombine to form disulfides. These findings support a sequential rather than a simultaneous reduction pathway, consistent with the observed product distribution under different applied potentials. The authors proposed a mechanism that begins with the formation of a Ni(0) complex (**A**) through cathodic reduction. This Ni(0) complex undergoes oxidative addition with thiosulfonate, resulting in the formation of an Ni­(II) complex (**B**). The Ni­(II) complex then couples with an alkyl radical generated in situ, forming a Ni­(III) complex (**C**). Reductive elimination of this Ni­(III) complex (**C**) leads to the formation of the C–S bond and releases the Ni­(I) complex. When an alkyl halide is added to this Ni­(I) complex (**D**), it generates a Ni­(II) species (**E**), which is further reduced to regenerate the Ni(0) complex, thereby continuing the catalytic cycle.

**Scheme 29:**
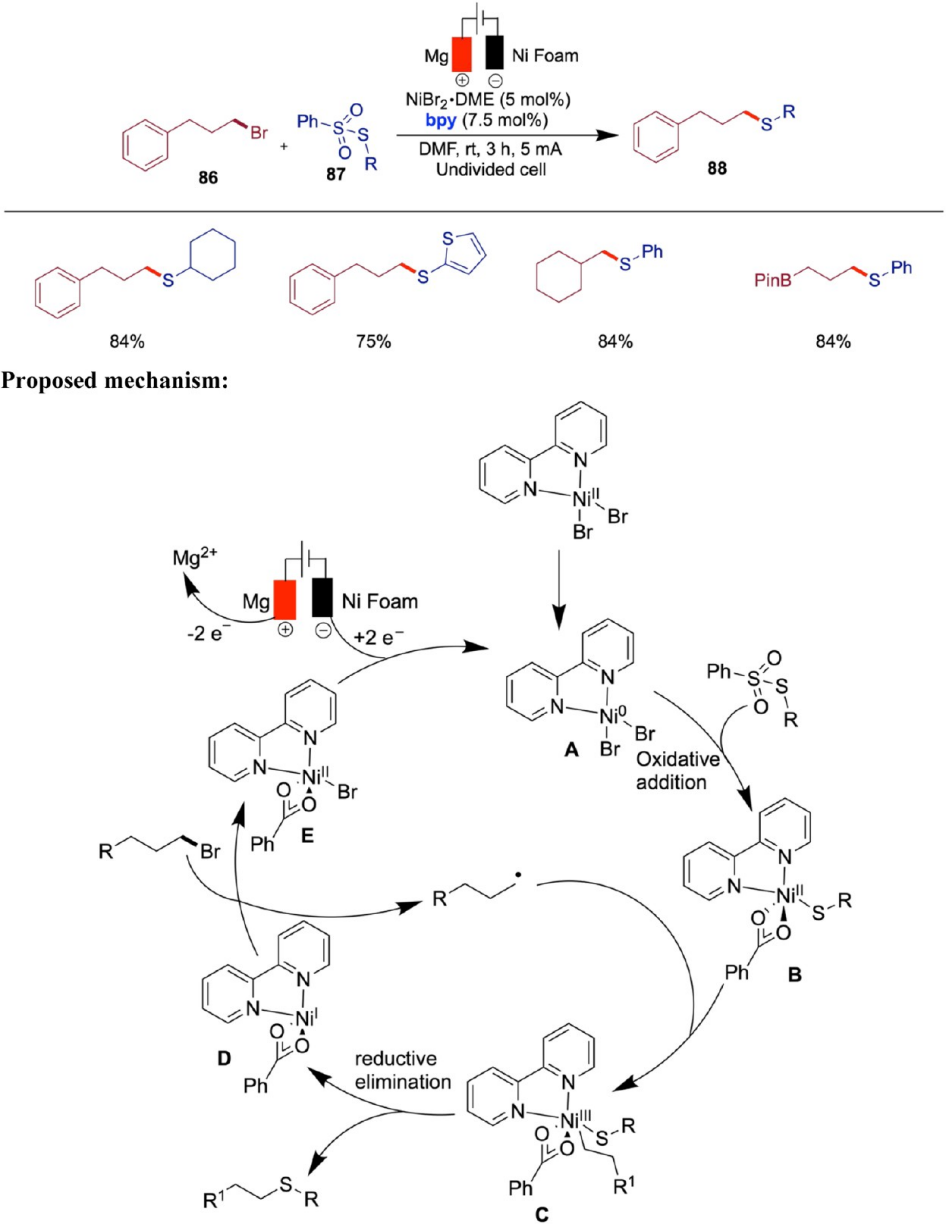
Electrochemical Synthesis of Thioethers from Thiosulfonates

In 2019, Wang and co-workers reported an electrochemical nickel-catalyzed carbon–sulfur bond formation from aryl iodides and thiols in the presence of pyridine as a base and dtbby [4,4-di-*tert*-butyl-2,2-dipyridyl] as a ligand. In this work, the authors used pyridine to abstract the proton from the arylthiol radical cation after anodic oxidation (Scheme 30).^75^ This Ullmann-type thiolation protocol demonstrates broad functional group tolerance across a range of aryl halides and thiols, delivering the corresponding thioethers in moderate to excellent yields. Functional groups such as ketone, ester, and boronate were well tolerated, and a variety of heterocycles, including thiophene, pyridine, furan, carbazole, and benzopyrazole, participated successfully. However, some limitations were noted: for instance, 2-mercaptopyridine gave only trace amounts of product, likely due to coordination with the nickel catalyst. In general, thiols bearing electron-withdrawing substituents showed enhanced reactivity compared with their electron-rich counterparts. The authors proposed a mechanism in which thiols are oxidized at the anode to generate thiyl radicals. These radicals then combine with a Ni(0) complex, which is formed via the cathodic reduction of a Ni­(II) precursor, to generate a Ni­(III)–Ar–SR intermediate. Reductive elimination from this intermediate furnishes the desired product.

**Scheme 30:**
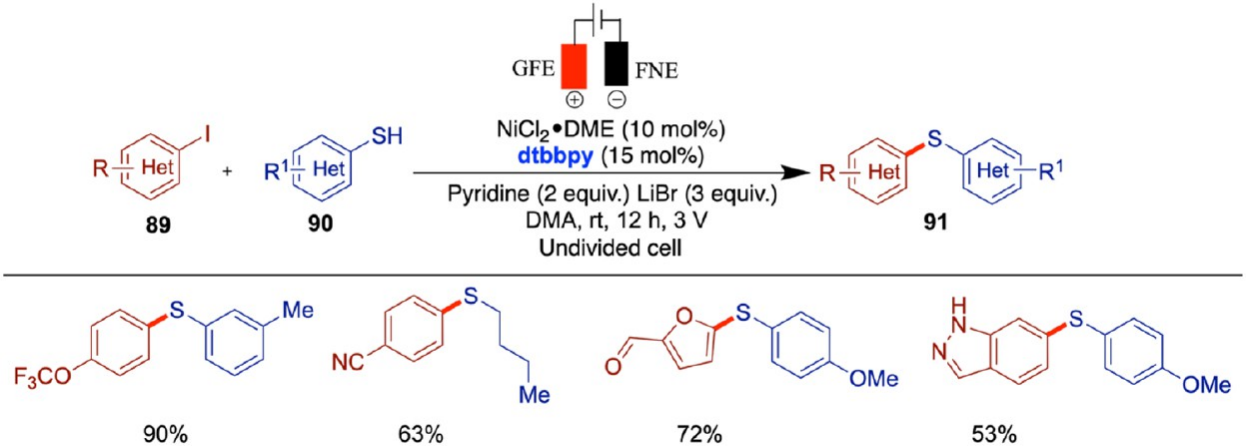
Nickel-Catalyzed Sulfenylation of Aryl Halides

Around the same time, Mei and co-workers reported the electrochemical nickel-catalyzed thiolation of aryl halides in the absence of an external base at room temperature under mild reaction conditions in an undivided electrochemical cell. Various aryl bromides and chlorides were used for the first time in nickel electrochemical catalysis to produce a range of aryl thioether products. Notably, the method is compatible with several heterocyclic scaffolds of biological relevance, including pyridine, pyrimidine, pyridazine, quinoline, indole, and benzothiophene derivatives (Scheme 31).^76^ A variety of aryl halides, including aryl chlorides, were successfully incorporated into this reaction. To some extent, the authors also included vinyl halides and heteroaryl bromides. However, the scope of thiols was limited primarily to arylthiols with the notable exception of 4-pyridyl thiol. The proposed mechanism involves the cathodic reduction of thiols to thiolates, which then add to a Ni(0) species generated via further cathodic reduction of Ni­(II) or Ni­(I). Reductive elimination from a Ni­(III) intermediate yields the desired product. The authors also suggested an alternative pathway involving thiyl radicals, similar to the mechanism proposed by Wang and co-workers.^75^

**Scheme 31:**
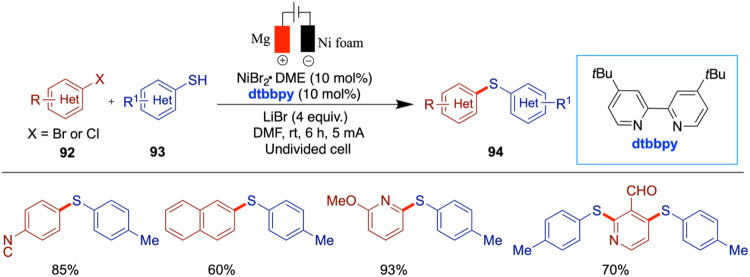
Nickel-Catalyzed Sulfenylation of Aryl Halides

In 2021, Zhang and co-workers reported a paired electrochemical nickel-catalyzed cascade reductive thiolation of aryl halides with aryl sulfinates in an undivided cell. Compared to conventional thermal and photochemical methods, this electrochemical approach offers distinct reactivity and selectivity, which arises from the paired electrolysis setup where both anodic oxidation and cathodic reduction contribute productively to the reaction outcome, enabling the direct synthesis of sulfides from sulfinates (Scheme 32).^77^ Various substituted aryl halides, including both bromides and iodides, along with aryl and alkyl sulfinates and two examples of alkyl bromides, were successfully incorporated into this thiolation reaction. The authors proposed a mechanism in which a thiyl radical is generated via the anodic oxidation of sulfinates. This radical then adds to the Ar–Ni­(II)–X complex, which is formed from Ni(0) through cathodic reduction of the Ni­(II) catalyst. The resulting RS–Ni­(III)–Ar–X complex subsequently undergoes reductive elimination to afford the desired thioether product.

**Scheme 32:**
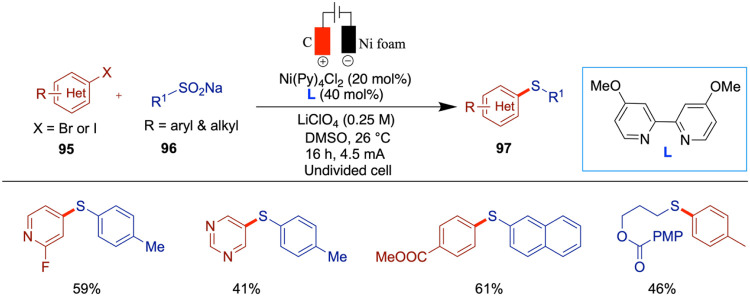
Nickel-Catalyzed Sulfenylation of Aryl Halides

The following year, Baran et al. reported an interesting study on the scalable, chemoselective nickel electrocatalytic sulfinylation of aryl halides with redox-active SO_2_. In this study, the authors employed an electrochemical oxidation strategy to generate S^VI^ analogs. Nickel-electrocatalyzed amination^83^ and etherification^59^ reactions from the same group have been reported as redox-neutral processes, whereas the sulfinylation presented here proceeds through a net reductive pathway. In this case, the electrochemical setup enables the use of a sacrificial anode in place of an external chemical reductant. Among the various SO_2_ sources screened, the DIPEA-SO_2_ adduct provided the best yield (Scheme 33).^78^ The synthetic utility of this method was demonstrated by scaling up the reaction in both batch and flow setups, yielding sulfonyl fluoride in 51% yield under batch conditions (10 mmol scale) and 50% yield in a 45 mmol scale flow reaction. These results highlight the robustness and scalability of the method across the different operational modes. Through cyclic voltammetry studies, the authors found that in the absence of DIPEA (*N*,*N*-Diisopropylethylamine) the yield was significantly reduced, suggesting the necessity of a long-lived SO_2_^•–^ radical intermediate. The proposed mechanism begins with the single-electron reduction of SO_2_, followed by the reduction of the Ni­(II) complex. The resulting low-valent Ni­(I) species undergoes oxidative addition with the aryl halide and SO_2_^•–^, forming an aryl–Ni-SO_2_ complex. Subsequent SO_2_ migration generates a Ni–aryl sulfinate intermediate, which undergoes halide substitution to release the aryl sulfinate product. Although the authors proposed a Ni­(I)/Ni­(III)/Ni­(II) catalytic cycle, they also acknowledged that alternative pathways involving other low-valent nickel species cannot be ruled out. NMPI (*N*-methylphthalimide) was identified as a reductive mediator; however, a satisfactory yield was still obtained without its presence. It is believed that NMPI fulfills two key functions: (i) it acts as a redox mediator, enhancing electron transfer efficiency during electrolysis, and (ii) it serves as an overcharge protection agent, suppressing undesired cathodic reduction of the aryl iodide substrates. While the reduction potentials of aryl iodides depend on their substituents, NMPI typically possesses a less negative reduction potential, allowing it to be preferentially reduced and thereby effectively facilitating the reaction.

**Scheme 33:**
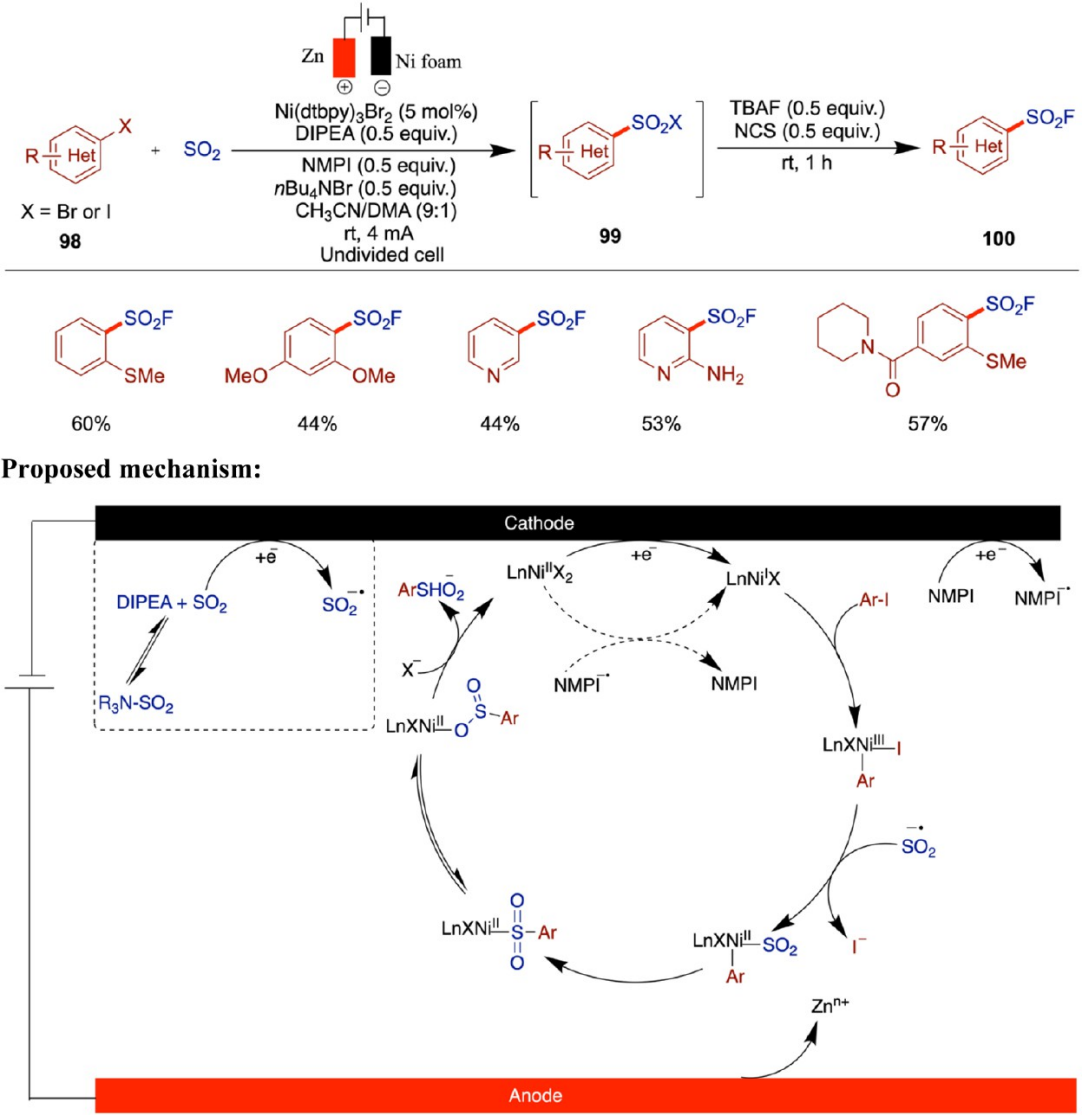
Electrocatalytic Sulfinylation of Aryl Halides with SO_2_

## Nickel-Catalyzed Electrochemical C–N Bond Formation

Carbon–nitrogen (C–N) bonds are fundamental to a vast array of biologically active compounds and advanced materials.^79^ In pharmaceuticals, C–N bonds are ubiquitous in active pharmaceutical ingredients (APIs), forming the backbone of many drug molecules.^80,81^ These structures are often critical for biological activity, receptor binding, and metabolic stability. In materials science, C–N bonds feature prominently in polymers, dyes, and agrochemicals, where they contribute to properties like thermal stability, solubility, and functionality.^82,83^ The widespread utility of C–N bonds underscores the need for efficient, sustainable, and broadly applicable synthetic methods for their formation, particularly those that align with the principles of green chemistry. Baran’s electrochemical amination has significantly impacted synthetic organic chemistry by providing a greener, more efficient alternative for C–N bond formation.^84^ Its development has spurred further research into electrochemical methods with ongoing efforts to expand its applicability, improve efficiency, and explore new types of bond-forming reactions. This approach continues to be a critical tool for chemists aiming to construct nitrogen-containing molecules in a sustainable and practical manner. In 2017, the authors’ group reported the first electrochemical protocol for cross-coupling aryl halides with alkyl amines at room temperature without the need for an external base. This methodology accommodates a wide range of substituted aryl chlorides, bromides, iodides, and triflates. Additionally, alcohols and amides were successfully employed as nucleophiles in this approach (Scheme 34). Although this method demonstrated broad applicability with various aryl halides and amines, including pharmaceutical compounds, the requirement to conduct reactions under an inert atmosphere has limited its wider use. Moreover, some limitations still remain, such as substrate-specific reactivity and the need for careful control of the electrochemical parameters. To overcome this limitation and to demonstrate that electrochemical amination could be applied to nucleosides, natural products, oligopeptides, and drug-like molecules, the authors synthesized a Ni­(bpy)_3_Br_2_ precatalyst from inexpensive starting materials in just a few steps.^85^ This catalyst proved to be highly effective for the synthesis of a wide range of functionalized amine derivatives.

**Scheme 34:**
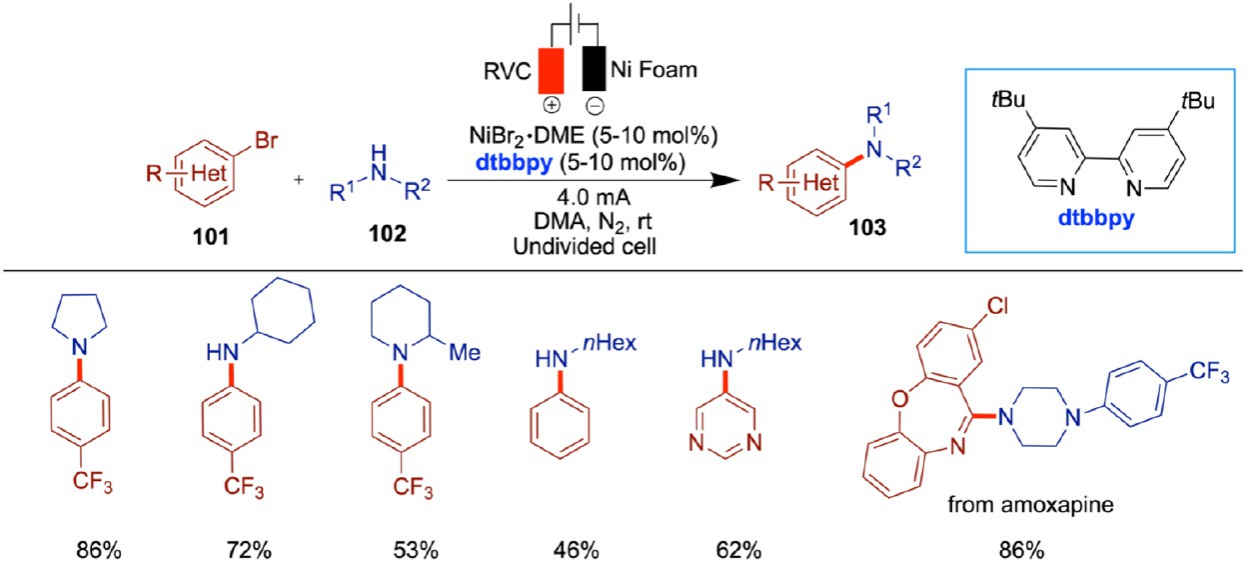
Electrochemical Amination of Aryl Halides

Sengmany and Leonel developed a method for the amination of aryl halides using electrogenerated nickel from a sacrificial anode (Scheme 35).^86^ This approach demonstrated that C–N coupling could be achieved without the need for an additional nickel catalyst and/or a special ligand system, underscoring the simplicity and efficiency of the methodology. Based on the observed reactivity, the authors proposed a plausible mechanism. The nickel catalyst generated from the sacrificial anode facilitates the oxidative addition with aryl bromide (**A**). The process involves a cathodic reduction of the Ni­(II) intermediate to generate the Ni­(I) intermediate (**B**), which then complexes with the amine to form a nickelate species (**C**). Subsequent anodic oxidation produces intermediate **D**, which undergoes reductive elimination to afford the cross-coupling product and regenerate Ni(0). Notably, the generated nickel participates in a cooperative process between the two electrodes, allowing the formation of various nickel intermediates. However, it is still possible that an alternative catalytic cycle involving the Ni­(I)/Ni­(III) system is also at play. The same group later reported an electrogenerated nickel catalyst for C–N cross-coupling, wherein nickel was generated in situ from a sacrificial anode at a constant current of 40 mA, producing approximately 10 mol % of nickel.^87^ Afterward, the sacrificial anode was replaced with a platinum electrode to continue the reaction. While the overall yields are generally high, certain amines and aryl halides resulted in low yields, and in some cases, only trace amounts of the desired product were observed.

**Scheme 35:**
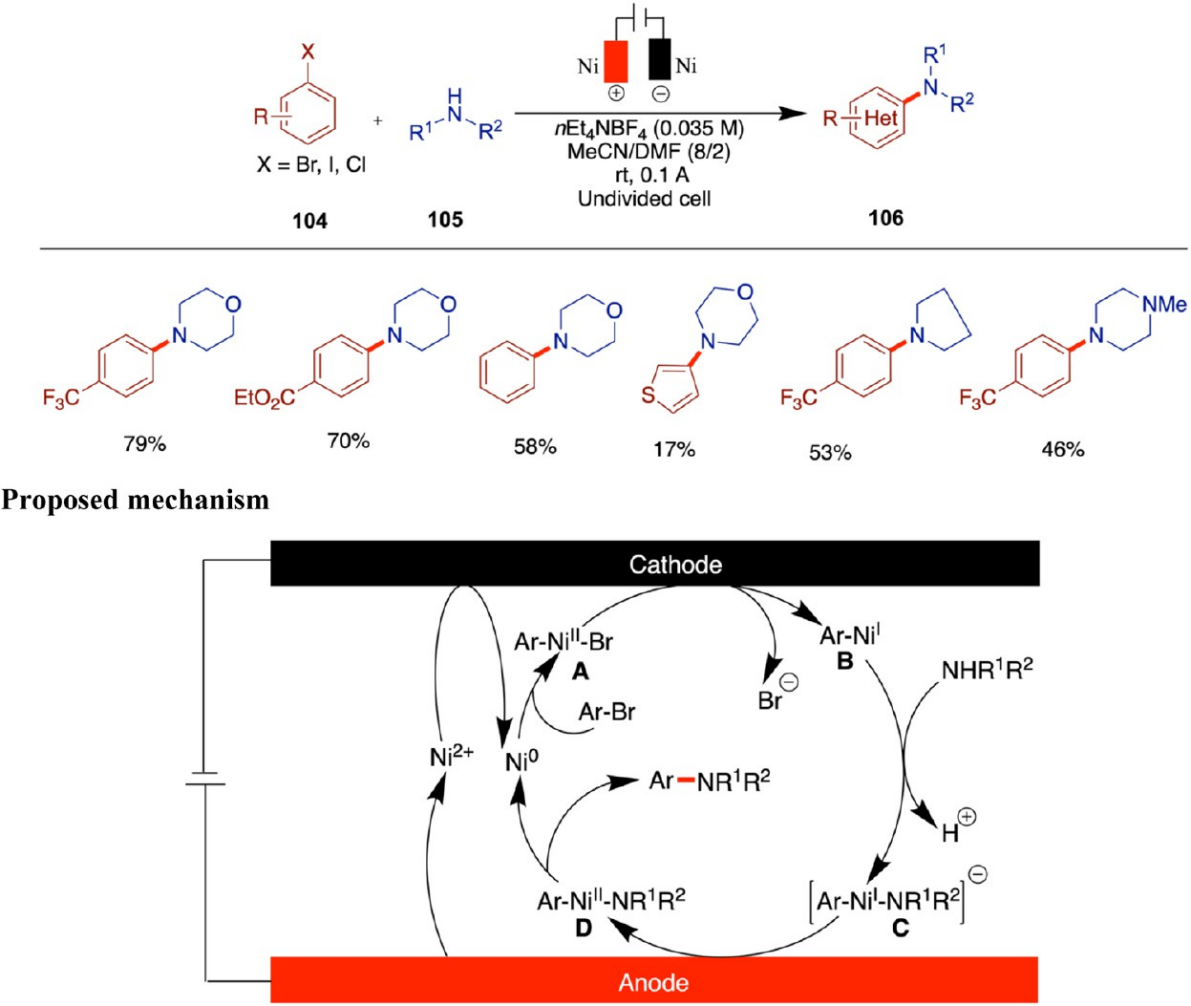
Electrochemical Amination of Aryl Halides

Mei et al. reported a nickel-catalyzed N-arylation of NH-sulfoximines with aryl halides using paired electrolysis for the synthesis of sulfoximine derivatives (Scheme 36).^88^ To eliminate the need for a sacrificial anode, the authors employed carbon-based electrodes within a paired electrolysis setup. Through a series of mechanistic studies, they discovered that the formation of the C–N bond occurs through reductive elimination from Ni­(III) species, which are generated by the anodic oxidation of Ni­(II) species, and this step is crucial for the reaction. The authors extended the application of this methodology to the late-stage functionalization of drug molecules, including Fenofibrate, Etoricoxib, Amoxapine, and Loratadine. The authors proposed a mechanism based on their cyclic voltammetric studies, starting with the formation of Ni­(I) species (**A**) through the cathodic reduction of Ni­(II). Oxidative addition then generates a Ni­(III) species (**B**). The cathodic reduction of the Ni­(III) species (**B**) produces Ni­(II) (**C**), which undergoes ligand exchange with NH-sulfoximine in the presence of a base, forming an ArNi­(III) intermediate (**D**). This intermediate subsequently undergoes reductive elimination, yielding the desired product.

**Scheme 36:**
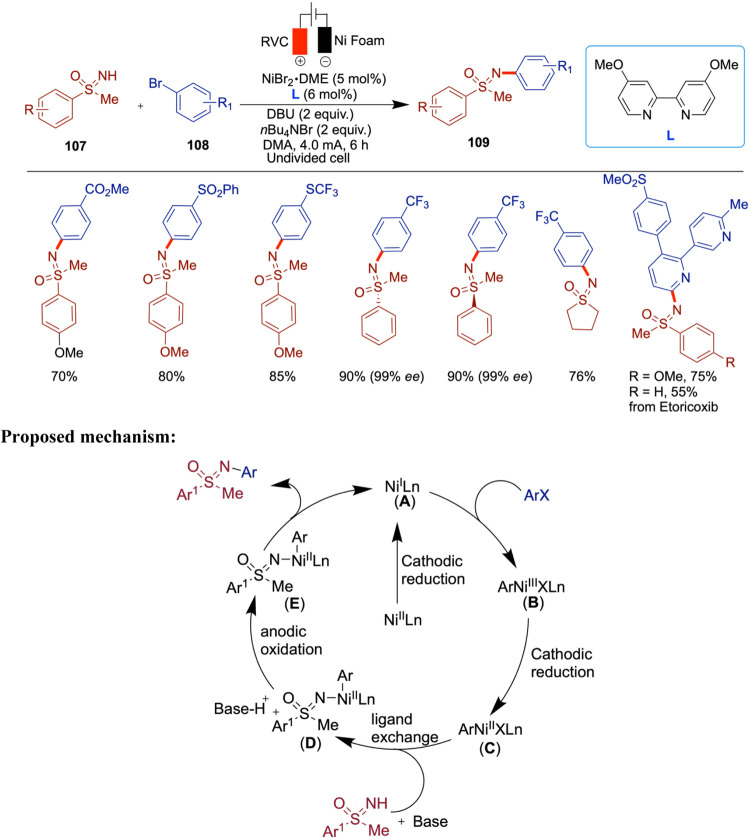
Electrochemical Amination of NH-Sulfoximines

Shang and co-workers reported an electrochemical nickel-catalyzed C­(sp^3^)–N cross-electrophile coupling between simple alkyl carboxylic acids and electrophilic amines (Scheme 37).^89^ This electrochemistry-driven decarboxylative C­(sp^3^)–N coupling method demonstrates a wide functional group tolerance across a broad range of RAEs (redox-active esters) derived from primary, secondary, and tertiary carboxylic acids. Notably, substrates bearing ester phenoxy, ketone, amino ester, and heterocyclic groups all participated efficiently in the reaction. Furthermore, sterically hindered tertiary RAEs, including α-*tert*-butyl acetic acid and complex structures derived from Gemfibrozil and Ibuprofen, were also well tolerated, delivering the desired alkyl amines in good to excellent yields. The authors proposed a mechanism that begins with the reduction of the oxime ester by a low-valent nickel species, generating the iminyl radical **B** and a nickel species referred to as Ni*^n^*. The exact oxidation state of nickel was not specified. Radical recombination of **B** and Ni*^n^* produces nickel species **C**. Subsequently, the redox-active esters undergo direct reduction at the cathode surface to generate alkyl radical **A**. Capture of this alkyl radical by species **C** leads to the formation of high-valent nickel species **D**. Finally, reductive elimination from **D** affords the C/N cross-coupled product.

**Scheme 37:**
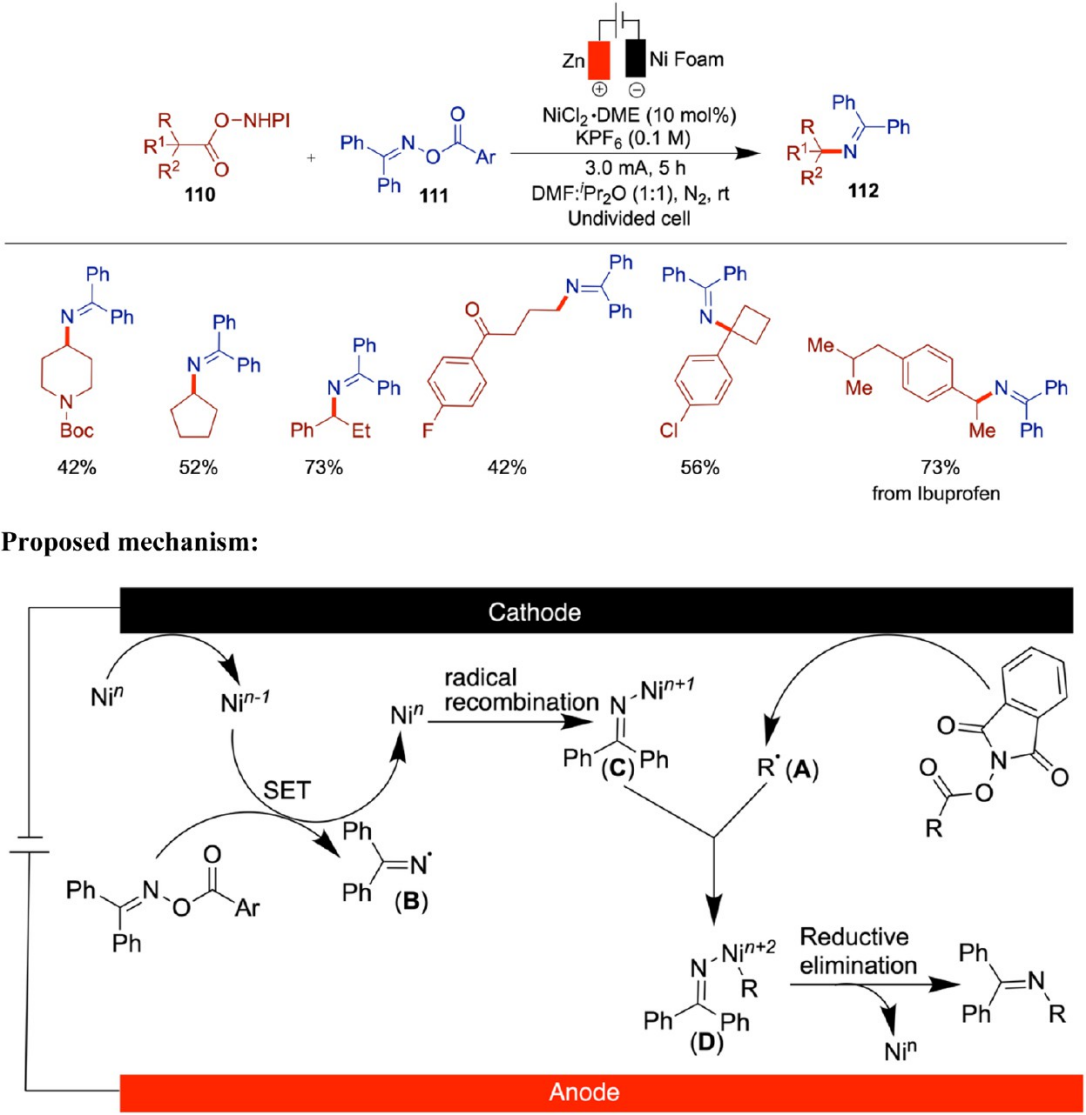
Decarboxylative C­(sp^3^)–N Cross-Electrophile Coupling

## Nickel-Catalyzed Electrochemical C–P Bond Formation

Similar to C–N bond formation, the construction of carbon–phosphorus (C–P) bonds is a fundamental transformation in organic synthesis due to the importance of organophosphorus compounds in pharmaceuticals, agrochemicals, and materials science.^90–94^ Traditional methods for C–P bond formation often require harsh reagents, transition-metal catalysts, or elevated temperatures, which can limit their sustainability and functional group tolerance.^95^ Recently, milder and more sustainable strategies, including catalytic and electrochemical approaches, have been developed to forge C–P bonds more efficiently. These modern methods improve reaction conditions, reduce environmental impact, and expand substrate scope, enhancing the practical utility of C–P bond-forming reactions in synthetic chemistry.^96^ Notably, the nickel-catalyzed electrosynthesis of aryl and vinyl phosphinates represents a significant advancement in the field of organic synthesis, particularly in the formation of C–P bonds. This method offers a more practical and scalable alternative to conventional protocols, which often rely on harsh reaction conditions, stoichiometric metal reagents, or air-sensitive organometallic intermediates. By enabling C–P bond formation under milder and more sustainable conditions, the electrochemical approach broadens functional group tolerance and enhances synthetic utility. The use of nickel catalysis under electrochemical conditions further addresses these limitations by providing a more sustainable and cost-effective strategy. Leonel and co-workers have reported nickel-catalyzed electrosynthesis of aryl and vinyl phosphinates using Fe/Ni anode, Ni cathode, bench-stable nickel precatalyst NiBr_2_(bpy) in an undivided cell (Scheme 38).^97^ Nickel catalysts facilitate the oxidative addition of aryl and vinyl halides, which is a critical step in the formation of C–P bonds. This method demonstrates successful reactivity with a variety of substituted aryl halides; however, when ortho-substituted or heterocyclic aryl halides were employed, the yields decreased significantly and, in some cases, only trace amounts of the desired products were obtained. Mechanistic studies have provided valuable insights into the role of nickel intermediates in the catalytic cycle. The formation of nickelate species through single-electron transfer and subsequent oxidative addition steps is crucial to the success of the reaction. However, the possibility of alternative catalytic cycles involving Ni­(I)/Ni­(III) species cannot be completely ruled out, indicating the need for further investigation.

**Scheme 38:**
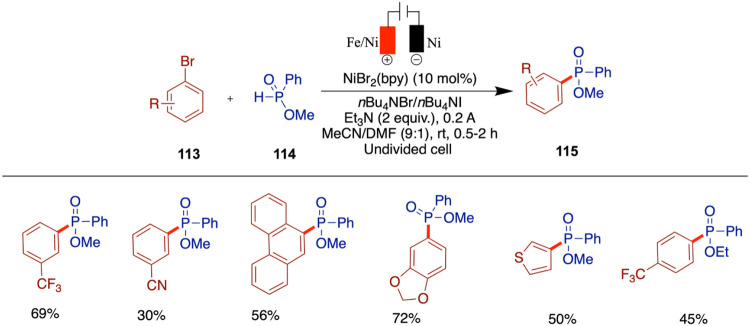
Nickel-Catalyzed Electrosynthesis of Aryl and Vinyl Phosphinates

The same group has also reported similar electrochemical nickel-catalyzed phosphorylation reactions in an undivided cell for the synthesis of various (hetero)­arylphosphonates at room temperature, using NiBr_2_bpy as precatalyst and acetonitrile as the solvent (Scheme 39).^98^ A wide range of both aryl bromides and aryl iodides has been used to provide the corresponding arylphosphonates in good yields. The yields were generally moderate to good. However, *ortho*-substituted aryl halides and aryl halides bearing electron-withdrawing groups did not afford the desired product. Additionally, the heterocyclic phosphonates containing a pyridine ring were isolated in low yield (20%), indicating possible limitations in the substrate scope. A possible mechanism begins with the initial cathodic reduction of the Ni­(II) precatalyst to generate the active Ni(0) species. This is followed by oxidative addition of the aryl halide (**A**), and subsequent single-electron reduction leads to a low-valent Ni­(I) species (**B**). The Ni­(I) species then undergoes nucleophilic attack by dimethyl phosphite to form a nickel–phosphorus ylide intermediate (**C**). Proton abstraction from this ylide generates the Ar–Ni–P­(O)­(OMe)_2_ species (**D**), which undergoes reductive elimination to furnish the final aryl phosphonate product.

**Scheme 39:**
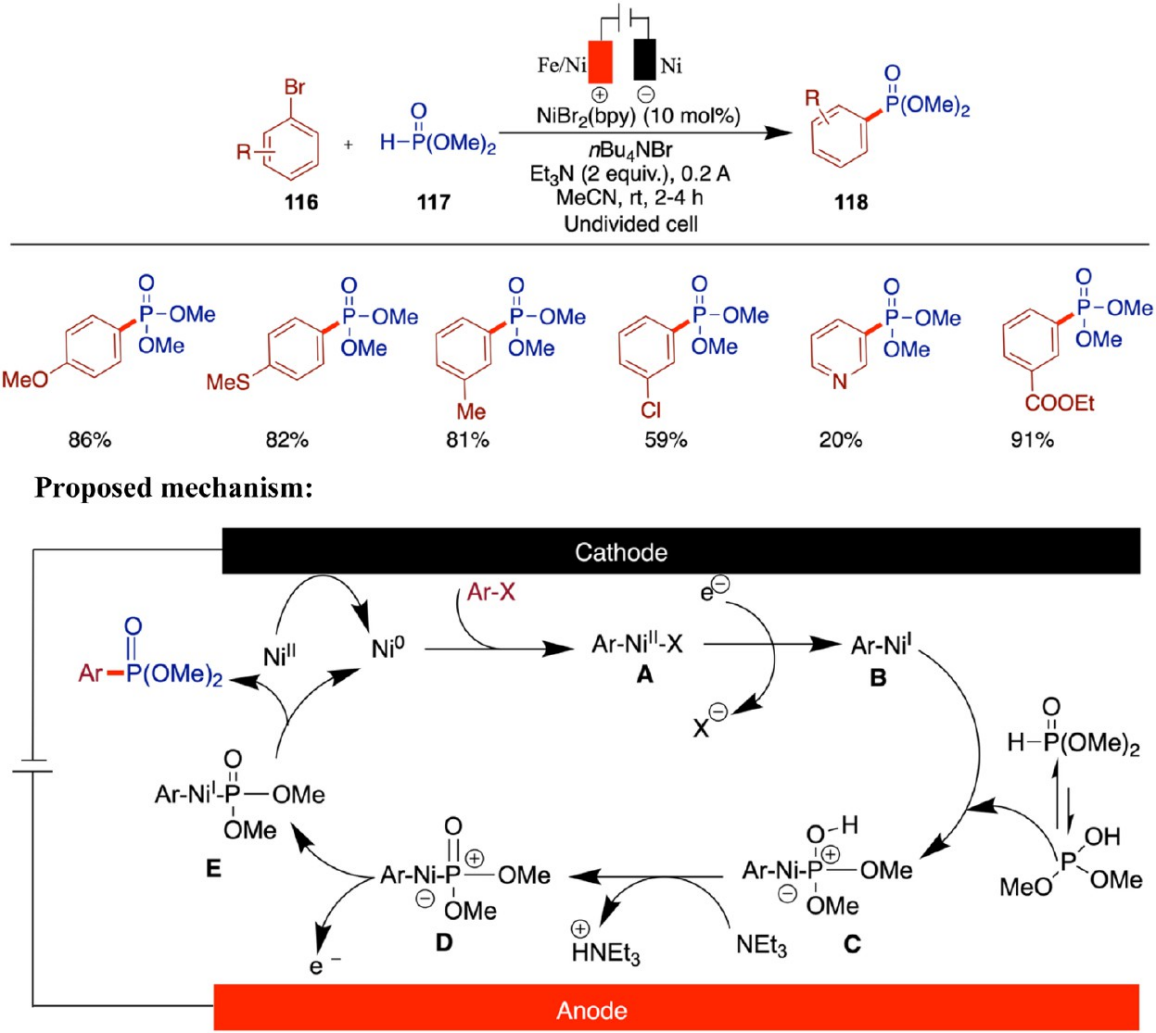
Nickel-Catalyzed Electrochemical Phosphorylation of Aryl Bromides

Xiang and co-workers have reported nickel-catalyzed electrochemical phosphorylation of aryl bromides using nonsacrificial carbon electrodes in an undivided cell (Scheme 40).^99^ This reaction is similar to those aforementioned but differs in several key aspects, including the choice of base, catalyst, and electrodes. Notably, in this study, the authors successfully included heterocyclic aryl halides and obtained moderate yields. In their proposed mechanism, the Ni^II^ catalyst under cathodic reduction generates the Ni^0^ catalyst, which further undergoes oxidative addition with aryl halides to provide the Ar–Ni^II^-Br intermediate (**A**). This intermediate is immediately coupled with the radical that is generated from the anode to give the Ni^III^ intermediate (**B**). From this intermediate the reductive elimination gave the product and the active catalyst was again generated by electrochemical conditions, which complete the catalytic cycle.

**Scheme 40:**
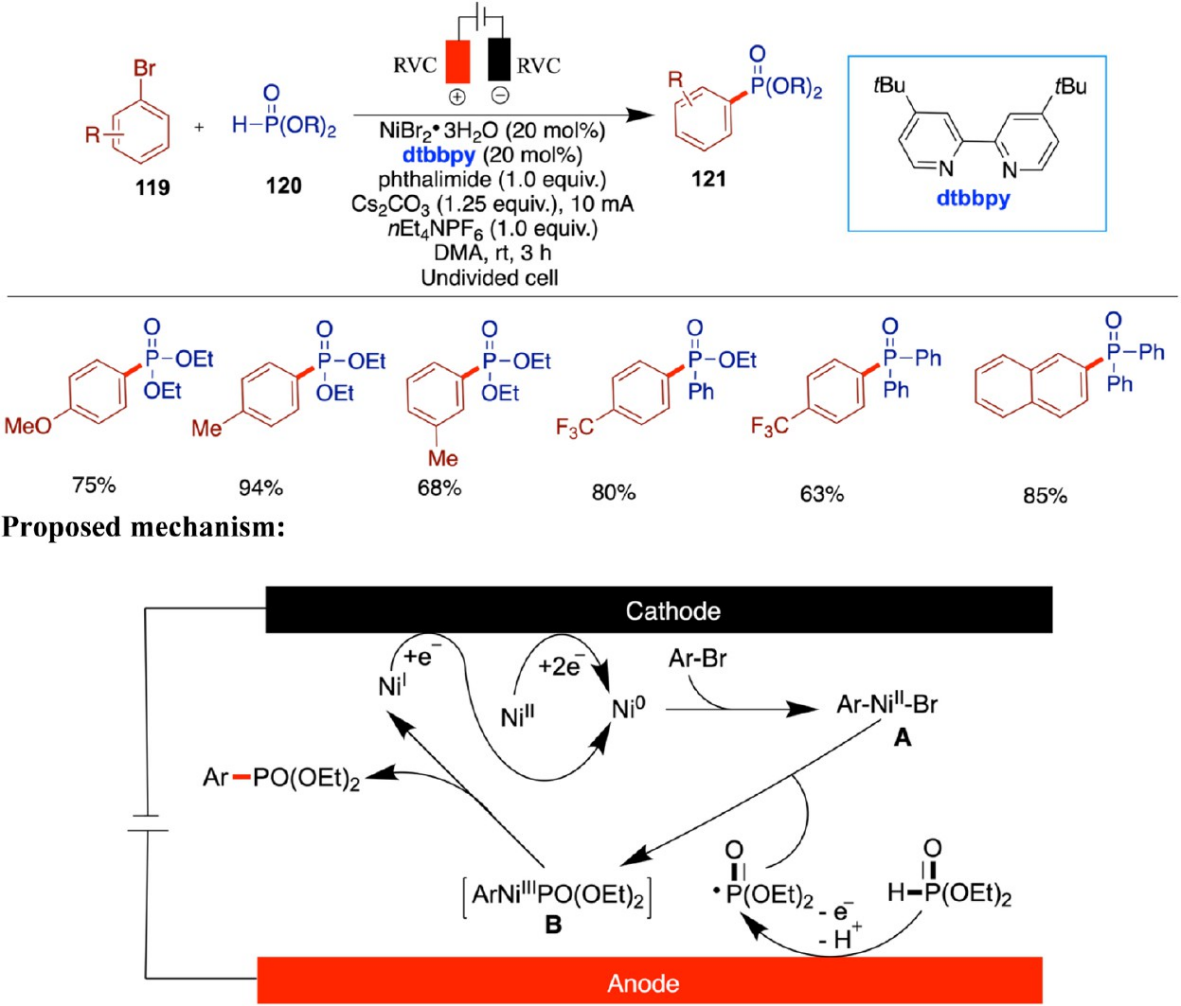
Nickel-Catalyzed Electrochemical Phosphorylation of Aryl Bromides

Cheong and Park presented a unified electrochemical strategy for [2 + 2+2] cyclotrimerizations, enabling the synthesis of 1,3,5- and 1,2,4-trisubstituted benzenes through the Ni­(I)-mediated reduction of alkynes (Scheme 41).^100^ By adjustment of the reaction conditions, the authors were able to control the regioselectivity of the trimerization products. This method is compatible with a wide range of substituted alkynes, although few substrates resulted in low yields. The 1,3,5-selective products were obtained using sacrificial Ni electrodes in the presence of pivalic acid (4 equiv), while the 1,2,4-products were formed using a Ni­(ClO_4_)_2_ catalyst. The selectivity of the cyclotrimerization reaction can be precisely controlled, depending on the nature of the alkyne substituent. Different substituents can influence the reaction pathway, directing the formation of either 1,3,5- or 1,2,4-trisubstituted benzene products. By carefully choosing the alkyne substituent and optimizing the reaction conditions, it is possible to achieve a high degree of regioselectivity, tailoring the product distribution according to the desired outcome.

**Scheme 41:**
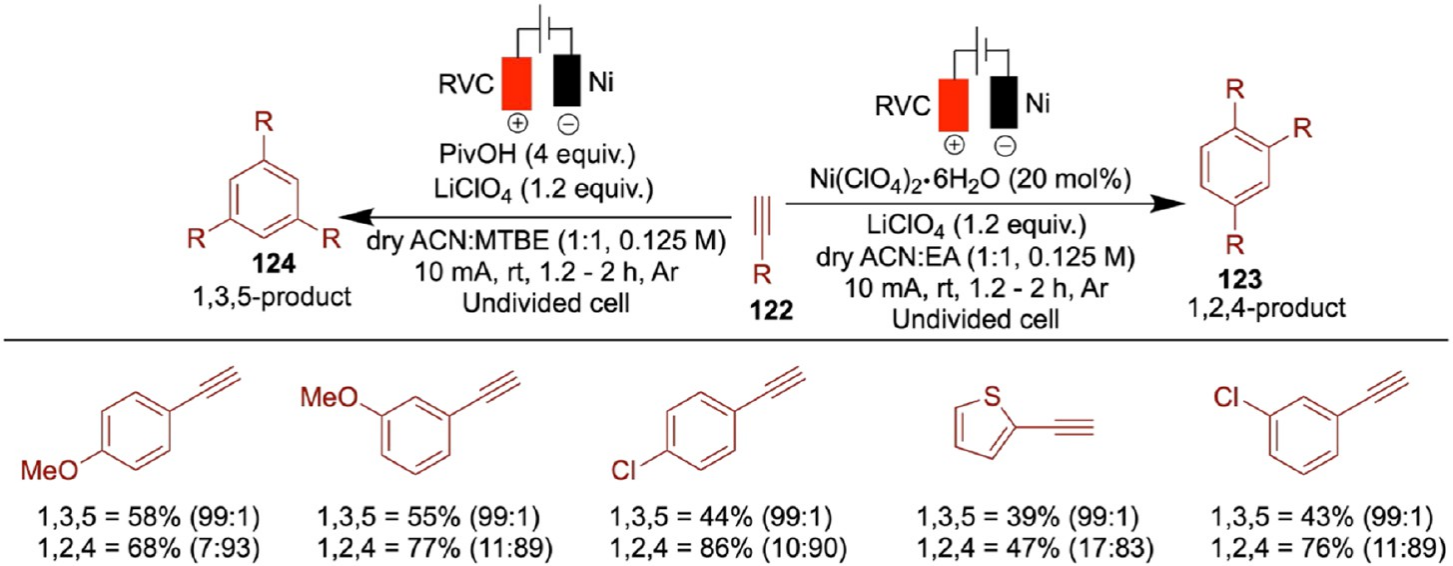
Electrochemical [2 + 2+2] Cyclotrimerizations

Wang and co-workers have reported electrochemical nickel-catalyzed ligand free site selective 1,2-diarylation of 1,3-dienes.^101^ Various aryl iodides and 1,3-dienes were successfully employed in this protocol. While the authors aimed to achieve enantioselective arylation using chiral ligands, the enantioselectivity was generally low, with some cases showing no enantioinduction at all. Regarding substrate scope, although a few examples gave good yields, most of both aryl iodide and 1,3-diene derivatives afforded only moderate yields (Scheme 42).

**Scheme 42:**
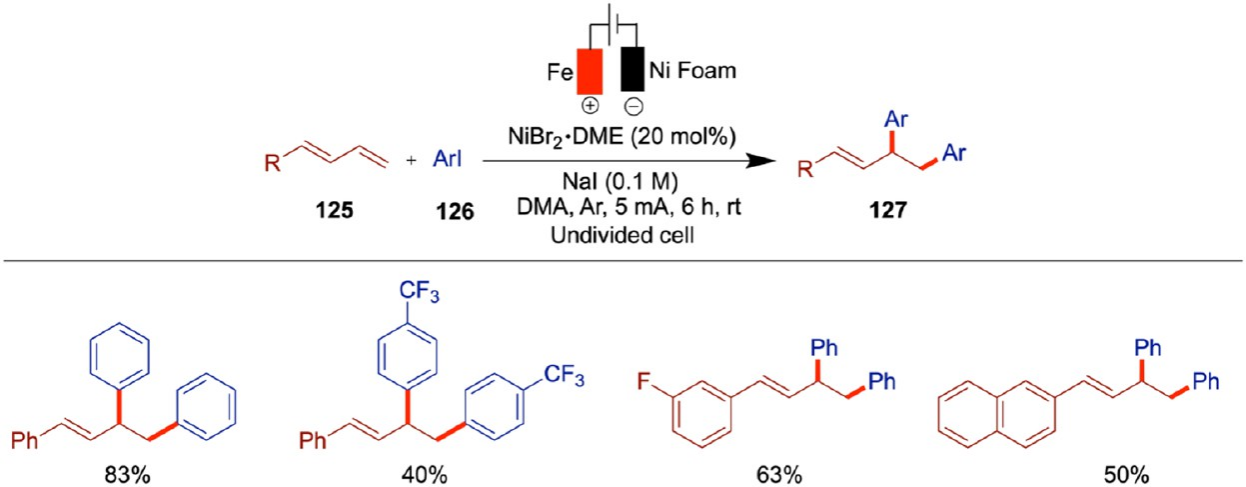
Site Selective 1,2-Diarylation of 1,3-Dienes

The same group reported the allylation of aryl halides via an electrochemical nickel-catalyzed cross-coupling process. They described a nickel-catalyzed reductive cross-coupling of aryl or alkyl halides with various allylic reagents. This method includes the use of trifluoroalkenes, sulfonates, oxalates, acetates, and MBH esters under electrochemical conditions. Simple aryl halides afforded good yields, whereas sterically hindered (bulky) aryl halides generally resulted in only moderate yields. The authors proposed a plausible mechanism that begins with the initial cathodic reduction to generate Ni(0) species (**A**), which then undergoes oxidative addition of PhI to form a Ni­(II) species (**B**). This Ni­(II)-Ph species (**B**) upon further cathodic reduction, generates intermediate **C**, which subsequently undergoes migratory insertion with an allyl derivative to yield a nickel­(I) intermediate (**D**). This intermediate then undergoes β-fluoroelimination to form the desired product, followed by cathodic reduction to regenerate the active Ni(0) species (**A**), allowing the catalytic cycle (Scheme 43).^102^

**Scheme 43:**
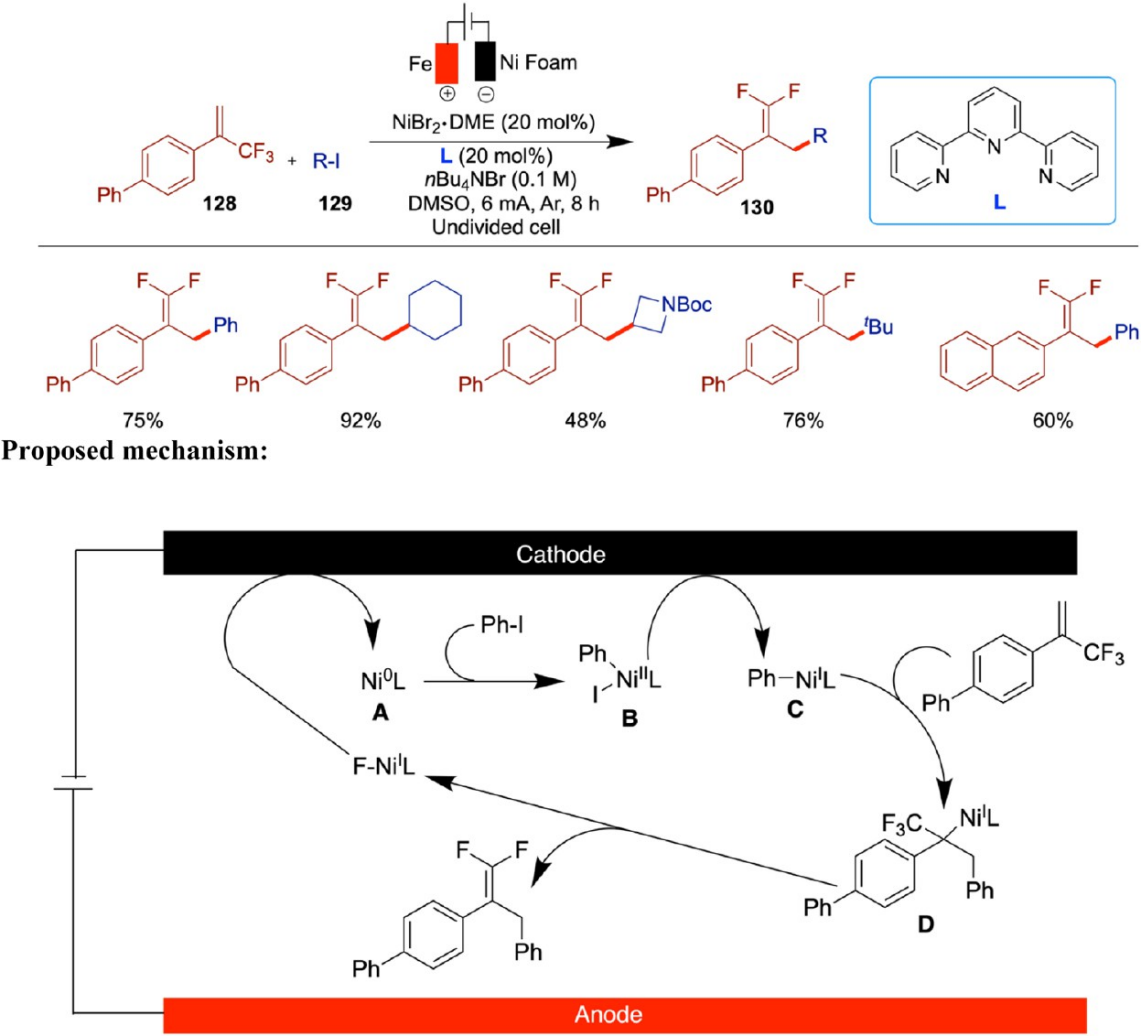
Nickel-Catalyzed Reductive Cross-Coupling of Aryl/Alkyl Halides with Allyl Reagents

Sevov and co-workers reported an electrochemical nickel-promoted Mizoroki–Heck coupling of aryl halides with alkenes (Scheme 44).^103^ The substrate scope in this reaction and yields are generally good to moderate, with the exception of sulfone-substituted alkenes giving low yields. The authors hypothesized that the kinetically inactive Ni­(II) aryl complex would become active only in the Heck reaction in the presence of a low-valent Ni catalyst, which initially led to low yields. Recognizing the inactivity of the Ni­(II) complex, they decided to employ electrochemical reduction to activate it. This approach leveraged electrochemistry to generate relevant nickel species in situ without the need for external reductants. Additionally, this method demonstrated for the first time the successful use of alkyl alkenes in an electrochemical Heck reaction, making it suitable for the functionalization of more affordable alkenes. This innovation broadens the scope of electrochemical functionalization, offering a cost-effective and versatile approach for organic synthesis.

**Scheme 44:**
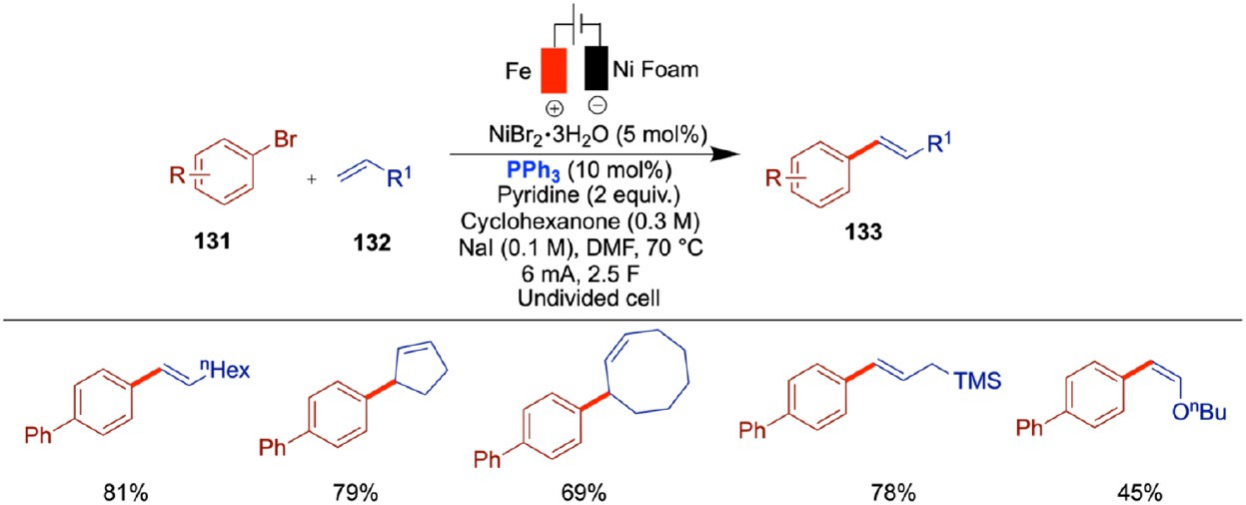
Nickel-Catalyzed Electrochemical Alkenylation of Aryl Halides

A further example of alkene functionalization through electrochemical nickel catalysis was reported by Rueping et al., a three-component reductive protocol for group 4 element heterofunctionalization of 1,3-enynes. In this important method, silyl-, germanyl-, and stannyl alkylation of enynes was demonstrated (Scheme 45).^104^ The yields were generally good to moderate, and an impressive substrate scope was demonstrated, highlighting the broad applicability of this method. The study encompassed a wide range of chlorosilanes and alkynes, various alkyl bromides (1, 2, and 3°), as well as germanyl and stannyl chlorides with diverse electronic and steric properties. In the proposed mechanism, the alkyl radical adds to the terminal carbon of the 1,3-enyne to generate allenyl radical **A**, which is intercepted by Ni­(I) species to form Ni­(II) intermediate **B**. Cathodic reduction of **B** gives Ni­(I) species **C**, which undergoes oxidative addition with silyl chloride to form Ni­(III) intermediate **D**. Subsequent reductive elimination from Ni­(III) intermediate **D** furnishes the cross-coupling product.

**Scheme 45:**
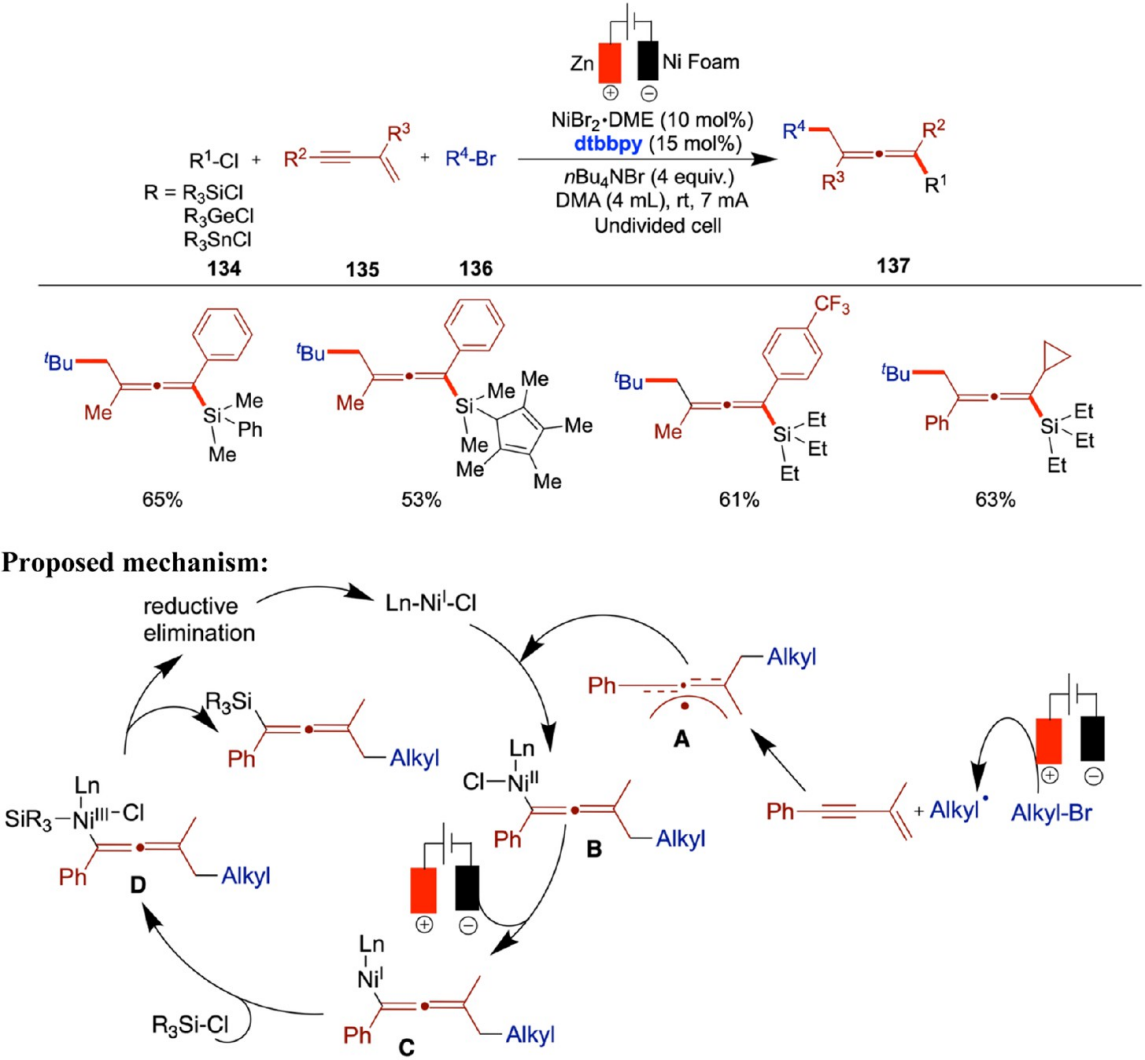
Nickel-Catalyzed Electrochemical Allene Synthesis

Liu et al. reported electroreductive nickel-catalyzed allylation of aryl chlorides using readily available allylic sulfones (Scheme 46).^105^ Traditionally, the allylic coupling used to be made by the use of various transition-metal catalysts in the presence of reductants. But here using electricity, the authors performed the same reaction without the use of an external reductant that makes this reaction very attractive. Moreover, the authors used aryl chlorides for the allylation reaction, which makes a significant advantage when we consider the cost effectiveness, when compared to aryl bromide and iodide derivatives. Using this method, the authors could synthesize various substituted allyl derivatives in good yields. The authors proposed a mechanism in which the Ni­(II) catalyst is reduced at the cathode to form Ni(0). After oxidative addition, the allylic sulfones react with Ni(0) to produce a π-allyl-Ni­(II) species (**A**), which is further reduced at the cathode to Ni­(I). This Ni­(I)-π-allyl intermediate (**B**) then undergoes oxidative addition with aryl chloride, forming a Ni­(III) intermediate (**C**). Reductive elimination from this intermediate yields the product and regenerates low-valent Ni­(I), which is subsequently reduced at the cathode to re-enter the catalytic cycle.

**Scheme 46:**
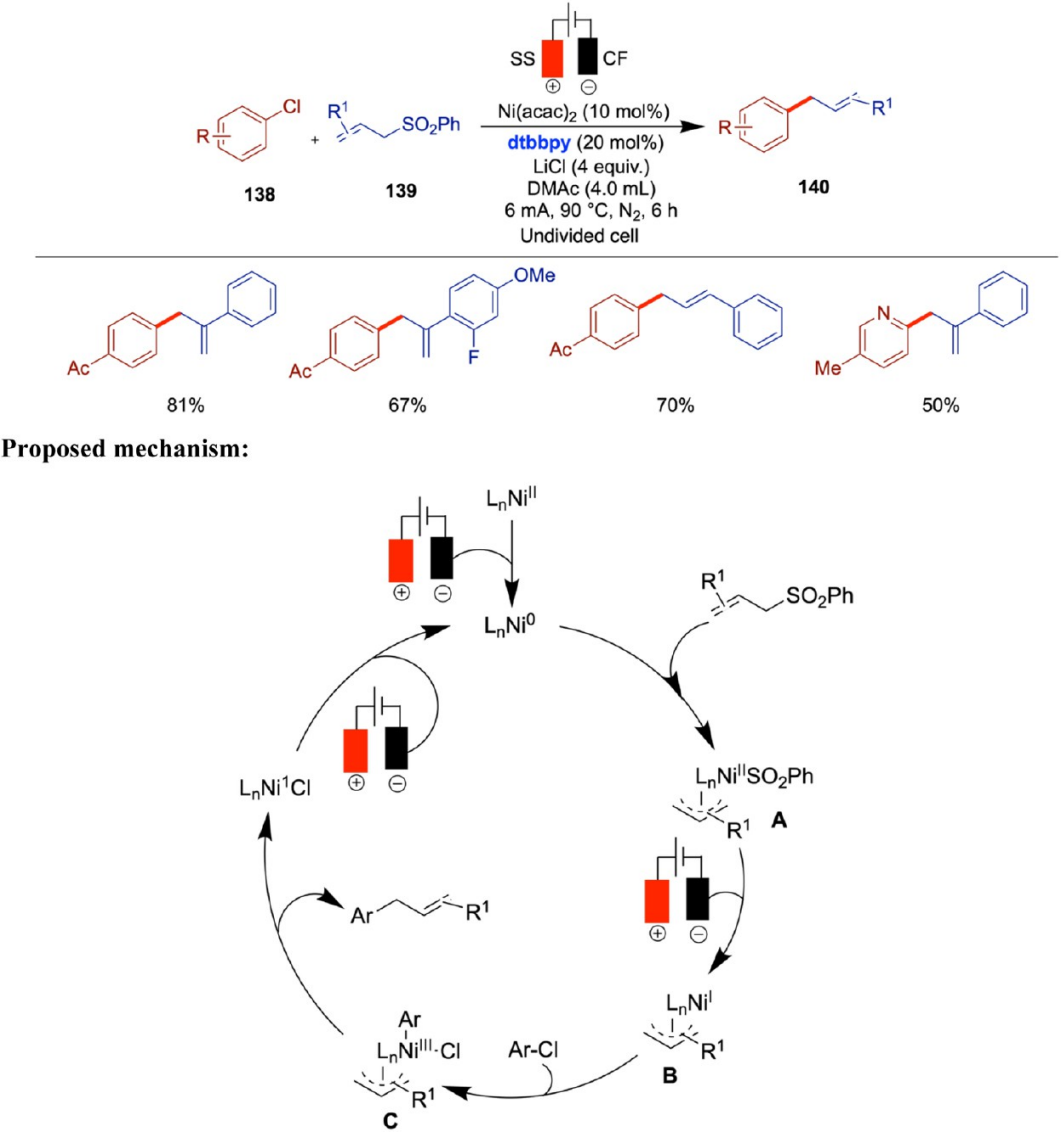
Nickel-Catalyzed Allylation of Aryl Chlorides with Allylic Sulfones

Fu and co-workers reported nickel-catalyzed electrochemical esterification of aryl halides with carbazates, which is paired with the iron catalyst to generate alkoxycarbonyl radical (Scheme 47).^106^ By introducing the iron oxidase catalysis, the authors successfully esterified various substituted aryl halides. The authors proposed a plausible mechanism in which the initial anodic oxidation of methyl carbazate, facilitated by an iron catalyst, produces a methoxycarbonyl radical (**A**). Meanwhile, the arylnickel species (**B**), formed via cathodic reduction and subsequent oxidative addition in solution, is sufficiently stable to persist in the bulk medium. At this stage, the carbon-centered radical (**A**) adds to the nickel complex (**B**), and the coupling product is formed through reductive elimination.

**Scheme 47:**
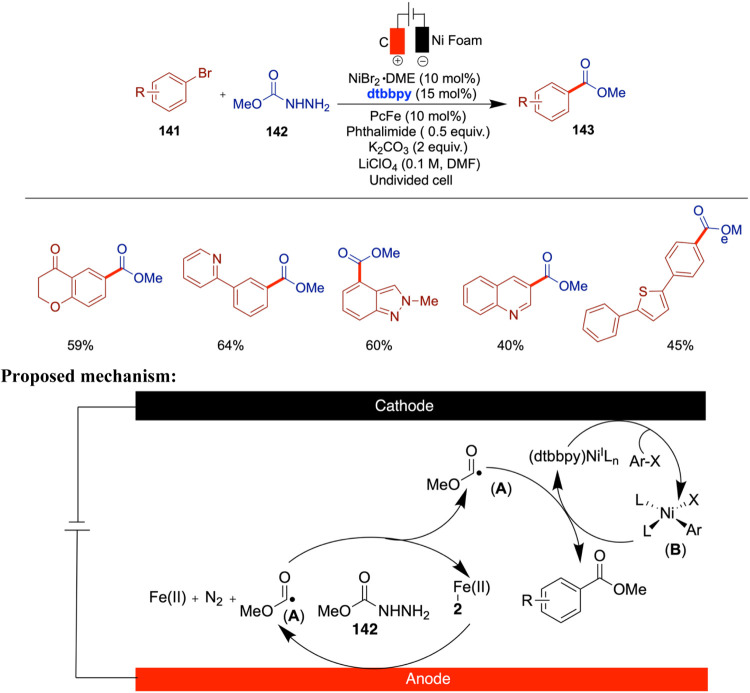
Nickel-Catalyzed Electrochemical Esterification of Aryl Halides with Carbazates

## Nickel-Catalyzed Electrochemical Fluoroalkylation

Recent advances in catalysis, particularly with transition metals, have made fluoroalkylation more efficient and selective, enabling the synthesis of complex molecules with precision.^107^ The development of environmentally friendly and sustainable fluoroalkylation methods is also an ongoing area of research.^108–110^ Electrochemical methods for the fluoroalkylation have been developed.^111,112^ For example, Qiu and co-workers reported a defluoroalkylation of *gem*-difluoroalkenes with unactivated alkyl halides to synthesize monofluoroalkenes, which is extended into late-stage functionalization of drugs (Scheme 48).^113^ The formation of alkyl radical intermediate, as confirmed by mechanistic studies was found to be a crucial step, and all the observed products found with high *Z*-stereoselectivity. The authors proposed a mechanism involving an initial reduction of Ni­(II) at the cathode to Ni(0), which then undergoes oxidative addition with alkyl halides, forming a Ni­(II) alkyl complex **A**. This complex **A** undergoes migratory insertion with gem-difluoroalkenes, generating complex **B**. Complex **B** then undergoes β-fluoroelimination, producing the desired product and leaving behind a Ni­(II) complex, which is reduced cathodically to regenerate Ni(0) and continue the catalytic cycle.

**Scheme 48:**
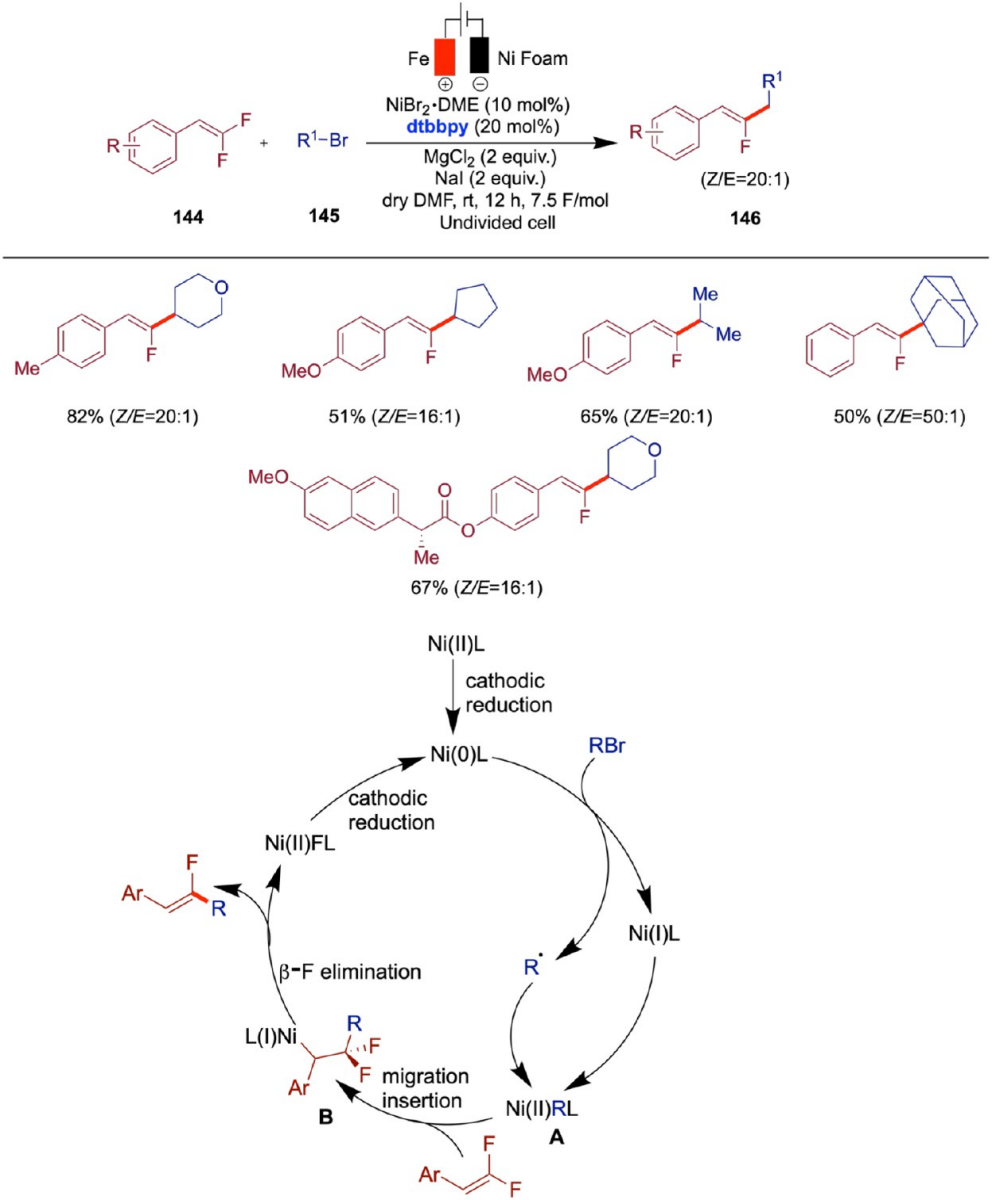
Electrochemical Fluoroalkylation

Zhang and Wang et al. reported a paired electrochemical nickel-catalyzed oxidative fluoroalkylation of aryl iodides (Scheme 49).^114^ In this report, the authors used sodium difluoromethanesulfonate as a source to produce difluoromethane radical at the anode, which coupled with the aryl iodide for the target synthesis. The authors proposed a mechanism involving two distinct pathways. In the first pathway, the oxidative addition of Ar–I to Ni­(I) generates an Ar–Ni­(III)–Rf intermediate, which is subsequently reduced cathodically to Ni­(II). The second pathway begins with the cathodic reduction of Ni­(I) to Ni(0), which undergoes oxidative addition with Ar–I to form an Ar–Ni­(II)–I species. Simultaneously, anodic oxidation of sodium fluoroalkyl sulfinate generates a fluoroalkyl radical, which is intercepted by the Ar–Ni­(II)–I complex to form the Ar–Ni­(III)–Rf intermediate. Reductive elimination from Ni­(III) then furnishes the fluoroalkylated product and regenerates the Ni­(I) species.

**Scheme 49:**
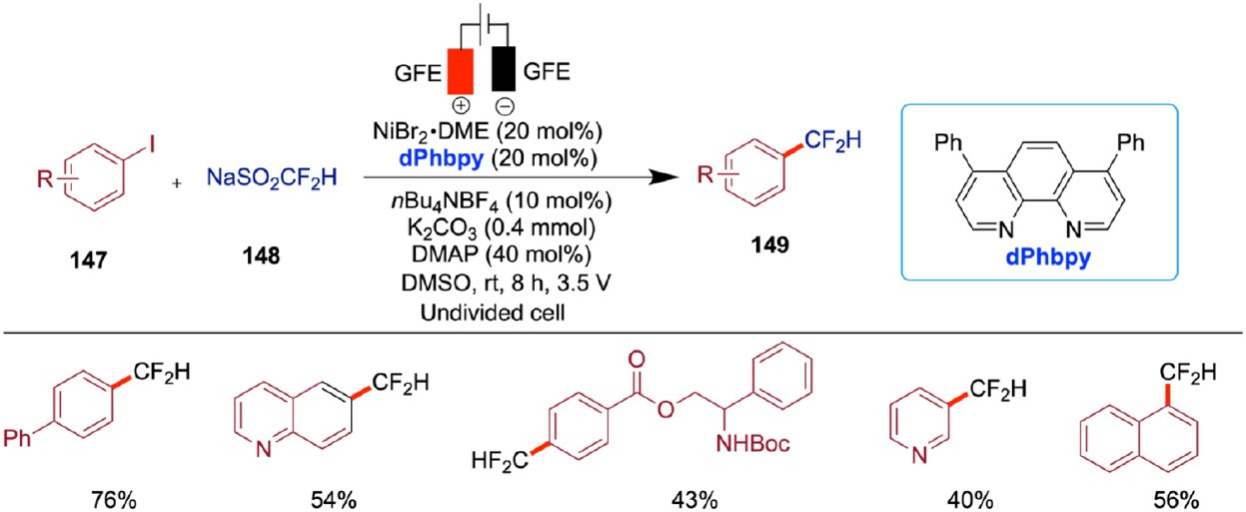
Nickel-Catalyzed Oxidative Fluoroalkylation of Aryl Iodides

## Nickel-Catalyzed Electrochemical Peptide Functionalization

Recently, Messaoudi and co-workers reported a nickel-catalyzed electrochemical method for the selective inter- and intramolecular arylation of cysteine-containing peptides (Scheme 50).^115^ This approach demonstrated the effectiveness of electrochemistry for synthesizing linear and cyclic S-arylated peptides without the need for a base. The base-free reaction highlights the potential of nickel electrocatalysis in peptide modification with possible applications in chemical biology. This methodology was successfully applied to peptides containing sensitive functional groups, such as NH_2_ and OH groups.

**Scheme 50:**
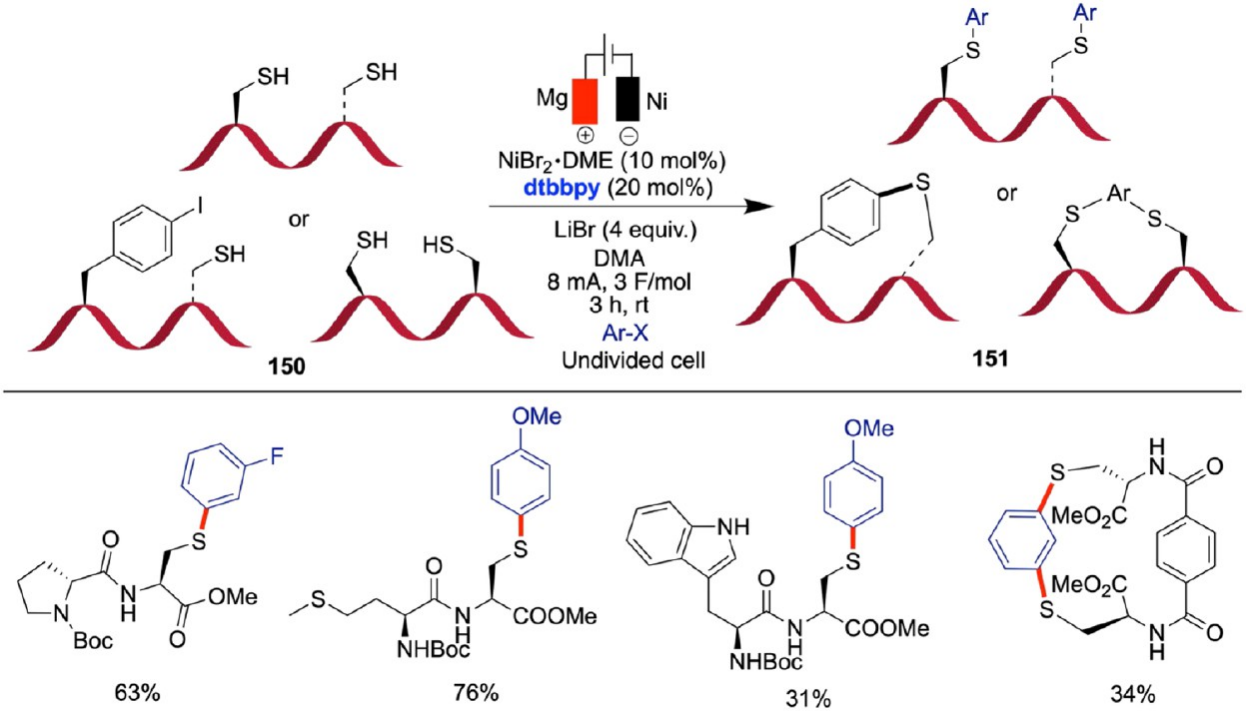
Electrochemical Arylation of Cysteine-Containing Peptides

## Nickel-Catalyzed Electrochemical Intramolecular Cyclization

Durandetti et al. reported an intramolecular nickel-catalyzed electrochemical cyclization of alkynyl aryl iodides via carbometalation of a triple bond with an aldehyde, using an aluminum sacrificial electrode (Scheme 51).^116^ This approach enabled the synthesis of heterocycles with high stereoselectivity and good yields. Cyclic voltammetry studies suggest that the reaction proceeds through a synexo-dig cyclization process. The authors proposed a mechanism beginning with the cathodic reduction of Ni­(II) to generate a Ni(0) complex. This Ni(0) species undergoes oxidative addition to form the aryl alkynyl iodide, forming a Ni intermediate (**A**). This intermediate then undergoes a 5-exo-dig cyclization, leading to a cyclized vinyl nickel intermediate (**B**). Subsequent nucleophilic addition of benzaldehyde to the vinyl nickel intermediate forms a nickel alkoxide (**C**). This alkoxide is transmetalated with Al­(III), which is produced at the anode via oxidation, releasing Ni­(II). The Ni­(II) is then reduced at the cathode to sustain the catalytic cycle.

**Scheme 51:**
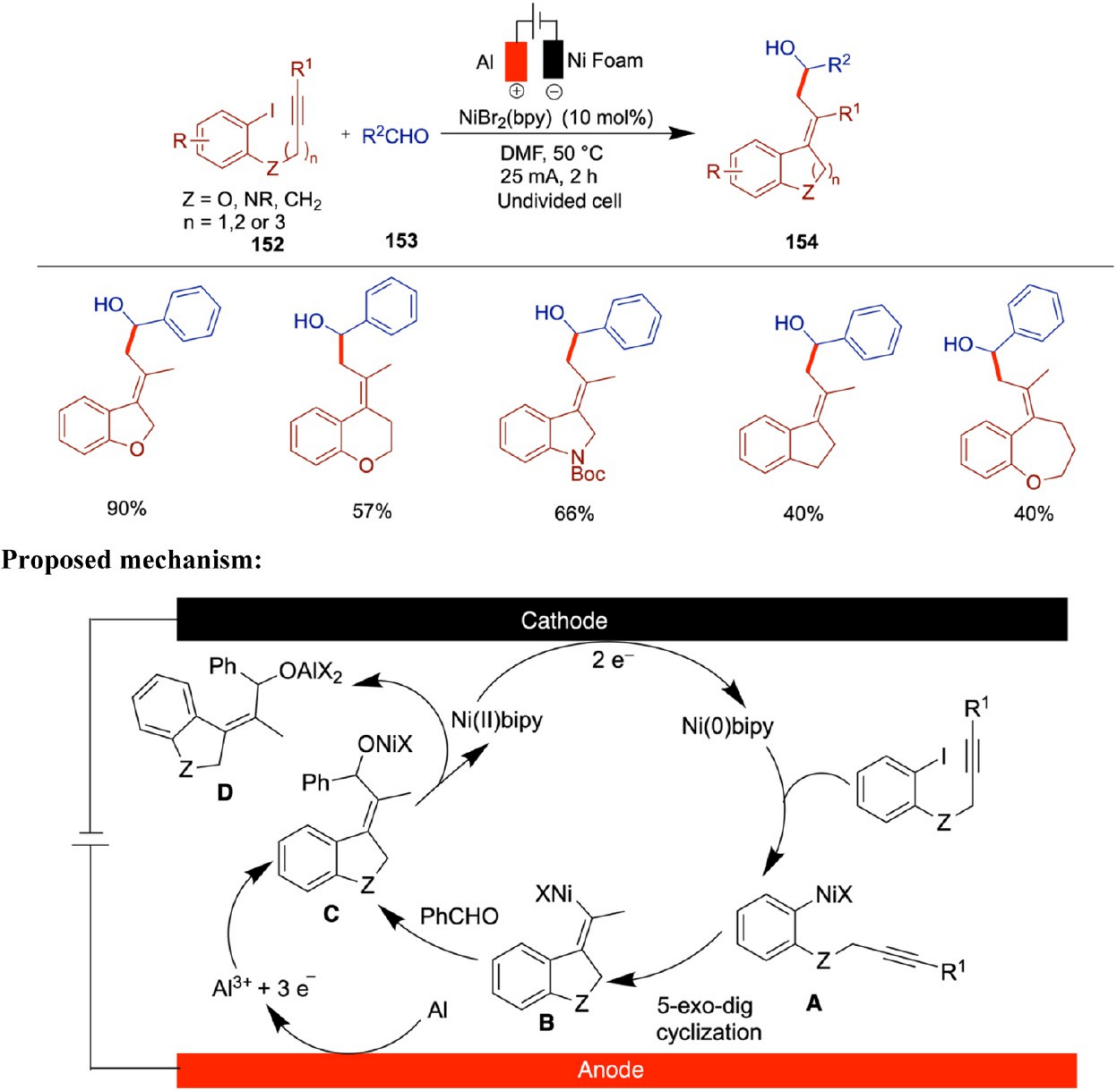
Intramolecular Nickel-Catalyzed Electrochemical Cyclization of Alkynyl Aryl Iodides

## Nickel-Catalyzed Electrochemical C–H Activation Reactions

Electrochemical C–H activation reactions offer a valuable opportunity to transform inactive C–H bonds into various functional groups, and numerous studies have been reported using different transition-metal catalysts.^117,118^ In this Perspective, we focus on nickel-catalyzed electrochemical C–H functionalizations, highlighting key developments in the field. Notably, in 2020, Ackermann et al. reported C–H alkoxylation with secondary alcohols via oxidatively induced reductive elimination from a nickel­(III) intermediate (Scheme 52).^119^ Various amides with different substitutions can react with sterically hindered secondary alcohols to produce ether derivatives. This approach can be further extended to functionalize complex secondary alcohols.

**Scheme 52:**
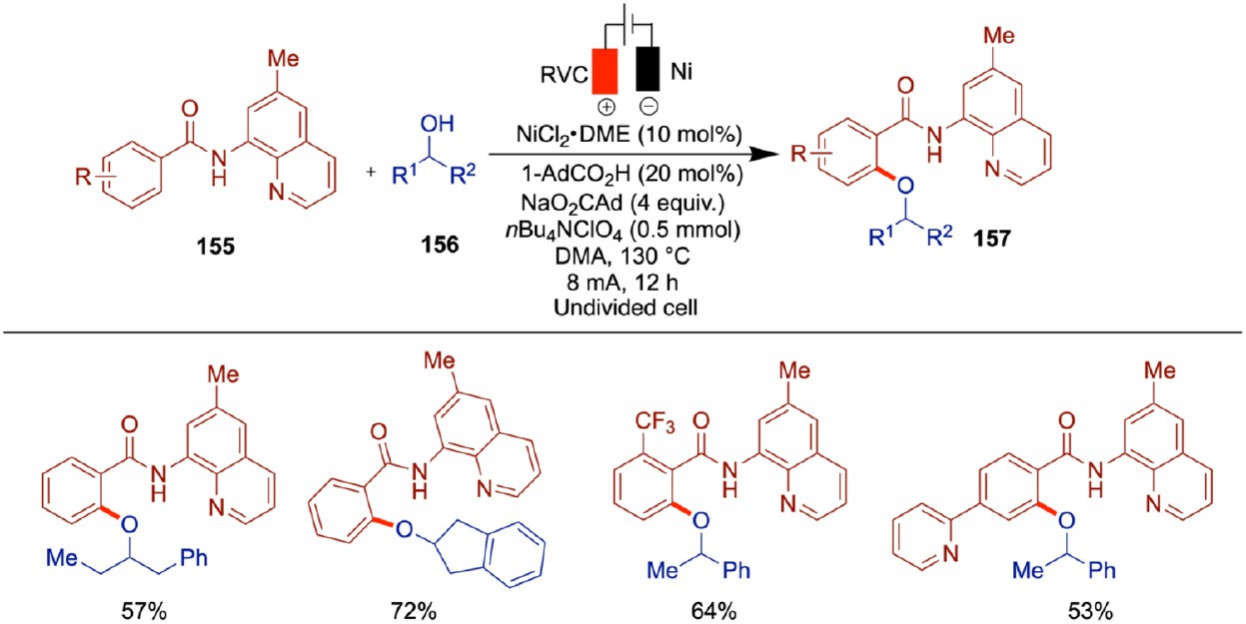
Nickel-Catalyzed Electrochemical C–H Oxygenation

In 2018, Ackermann et al. reported a nickel-catalyzed electrooxidative C–H amination with a wide scope with respect to amines and amides (Scheme 53).^120^ Although copper-catalyzed amination has been reported before, the nickel-catalysis achieved with this reaction provided a mild and efficient method for the C–N bond formation reaction. Improved yields were obtained using the NiCl_2_·DME complex compared to Ni­(OAc)_2_·4H_2_O, likely due to the enhanced solubility of the former in organic solvents, which facilitates better catalytic performance. The authors proposed a mechanism in which a Ni­(II) complex is formed from the amide and the nickel catalyst. This complex undergoes ligand exchange with an amine, resulting in aminated nickel complex **B**. Complex **B** then undergoes anodic oxidation to form Ni­(III) complex **C**, which is further oxidized anodically to generate Ni­(IV) complex **D**. The Ni­(IV) complex then undergoes reductive elimination, producing the desired product and regenerating the catalyst, thereby continuing the catalytic cycle.

**Scheme 53:**
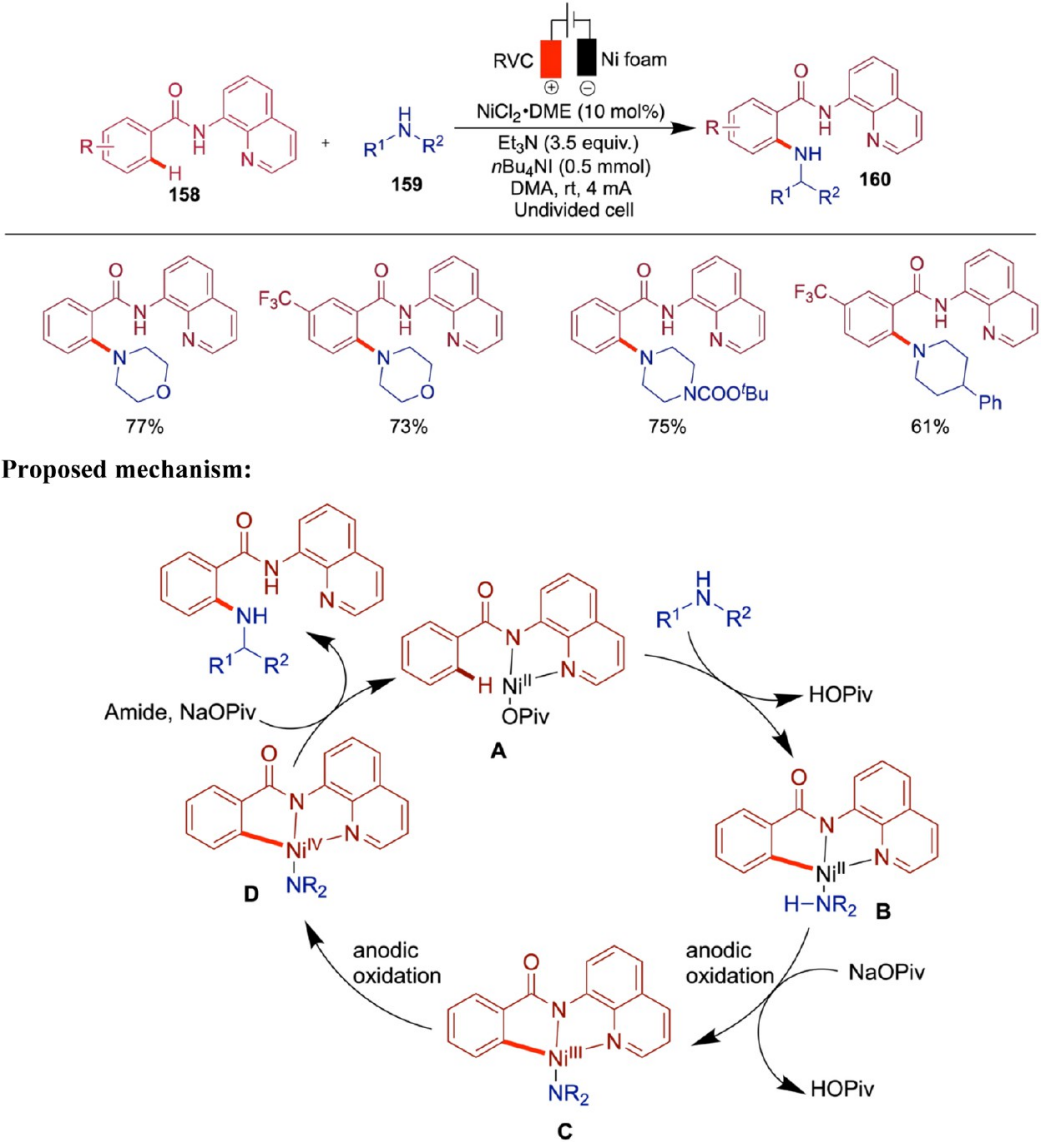
Nickel-Catalyzed Electrochemical Amination

Ackermann et al. reported a nickel-catalyzed mild C–H alkylation of amides at room temperature using triethylamine as a mild base (Scheme 54).^121^ The selection of electrodes and solvents was found to significantly influence the reaction efficiency. Specifically, a zinc anode paired with a nickel cathode in an undivided cell provided the best results, affording the C/H alkylated product in 76% yield. In contrast, alternative electrode combinations, including graphite felt and nickel foam, were less effective. Among solvents, DMA delivered optimal performance, DMF gave slightly lower yields, and methanol proved considerably less effective. Various alkyl halides can be incorporated, although only iodides are effective. Despite this limitation, the mild reaction conditions make this electrochemical alkylation a viable alternative to traditional methods, which typically require temperatures of 160 °C.^122,123^ A wide range of substituents on both amides and alkyl halides were tolerated, producing alkylated products in good to moderate yields. Mechanistic studies suggest that Ni­(II/III/I) catalytic species may be generated during the reaction, facilitating the alkylation process.

**Scheme 54:**
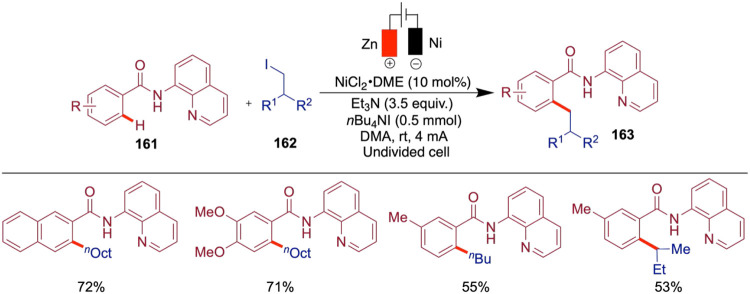
Nickel-Catalyzed Electrochemical C–H Alkylation

## Nickel Electrocatalysis under Flow Conditions

Very recently, the Nol group reported an elegant continuous-flow electroreductive nickel-catalyzed cyclopropanation of alkenes using gem-dichloroalkanes (Scheme 55).^124^ Cyclopropanes are highly sought-after molecules in organic synthesis, as this structural motif is commonly found in many pharmaceuticals. In this article, the authors combined electricity and continuous flow for the cyclopropanation reaction, achieving a broad substrate scope and successfully accommodating both electron-rich and electron-poor alkenes. Through mechanistic studies, the key nickel-carbene intermediate, generated electrochemically, was identified. An interesting observation was made when cyclic enones were subjected to the standard conditions: instead of the anticipated cyclopropanes, β-methylated products were obtained. The authors have proposed the following mechanism for the Ni-electrocatalytic cyclopropanation, which begins with the initial reduction of NiBr_2_-^i^Pr-PyBOX (**A**) at the cathode, producing Ni^0^-^i^Pr-PyBOX (**B**). The nickel-carbene intermediate (**C**) is generated through a second reduction in the presence of dichloromethane. At this stage, there are two possible pathways for the newly formed nickel-carbene complex (**C**): it can either undergo homocoupling or proceed toward the expected cyclopropanation. In the latter case, a nickelacyclobutane intermediate (**D**) is formed through interaction with the alkene substrate. The cyclopropanation product is obtained via reductive elimination, which regenerates complex **B**, completing the catalytic cycle.

**Scheme 55:**
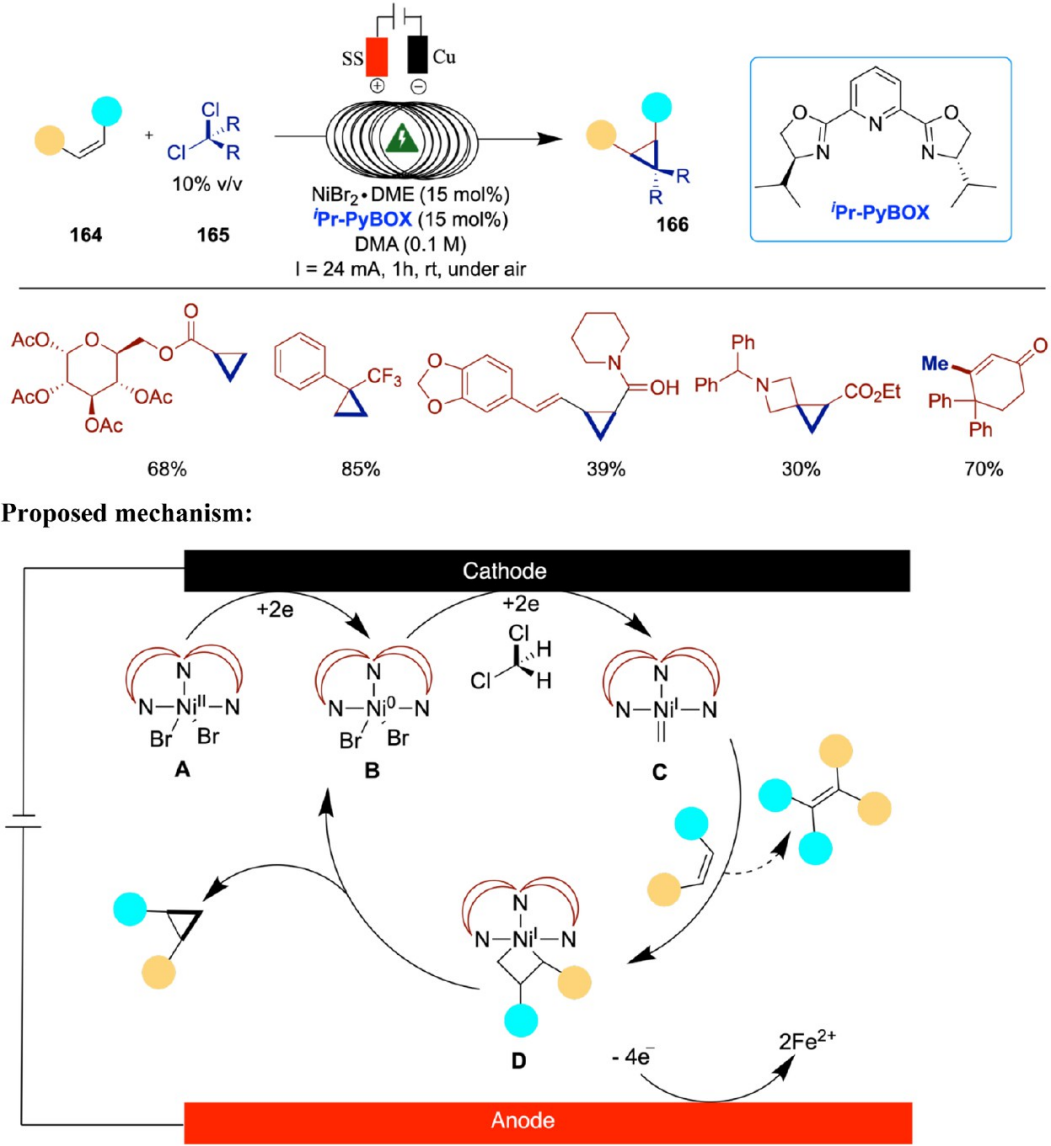
Nickel-Catalyzed Electrochemical Cyclopropanation

## Nickel-Catalyzed Dicarbofunctionalization of Alkenes

Catalytic dicarbofunctionalization of alkenes has been well-established by using nickel catalysis under conventional conditions. The appeal of these transformations lies in the use of diverse coupling partners for alkene functionalization, thereby enhancing their synthetic utility. In 2022, Xia and co-workers reported a nickel-catalyzed reductive dicarbofunctionalization of vinylarenes enabled by an electrochemical process (Scheme 56).^125^ In this reaction, the authors demonstrated broad substrate compatibility by evaluating various aryl iodides, styrene derivatives, and alkyl iodides under optimized electrochemical conditions. Aryl iodides bearing electron-withdrawing groups such as formyl, ketone, ester, cyano, halide, trifluoromethyl, and boronate esters at the para-position furnished the desired products in moderate to good yields. Electron-donating or neutral aryl iodides also reacted, albeit with a lower efficiency. The reaction was tolerant of ortho- and metasubstitution as well. A range of styrene derivatives, including those with electron-donating and -withdrawing groups, participated effectively, and even heteroaromatic alkenes like 2-vinylthiophene afforded the product, though in lower yield. In the case of alkyl iodides, cyclic and primary iodides were tolerated, while tertiary iodides gave poor yields. Importantly, the methodology enabled late-stage modification of complex molecules, including a steroid derivative, highlighting its potential in medicinal chemistry.

**Scheme 56:**
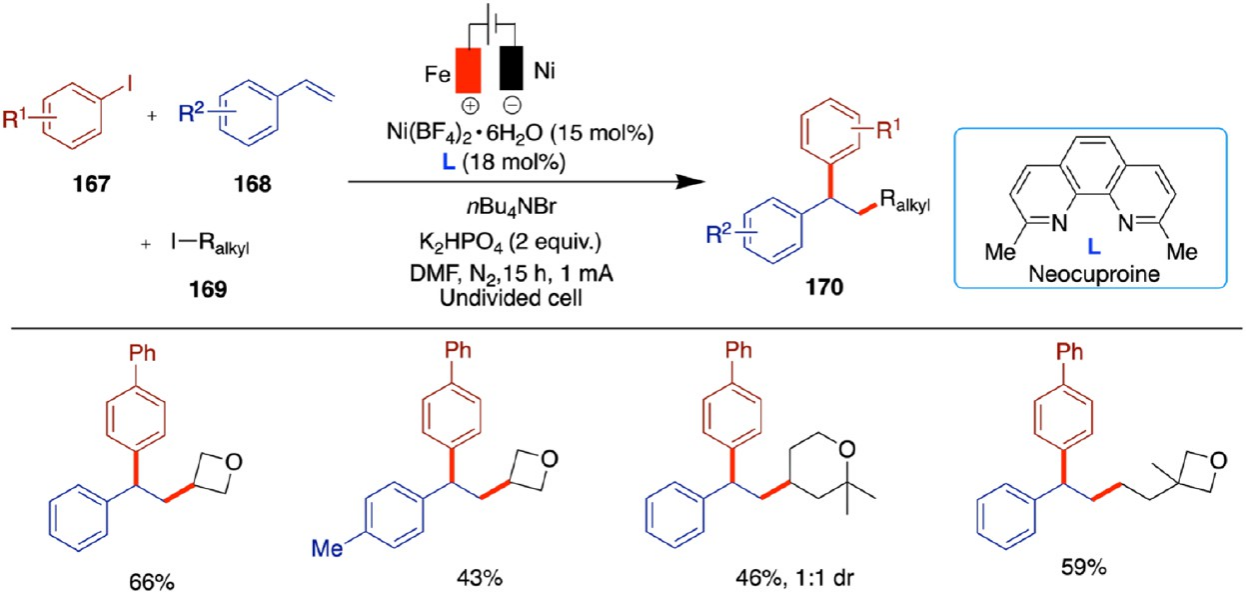
Electrochemical Dicarbofunctionalization

## Future Directions

Further exploration of the underlying mechanisms in nickel-catalyzed electrochemical reactions will be crucial for optimizing existing processes and developing new ones. Detailed mechanistic studies, supported by advanced computational methods, could uncover new catalytic cycles and reaction pathways, leading to more efficient and selective transformations. Expanding the range of substrates that can be utilized in nickel-catalyzed electrochemical cross-coupling is another important area for future research. By broadening the scope of compatible functional groups and molecular architectures, these methods can be applied to a wider array of synthetic challenges.

While the potential of nickel-catalyzed electrochemical cross-coupling is clear, translating these methods from the laboratory to an industrial scale will require further development. Research focused on improving the scalability and economic viability of these processes, such as optimizing electrochemical cell design and reducing catalyst loadings, will be essential for their widespread adoption in industry. The integration of electrochemical techniques with flow chemistry presents an exciting opportunity for the continuous, scalable synthesis of complex molecules. This combination could lead to highly efficient automated processes that are both cost-effective and environmentally friendly. Finally, the discovery of new nickel-based catalytic systems with enhanced activity, selectivity, and stability will continue to propel the field forward. The development of catalysts capable of operating under even milder conditions or exhibiting unique reactivity could open entirely new avenues in synthetic chemistry.

## Conclusions

Nickel-catalyzed electrochemical cross-coupling has rapidly evolved into a powerful and sustainable platform in modern synthetic chemistry, uniquely merging the catalytic versatility of nickel with the precision and tunability of electrochemical methods. This approach enables the efficient construction of C–C and C–heteroatom bonds under mild conditions while minimizing reliance on stoichiometric oxidants or reductants. Innovations in reaction design, such as cross-electrophilic coupling, enantioselective catalysis, and C–H activation, have significantly expanded the synthetic utility and mechanistic landscape of this emerging field.

Despite these advances, realizing the full environmental and practical potential of nickel electrocatalysis demands that several persistent limitations. A critical bottleneck lies in the continued reliance on polar aprotic solvents such as *N*,*N*-dimethylformamide (DMF) and *N*-methyl-2-pyrrolidone (NMP). While effective in supporting electrochemical efficiency and catalyst stability, these solvents pose environmental and safety concerns that conflict with the core tenets of green chemistry. Efforts to replace them with greener alternatives often lead to compromised reaction performance, underscoring the need for further optimization of the solvent systems.

Moreover, many current methods exhibit limited substrate generality and functional group tolerance, which restricts their broader applicability. To overcome this, future research should focus on the development of universally applicable protocols that perform consistently across diverse electrophilic and nucleophilic partners. Equally important is the innovation of new ligand architectures. Given the critical role ligands play in dictating reactivity, selectivity, and substrate compatibility, the design of modular, tunable ligands holds significant promise for enhancing catalytic efficiency and scope.

In parallel, practical considerations must not be overlooked. The potential toxicity and immunogenicity of nickel, particularly relevant in pharmaceutical or large-scale contextsnecessitate the integration of strategies for catalyst recovery, purification, and, where possible, substitution with less toxic alternatives. Additionally, the environmental and toxicological impact of electrode materials, especially sacrificial anodes such as aluminum or zinc, must be carefully considered. The use of such anodes generates metal byproducts (e.g., Zn^2+^ or Al^3+^), contributing to reagent waste and complicating downstream purification. This is particularly critical in pharmaceutical applications, where trace metal impurities are tightly regulated and the overall environmental footprint must be minimized. Future developments should aim to reduce or replace sacrificial anodes through the use of recyclable or inert alternatives or through electrochemical setups that enable closed-loop metal usage, in which the metal used is not consumed and discarded but instead recovered and reused within the system.

Looking forward, nickel-catalyzed electrochemical cross-coupling is poised to transition from a cutting-edge research domain into a cornerstone of sustainable synthesis in both academic and industrial settings. This evolution will depend not only on the continued exploration of nickel’s unique redox capabilities but also on the field’s commitment to balancing reactivity with responsibility. Through strategic advances in solvent use, ligand design, and reaction generality, nickel electrocatalysis can fulfill its potential as a truly green and transformative synthetic methodology.

In summary, while nickel-catalyzed electrochemical cross-coupling has demonstrated remarkable progress and potential as a green synthetic platform, realizing its full impact will depend on targeted efforts in three critical areas: (1) reducing reliance on environmentally hazardous solvents through the adoption of greener media, (2) advancing ligand design to improve efficiency, selectivity, and functional group tolerance, and (3) developing broadly applicable methodologies that maintain high performance across diverse substrates and reaction types.

## Data Availability

No new data were generated or analyzed in support of this study.
